# Heterotrimeric G proteins regulate planarian regeneration and behavior

**DOI:** 10.1093/genetics/iyad019

**Published:** 2023-02-10

**Authors:** Jennifer E Jenkins, Rachel H Roberts-Galbraith

**Affiliations:** Department of Cellular Biology, University of Georgia, Athens, GA 30602, USA; Department of Cellular Biology, University of Georgia, Athens, GA 30602, USA

**Keywords:** planarian, regeneration, heterotrimeric G protein, GPCR, signaling, behavior, neurobiology, flatworm, *Schmidtea*

## Abstract

G protein-coupled receptors play broad roles in development and stem cell biology, but few roles for G protein-coupled receptor signaling in complex tissue regeneration have been uncovered. Planarian flatworms robustly regenerate all tissues and provide a model with which to explore potential functions for G protein-coupled receptor signaling in somatic regeneration and pluripotent stem cell biology. As a first step toward exploring G protein-coupled receptor function in planarians, we investigated downstream signal transducers that work with G protein-coupled receptors, called heterotrimeric G proteins. Here, we characterized the complete heterotrimeric G protein complement in *Schmidtea mediterranea* for the first time and found that 7 heterotrimeric G protein subunits promote regeneration. We further characterized 2 subunits critical for regeneration, *Gαq1* and *Gβ1-4a*, finding that they promote the late phase of anterior polarity reestablishment, likely through anterior pole-produced Follistatin. Incidentally, we also found that 5 G protein subunits modulate planarian behavior. We further identified a putative serotonin receptor, *gcr052*, that we propose works with *Gαs2* and *Gβx2* in planarian locomotion, demonstrating the utility of our strategy for identifying relevant G protein-coupled receptors. Our work provides foundational insight into roles of heterotrimeric G proteins in planarian biology and serves as a useful springboard toward broadening our understanding of G protein-coupled receptor signaling in adult tissue regeneration.

## Teaser

In this manuscript, Jenkins and Roberts-Galbraith use a forward genetics screen to query the functions of heterotrimeric G proteins in planarians as a starting point for understanding potential roles of G-protein coupled receptors in planarian regeneration. They find several heterotrimeric G protein subunits with important roles in regeneration, with one alpha and beta pair important for resetting body polarity after injury. Incidentally, they also describe several heterotrimeric G protein subunits with roles in planarian behavior and movement.

## Introduction

**Fig. 1.**
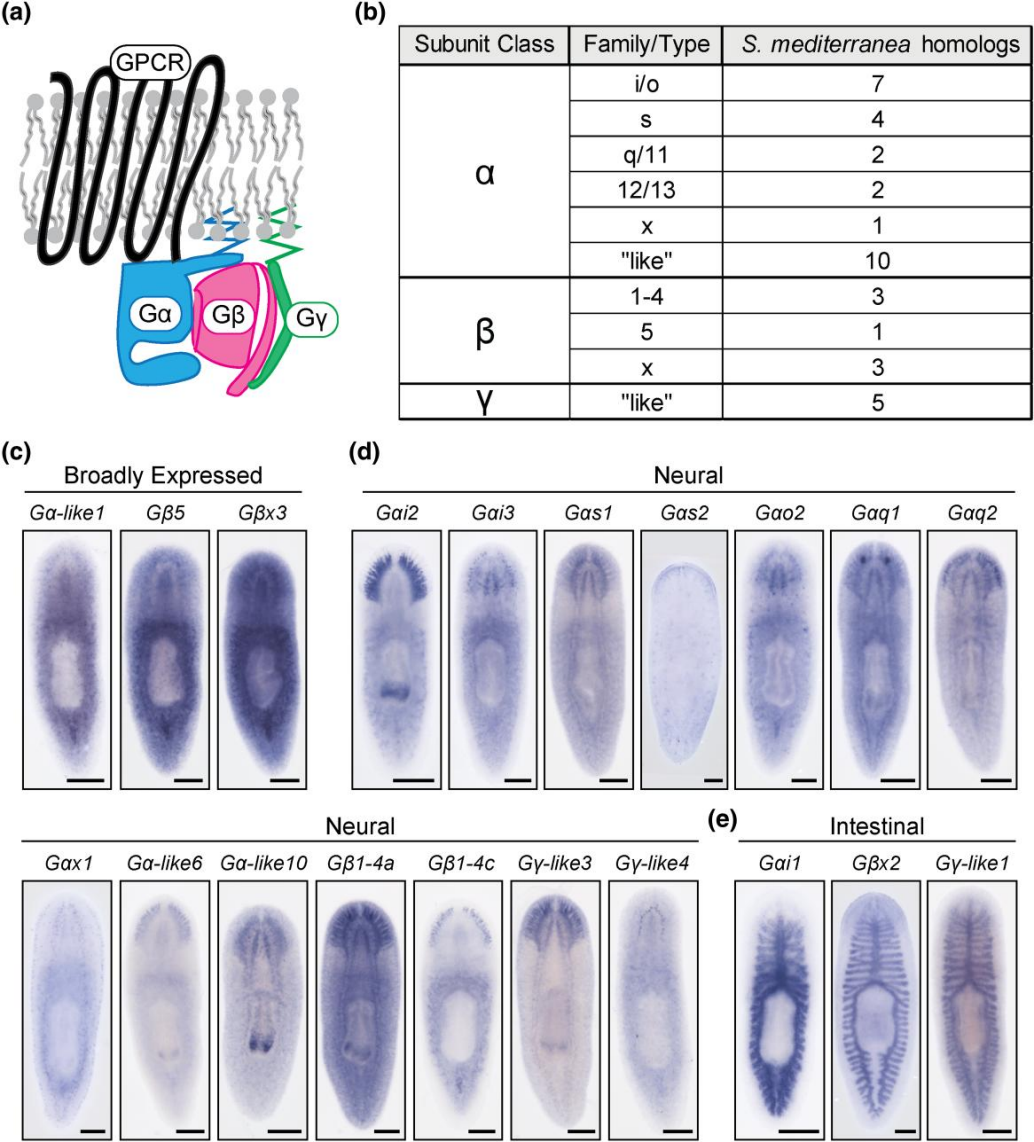
Planarians possess diverse heterotrimeric G proteins. a) Graphical summary of a typical heterotrimeric G protein complex associated with a GPCR (top in the lipid bilayer). The heterotrimer is composed of Gα (bottom left), Gβ (bottom middle), and Gγ (bottom right) subunits that are activated upon ligand binding to the receptor. b) Table depicting *S. mediterranea* homologs for heterotrimeric G protein subunits. Representative images of G protein subunit expression patterns categorized by the most visually enriched tissue type into broad (c), neural (d), and intestinal (e) patterns. Scale bars = 200 μm. The anterior of the animals is oriented toward the top of the page in all figures.

G protein-coupled receptors (GPCRs) represent one of the largest, most highly conserved, and functionally diverse families of cell surface receptors (Anantharaman *et al*. 2011; Krishnan *et al*. 2012; Langenhan *et al*. 2015). GPCRs also comprise ∼30% of drug targets, due to their broad involvement in cell signaling (Hopkins and Groom 2002; Wise *et al*. 2002; Garland 2013). GPCRs possess structures that include 7 transmembrane domains, extracellular domains for signal perception, and intracellular domains for interaction with signal transducers (Fig. 1a) (Pierce *et al*. 2002; Lagerström and Schiöth 2008). Canonically, activation of a GPCR initiates dissociation of a heterotrimeric G protein complex (Fig. 1a) into an α subunit and a β/γ subcomplex, both of which impact cellular function (Oldham and Hamm 2008; Smrcka 2008).

Importantly, GPCR signaling regulates wound response throughout the animal kingdom in organisms that include nematodes, fruit flies, and mammals (Ziegler *et al*. 2009; Doze and Perez 2013; Kiseleva *et al*. 2014; Zugasti *et al*. 2014; Choi *et al*. 2015; Guo *et al*. 2019; O’Connor *et al*. 2021). For example, the protease-activated receptor 1 (PAR1) GPCR promotes wound healing in murine skin by stimulating production of keratinocytes (Kiseleva *et al*. 2014). Downstream heterotrimeric G proteins also modulate regeneration. Gα class subunits from several families have been shown to promote *or* inhibit axon regeneration in vertebrates (Bates and Meyer 1996; Li *et al*. 2016) and *Caenorhabditis elegans* (Shimizu and Hisamoto 2020). However, roles for GPCR pathways have not yet been explored in organisms that complete robust, whole-body regeneration. Studying GPCR signaling in highly regenerative models could reveal new roles for these pathways in regeneration of complex tissues.

Freshwater flatworms called planarians provide an appealing model for investigation of mechanisms underlying robust regeneration. After nearly any injury, planarians produce a blastema in which differentiating cells accumulate and mature to reconstruct missing structures (Baguna *et al*. 1989). Regeneration proceeds through key events that include wound detection (Wenemoser *et al*. 2012; Wurtzel *et al*. 2015), activation of pluripotent adult stem cells (Wagner *et al*. 2011; Raz *et al*. 2021), and polarity reestablishment (Witchley *et al*. 2013; Reddien 2018). Through these processes, planarians regenerate all tissues and complex organs de novo, including the brain. How planarian cells detect injury, reinterpret polarity axes, and mount the correct regenerative response after injury remain key areas of investigation. Because regeneration requires multifaceted, fine-tuned coordination of cellular responses after injury and because GPCR signaling functions in diverse aspects of cell biology and healing in other animals, we hypothesized that GPCR pathways play key roles in planarian regeneration that have yet to be discovered.

Currently, the genome of *S. mediterranea* is predicted to contain 566 GPCR-encoding genes (Zamanian *et al*. 2011; Saberi *et al*. 2016). Very few of these genes are functionally characterized, with the identified GPCRs promoting posterior identity, supporting planarian locomotion, coordinating germline differentiation and maintenance, facilitating reproductive system development and repair, or impacting eye regeneration (Zamanian 2011; Lozano 2015; Saberi *et al*. 2016; Pascual-Carreras *et al*. 2021). Characterization of planarian heterotrimeric G protein subunits is also limited. *gpas* is expressed in the brain branches and pharynx (Cebrià *et al*. 2002; Iglesias *et al*. 2011), while 4 other G protein subunit genes (*gna-q*, *gna-o*, *gnb*, and *gnc*) are highly expressed in photoreceptors (Lapan and Reddien 2012). Work among planarian species assessed function for a handful of specific GPCR/G protein pathways (Zamanian 2011; Zamanian *et al*. 2012; Chan *et al*. 2015; Chan *et al*. 2016). However, a comprehensive analysis of G protein function could help indicate the extent to which GPCR pathways regulate tissue regeneration and help uncover new roles for GPCR pathways in planarians.

As an essential first step toward pursuing our hypothesis that GPCR signaling promotes regeneration, we characterized heterotrimeric G proteins in the planarian *S. mediterranea*. In this work, we identified and characterized 38 predicted heterotrimeric G protein subunit-encoding genes, which include highly conserved homologs of described vertebrate G protein families and divergent subunits. We show that 7 G protein subunit-encoding genes—*Gαs1*, *Gαs2*, *Gαq1*, *Gαq2*, *Gαo2*, *Gα-like6*, and *Gβ1-4a*—promote planarian regeneration. Two of the identified genes, *Gαq1* and *Gβ1-4a*, are essential for promoting the late phase of anterior–posterior axis reestablishment, likely by influencing production of *follistatin^+^* anterior pole cells. We also show that 5 genes—*Gαs1*, *Gαs2*, *Gαq1*, *Gβ1-4a*, and *Gβx2*—are required for planarian movement. To illustrate the utility of our G protein-centered approach to identifying key GPCRs, we further identified a GPCR-encoding gene, *gcr052* (Saberi *et al*. 2016), as a potential partner of *Gαs2* and *Gβx2*. Taken together, our results reveal new functions for heterotrimeric G protein signaling in the highly regenerative planarian model. Our data further provide a much-needed starting point for identifying GPCRs with roles in regeneration.

## Materials and methods

### Animal maintenance

Planarians from an asexual strain of the species *S. mediterranea* [CIW4 (Alvarado *et al*. 2002)] were kept in 1X Montjuïc salts [1.6-mM NaCl, 1-mM CaCl_2_, 1-mM MgSO_4_, 0.1-mM MgCl_2_, 0.1-mM KCl, and 1.2-mM NaHCO_3_ prepared in ELGA PURELAB (ELGA LabWater, Woodridge, IL) ultrapure water] (Cebrià and Newmark 2005) at 18°C in the dark. Animals were fed beef liver puree weekly or biweekly. Animals were cut periodically to expand their numbers and generate properly sized (∼2–5 mm) individuals for experiments. Animals were starved for a minimum of 1 week before experiments.

### Gene identification

Gα subunit-like transcripts were mined using the guanine nucleotide-binding domain [PF00503 (Coleman *et al*. 1994)], Gβ subunit-like transcripts were mined using the WD40-repeat-containing domain preceded by N-terminal alpha helix [IPR001632 (Wall *et al*. 1995)], and Gγ subunit-like transcripts were mined using the GGL domain [PF00631 (Snow *et al*. 1998)]. Each relevant functional domain [from Pfam (El-Gebali *et al*. 2019) or InterPro (Mitchell *et al*. 2019)] was searched within the translated *S. mediterranea* transcript dataset dd_Smed_v6 (Brandl *et al*. 2016; Rozanski *et al*. 2019); then redundant transcripts were removed. To ensure the retrieved Gγ subunit-like sequences were not regulators of G protein signaling (RGS) proteins, the absence of an RGS domain [PF00615 (Chen *et al*. 2001; Longenecker *et al*. 2001)] was confirmed (Supplementary Table 1).

### Protein alignment and phylogenetic analysis

Amino acid sequences were predicted using the web-based translation tool Swiss ExPASy (Expert Protein Analysis System) Molecular Biology Server (Swiss Institute of Bioinformatics, University of Lausanne, Switzerland) (Gasteiger *et al*. 2003). Protein sequences were aligned to reference sequences from other animals (Supplementary Table 5) using Clustal Omega O (1.2.4) (Sievers and Higgins 2014), and secondary structures were predicted with ESPript3.0 (Robert and Gouet 2014), using well-characterized structure examples (PDB ID: 1GP2). Phylogeny was analyzed using www.phylogeny.fr (Dereeper *et al*. 2008). The “a la carte” option was selected with MUSCLE for alignment (Edgar 2004) and PhyML for construction of the phylogenetic tree (Guindon *et al*. 2010). For the PhyML analysis, 100 bootstrap replicates were performed, and the WAG model of amino acid substitution was applied.

### Molecular cloning

For genes of interest, primers were designed using Primer3 (Rozen and Skaletsky 1999) to amplify an ∼700-bp region of the corresponding gene from asexual *S. mediterranea* cDNA (Supplementary Table 6). PCR products were cloned into the vector, pJC53.2 (Collins *et al*. 2010) using standard molecular biology protocols.

### RNA interference (RNAi) experiments

**Fig. 2.**
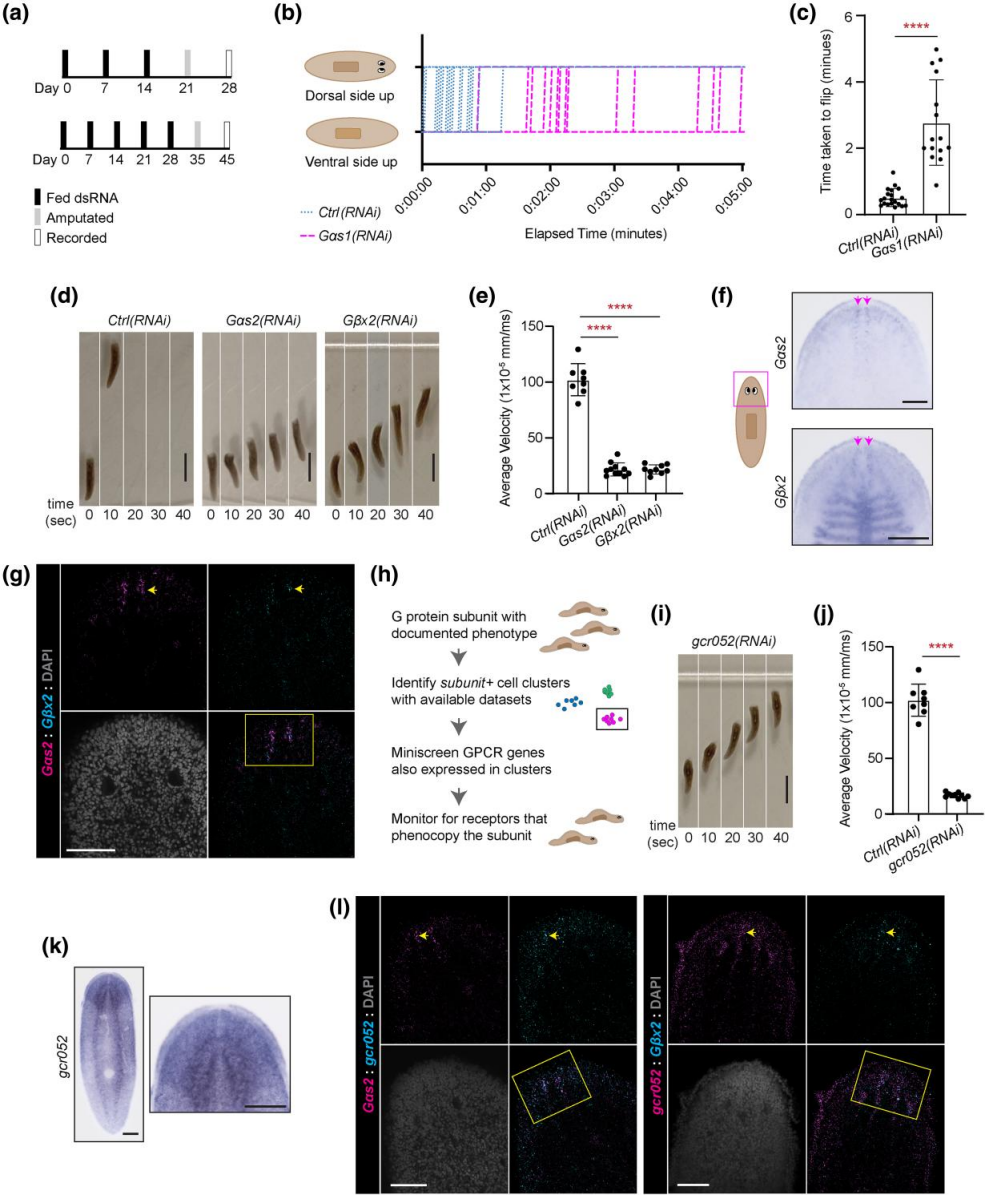
Planarian heterotrimeric G proteins and GCR052 promote animal movement. a) RNAi paradigms used during initial regeneration screens (top) and follow-up, longer-term experiments (bottom). Data from b–c resulted from the top paradigm, and data from d–e and i–j resulted from the bottom paradigm. b) Flip assay used to document paralysis in *Gas1(RNAi)* animals. The graph includes flipping data for 20 animals per RNAi condition. c) Bar graph showing times taken for animals to flip over to a correct ventral-down posture (excluding 5 nonflipping *Gas1(RNAi)* animals), displayed as mean and standard deviation. Differences were analyzed with unpaired *t*-test with Welch's correction. **** = *P* value ≤ 0.0001. d) Image stills from videos capturing locomotion displayed by regenerating control, *Gas2(RNAi)*, and *Gβx2(RNAi)* animals 10 dpa. e) Results from quantification of average velocity over a 40-s timespan in regenerating control, *Gas2(RNAi)*, and *Gβx2(RNAi)* animals, displayed as mean and standard deviation. f) Images of *Gas2* and *Gβx2* zoomed colorimetric ISH showing the clusters of cells at the anterior tip of the animals, indicated with arrowheads. g) *Gas2* and *Gβx2* dFISH images of the head region. Arrowhead indicates an example of a cell enriched with both transcripts. The box indicates the region of interest where the anterior clusters are found. h) Graphical scheme showing the method used to identify candidate GPCRs for G protein subunits with documented phenotypes. i) Image stills from videos capturing locomotion displayed by regenerating *gcr052(RNAi)* animals. j) Results from quantification of average velocity over a 40-s timespan in intact control and *gcr052(RNAi)* animals, displayed as mean and standard deviation. Data displayed in i–j are from the same experiment as shown in d–e. Differences in average velocities were analyzed with Brown–Forsythe and Welch ANOVA with multiple comparisons. ****= *P* value ≤ 0.0001. k) Images showing the expression pattern of *gcr052* through colorimetric ISH. l) *gcr052* dFISH images with *Gas2* or *Gβx2* in the head region. The box indicates the region of interest where the anterior clusters are found. Arrowheads indicate an example of a cell enriched with both transcripts. Scale bars in d and i = 2 mm. Scale bars in f and d = 200 μm. Scale bars in g and l=100 μm.

The dsRNA was transcribed in vitro from PCR products amplified from pJC53.2 using standard molecular methods (Collins *et al*. 2010; Rouhana *et al*. 2013). Concentration of dsRNA was determined using either a NanoPhotometer NP80 (Implen, Munich, Germany) or by band intensity after gel electrophoresis. For a typical experiment, 10–12 animals were fed 1–3-µg dsRNA mixed in ∼30-µL food (beef liver paste, 4:1 liver:salts mixture), and 1-µL green food dye was added to verify that the animals ate. The mixture was doubled for larger experiments. Negative control worms were fed dsRNA matching green fluorescent protein (*GFP*) or bacterial genes [chloramphenicol resistance gene (*Cm^R^*) and toxin CcdB (*ccdB*)]. Animals were kept in 60–100-mm Petri dishes. After eating, the animals were washed and transferred to fresh dishes, and salts were supplemented with 1:1000 gentamicin sulfate [50-mg/mL stock (Gemini Bio, West Sacramento, CA)]. Animals were fed dsRNA ∼once per week for 3 total feedings [more feedings given in long-term RNAi experiments (Fig. 2; Supplementary Fig. 6)] and then were processed. Live images during experiments were obtained using a Zeiss Axiocam 506 color camera mounted on a Zeiss Axio Zoom.V16 microscope (ZEISS Microscopy, Jena, Germany). Live images and video were also captured on an iPhone 6 and/or SE and processed in iMovie (Apple Inc., Cupertino, California).

### Behavior assays

For the flipping assay (Fig. 2b–c), live recordings were captured for up to 5 min after each animal was put on its dorsal side. We observed how long it took each animal to flip to its ventral side. For locomotion studies (Fig. 2; Supplementary Fig. 3), animals were recorded in 13 × 13-mm/square grid dishes (VWR International, Radnor, PA) for at least 15 min. Velocity was quantified for 8–12 individual animals, while they showed forward movement over at least a 40-s timespan. Distance was tracked using BioTracker (Mönck *et al*. 2018); then velocity was calculated for intervals of 4 s. Average velocities were determined from the values of 11 successive intervals. For negative phototaxis assays (Supplementary Fig. 3D), animals were put in 13 × 13-mm/square grid dishes (VWR International, Radnor, PA) with lids half-covered with black electrical tape. This produced an uncovered/light side and covered/dark side of the dish. Animals were placed in the far-left corner of the uncovered region and then recorded for at least 10 min. For each 60-s interval, the number of animals visible in the uncovered region was documented.

### In situ hybridization (ISH)

Single-stranded antisense riboprobes were transcribed with digoxigenin (Dig-11-UTP) (Sigma-Aldrich, St. Louis, MO) using standard molecular methods (Collins *et al*. 2010). Animals were fixed, hybridized with riboprobes, and stained as previously described (King and Newmark 2013), with the following modifications: animals were killed in a 10% N-acetyl cysteine solution and treated with a 2-µg/ml Proteinase K solution. Regenerating animals were treated with the Proteinase K/postfixation steps (as opposed to a boiling step). After the hybridization step, 56°C washes were as follows: one 20-min wash in wash hyb [25% formamide, 3.5X SSC (0.15-M NaCl, 0.015-M Na citrate), 0.1% Triton X-100, and pH 7.0], three 20-min 2X SSCx (2X SSC and 0.1% Triton X-100) washes, and four 20-min 0.2X SSCx (0.2X SSC and 0.1% Triton X-100) washes. We also replaced MABT with TNTx (0.1-M Tris pH 7.5, 0.15-M NaCl, and 0.3% Triton X-100). After antibody incubation, animals were washed in TNTx for 5 min (1 wash), 10 min (1 wash), and 20 min (6 washes). The fixation step after sample development was omitted. Other key reagents include antidigoxigenin conjugated with an alkaline phosphatase (anti-Dig-AP 1:2000 dilution), nitro blue tetrazolium (NBT), and 5-bromo-4-chloro-3′-indolylphosphate (BCIP) (all Sigma-Aldrich, St. Louis, MO). Animals were mounted in 80% glycerol and imaged with a Zeiss Axiocam 506 color camera mounted on a Zeiss Axio Zoom.V16 microscope (ZEISS Microscopy, Jena, Germany).

### Fluorescent ISH (FISH)

Single-stranded antisense riboprobes were synthesized with digoxigenin (Dig-11-UTP) (Sigma-Aldrich, St. Louis, MO), fluorescein isothiocyanate (FITC-12-UTP) (Roche, Basel, Switzerland), or 2,4-dinitrophenol (DNP) (PerkinElmer, Inc., Waltham, MA) using standard molecular methods (Collins *et al*. 2010). Riboprobes were detected using anti-Dig-POD (1:1000; Sigma-Aldrich, St. Louis, MO), anti-FITC-POD (1:1000; Sigma-Aldrich, St. Louis, MO), or anti-DNP-HRP (1:3000; Vector Laboratories, Newark, CA). Tyramide conjugate signal amplification was performed as previously described (King and Newmark 2013). The final incubation was with DAPI (10 µg/ml) (1:1000; Thermo Fisher Scientific, Waltham, MA). Animals were mounted in VECTASHIELD (Vector Labs, Burlingame, CA) for imaging.

### Immunofluorescence (IF)

Immunofluorescence was adapted from existing protocols (Forsthoefel *et al*. 2014; Ross *et al*. 2015). Planarians were killed in 2% HCl for 5 min with alternating 1-min incubations on ice and gently inverting at room temperature. The HCl step was followed by three 5-min washes in PBS (phosphate-buffered saline: 137-mM NaCl, 2.7-mM KCl, 10-mM Na_2_HPO_4_, 2-mM KH_2_PO_4_, pH 7.4) at room temperature. Animals were then fixed for 15 min in 4% formaldehyde solution in PBS and then shaken in PBSTx (PBS and 0.3% Triton X-100) for 10 min 3 times at room temperature. The animals were bleached under light overnight in 6% H_2_O_2_ in PBSTx. Bleaching was followed by two 10-min PBSTx washes at room temperature. Animals were then blocked [PBSTx and 1% bovine serum albumin (Jackson ImmunoResearch Laboratories, Inc., West Grove, PA)] for at least 4 h. Blocking solution was replaced with a solution containing primary antibody anti-phospho-histone H3 (Ser10) [1:1600 (Cell Signaling Technology, Danvers, MA)] to mark cells in the process of mitosis and were incubated gently shaking at 4°C overnight. The next day animals were incubated in PBSTx at room temperature 8 times for 30 min. Then animals were incubated in blocking solution for 1 h. Blocking solution was replaced with a solution containing the secondary antibody, goat-antimouse IgG + IgM-horseradish peroxidase [1:1000 (Sigma-Aldrich, St. Louis, MO)], and animals were shaken gently at 4°C overnight. Afterward, animals were washed for 30 min 8 times in PBSTx at room temperature. The samples were then shaken for 30 min at room temperature in PBSTi (PBSTx and 10-mM imidazole) wrapped in foil (foil remained until mounting). The samples were then developed for 5 min through tyramide signal amplification (TSA reaction) using FITC-tyramide (1:1000 in PBSTi and 0.015% H_2_0_2_). The samples were then shaken at room temperature in PBSTx 3 times for 10 min and then 2 times for 30 min. The final incubation was in DAPI solution (0.5 µg/ml in PBSTx) overnight at 4°C. Samples were then mounted in VECTASHIELD (Vector Labs, Newark, CA) for imaging.

### Confocal image acquisition

**Fig. 3.**
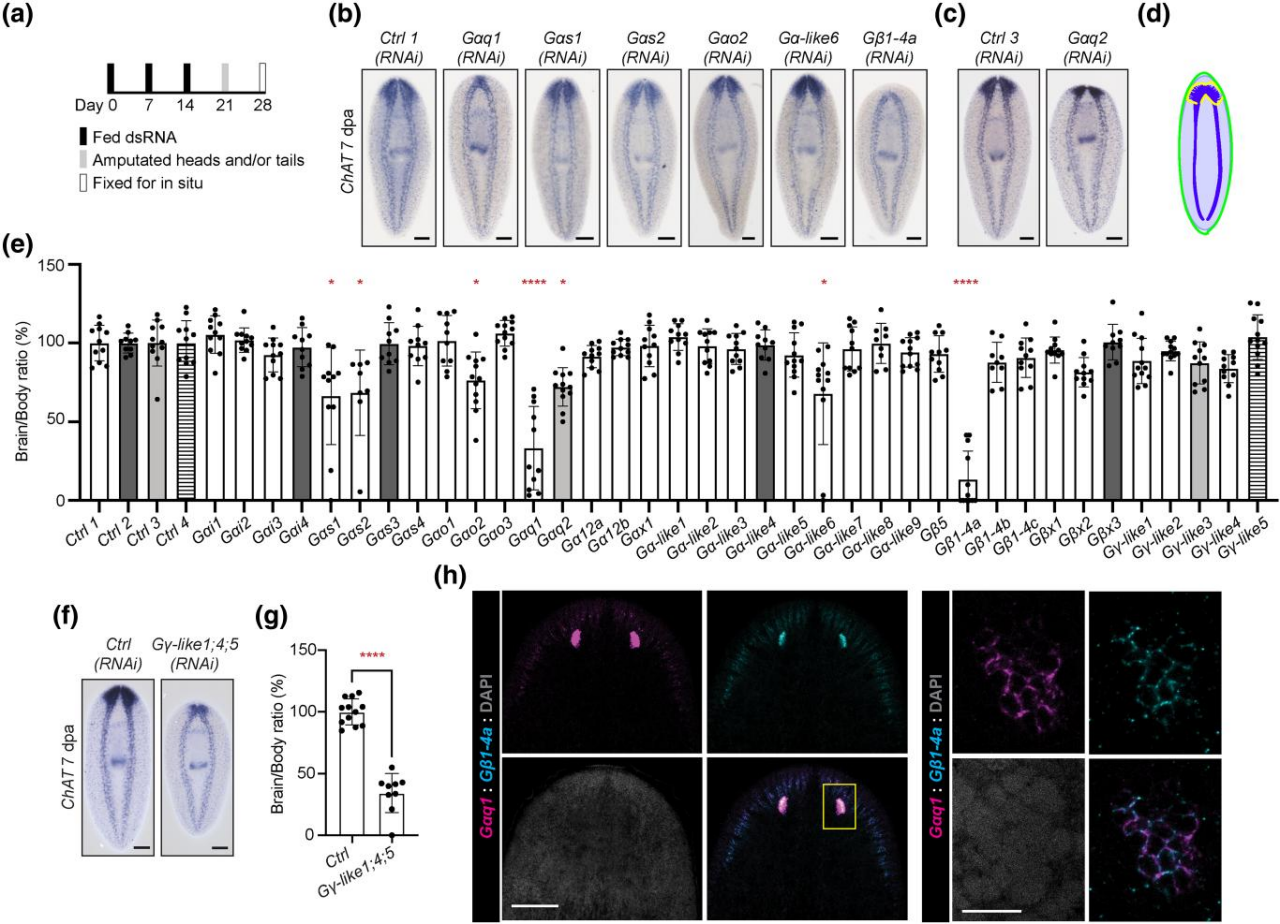
Specific planarian heterotrimeric G protein genes promote brain regeneration. a) RNAi paradigm used for initial regeneration screens. b and c) Representative images showing animals treated with RNAi targeting genes that reduced brain regeneration along with corresponding controls. d) Visual schematic displaying our method for brain regeneration quantification. From *ChAT* ISH images, the area of the brain and body for each animal are used to calculate brain/body ratios (Roberts-Galbraith *et al.* 2016). e) Bar graph of data from quantification of brain/body ratios after RNAi, displaying mean and standard deviation. Bars are color coded to match samples to controls from the same experiment. Differences were analyzed using Brown–Forsythe and Welch ANOVA. * = *P* value ≤ 0.05. **** = *P* value ≤ 0.0001. f) Representative images showing brain regeneration in control and combinatorial *Gγ-like1*, *Gγ-like4*, and *Gγ-like5 (RNAi)* animals. g) Bar graph of quantified brain/body ratios in Gγ-like combinatorial RNAi, displayed as mean and standard deviation. Differences were analyzed using unpaired *t*-test. **** = *P* value ≤ 0.0001. h) *Gαq1* and *Gβ1-4a* dFISH images focusing on the head. The box indicates the region shown in the next, zoomed image of the eyespot, validating coexpression. Scales in b, c, and f and the head region image in h = 200 μm. Scale in the eyespot image of h = 20 μm.

Confocal images were obtained for FISH and IF samples using Zen Black 2.3 SP1 software on a Zeiss LSM 710 AXIO Observer Z1 inverted microscope or Zeiss 880 Axio Imager Z2 microscope (ZEISS Microscopy, Jena, Germany). The details for FISH images are as follows: Fig. 2g and 2l are single slice images using a 20× objective (numerical aperture [NA] 0.8); the head region images in Fig. 3h are max intensity projections of 10 z-sections (9.72-µm sections) taken with a 10× objective (NA 0.3); the zoomed eyespot images in Fig. 3h are single slices captured with a 40× objective (NA 1.4); Supplementary Fig. 6a are max intensity projections of 12 z-sections (1-µm sections) taken with a 20× objective (NA 0.8); and Supplementary Fig. 6b are single slices taken with a 20× objective (NA 0.8). For H3P IF, images of 4 tiles and ∼30 z-sections (1-µm sections) capturing the anterior half of the animals were taken with a 10× objective (NA 0.3). For post-processing, tiles were stitched with Imaris (Oxford Instruments, Abingdon, United Kingdom) or FIJI (FIJI is just ImageJ, Schindelin *et al*. 2012) software.

### Image quantification

For regeneration assays, areas of the brains (Fig. 3; 6 and Supplementary Fig. 4; 8) or blastemas (Supplementary Fig. 4) were measured from fixed sample images by tracing the structures with FIJI imaging software (Schindelin *et al*. 2012) and normalized as described previously (Roberts-Galbraith *et al*. 2016) (Supplementary Table 4). Brain measurements were traced around the outer boundary of the brain, encompassing the entire structure including the brain branches. For growth assays (Supplementary Fig. 6), animal lengths were measured from live images in FIJI (Schindelin *et al*. 2012). Data were statistically analyzed and visualized using Prism GraphPad Version 7.0 software (GraphPad Software, San Diego, CA). Specific tests employed are found in the corresponding figure legends.

For H3P analysis, Imaris software (Oxford Instruments, Abingdon, United Kingdom) was used for quantification of H3P^+^ cells in the body volume of the anterior half of 4–5 animals per RNAi treatment. The spots function of the software was employed to detect green cells. After automated counting, spots were manually checked and adjusted. The surface function was employed to measure the body volume captured in each z-stack. Mitoses/mm^3^ was calculated from the total number of H3P^+^ cells in a given volume.

### Quantitative reverse transcription polymerase chain reaction

RNA was extracted from animals using TRIzol Reagent (Thermo Fisher Scientific, Waltham, MA) as per the manufacturer's protocol (Liu and Rink 2018). Samples were treated with RQ1 RNase-free DNase (Promega Corporation, Madison, WI) for 15 min at 37°C. cDNA was synthesized from RNA using an iScript kit (Bio-Rad, Hercules, CA). Quantitative reverse transcription polymerase chain reaction (RT-qPCR) reactions were completed using SYBR Green PCR Master Mix (Bio-Rad, Hercules, CA) in a QuantStudio® 3 real-time PCR system (Applied Biosystems, Foster City, CA). Primers were generated in Primer3 (Rozen and Skaletsky 1999) and targeted sequences ∼100 bp in length (Supplementary Table 6). RT-qPCR primers were designed to match a region of the transcripts not included in dsRNA constructs using Benchling software (Benchling, San Francisco, CA). Transcript abundance for genes of interest was normalized using the control gene, *β tubulin* (Collins *et al*. 2010). Experiments were performed in biological and technical triplicate (*n* = 12 animals per biological replicate). Data were statistically analyzed and visualized using Prism GraphPad Version 7.0 software (GraphPad Software, San Diego, CA).

## Results

### Identification of the planarian G protein subunit repertoire

To better understand G protein-coupled receptor signaling in planarians, we identified 38 G protein subunit homologs (26 Gα subunits, 7 Gβ subunits, and 5 Gγ subunits) in *S. mediterranea* transcriptomes based on the presence of key domains (Brandl *et al*. 2016; Rozanski *et al*. 2019) (Fig. 1b, Supplementary Table 1). This list included all 5 previously identified planarian G protein subunit genes (Cebrià *et al*. 2002; Iglesias *et al*. 2011; Lapan and Reddien 2012). Both numbers and proportions of subunits are consistent with those found in other animals, including humans (Syrovatkina *et al*. 2016), *C. elegans* (Jansen *et al*. 1999), and *Drosophila* (Malpe *et al*. 2020). These results suggest that planarians utilize a typical repertoire of heterotrimeric G protein subunits.

We next classified planarian heterotrimeric subunit homologs into families using phylogenetic analysis. We classified 7 Gαi/o homologs, 4 Gαs homologs, 2 Gαq/11 homologs, 2 Gα12/13 homologs, 3 Gβ1-4 subgroup homologs, and 1 Gβ5 homolog (Fig. 1b; Supplementary File S1; S2). One Gα homolog and 3 Gβ homologs contained all functional domains (Supplementary File S1) but did not cluster with a specific family (Suppplementary File S2). We therefore designated these genes as “Gαx” or “Gβx” (Fig. 1b). Additionally, 10 Gα class homologs retrieved in our search were truncated, preventing accurate classification (Supplementary File S3). We designated these genes *Gα-like* (Fig. 1b). Lastly, due to the divergent nature of Gγ homologs, we were unable to classify them into families, so we designated them as “Gγ-like” (Fig. 1b; Supplementary File S1; S2). Our phylogenetic analysis suggests that the Gα class homolog *gpas* (Cebrià *et al*. 2002; Iglesias *et al*. 2011) was previously misclassified, and the name *Gαi2* more accurately represents this subunit's classification.

After defining the *S. mediterranea* heterotrimeric G protein complement, we next sought to characterize the expression patterns of these genes, to potentially provide insight into tissue-specific roles and possible heterotrimer combinations. We observed broad expression for 9 G protein subunit homologs (Fig. 1c; Supplementary Fig. 1). However, many subunits showed tissue-specific enrichment in the nervous system (Fig. 1d; Supplementary Fig. 1) or the intestine (Fig. 1e). Lastly, we detected no expression pattern for 2 subunits through ISH (Supplementary Fig. 1). In addition to our observations, we determined that 32 of the 38 subunits are expressed within stem cells based on available transcriptomic resources (Labbé *et al*. 2012; Fincher *et al*. 2018; Plass *et al*. 2018; Zeng *et al*. 2018) (Supplementary Table 1). Our results suggest that *S. mediterranea* heterotrimeric G proteins likely function in many different tissue types, including stem cells and a diverse set of neural cell types.

### Elucidation of roles for heterotrimeric G proteins in planarian behavior

We next performed, to our knowledge, the first comprehensive investigation into roles for planarian heterotrimeric G proteins. We completed an RNAi screen by feeding dsRNA and assessing behavioral and regenerative phenotypes (Fig. 2a). We evaluated the penetrance of RNAi for a sampling of 5 genes in these screens using RT-qPCR and observed knockdown efficiency ranging from 92 to 98% (Supplementary Fig. 2a). Specificity of knockdown was examined with the two subunits with the most similar DNA sequence, *Gβ1-4a* and *Gβ1-4b* (58% identity, with stretches of up to 20 identical base pairs). We note that we did observe some degree of cross-reactivity for the dsRNA of these subunits at the level of RT-qPCR but did not see overlap in phenotypes after RNAi (Supplementary Fig. 2b).

Though our ultimate focus was on regeneration, during our screens, we incidentally observed that knockdown of 5 G protein subunit-encoding genes caused behavioral phenotypes. The strongest behavior we documented was reduced movement and paralysis in *Gαs1(RNAi)* animals, which was most clear when animals were placed on their dorsal sides (Supplementary Video S1). All control animals righted themselves after being placed on their dorsal side, taking an average of 27.35 s (Fig. 2b–c). In contrast, 5 of 20 *Gαs1(RNAi)* animals failed to flip onto their ventral side within 5 min. The remaining *Gas1(RNAi)* animals took an average of 168 s to flip (Fig. 2b–c). These results indicate that *Gas1* is required for the righting response and gross movement in planarians. Although we saw reduced movement prior to amputation, the paralysis and flipping phenotypes were enhanced after amputation or long-term RNAi, which may suggest that the movement phenotype results from loss of a slow-turnover cell type (Supplementary Table 2).

Inhibition of any of 4 genes—*Gαs2*, *Gβx2*, *Gβ1-4a*, and *Gαq1*—resulted in decreased gliding movement, which leads to “inching” behavior (Glazer *et al*. 2010). The quickest effects were seen following RNAi of *Gαs2* or *Gβx2*. We first documented the inching after amputation (Fig. 2d), but the phenotype was nearly identical in intact worms (Fig. S3a; Supplementary Table 2; Supplementary Videos 2-3). Movement defects in *Gαs2(RNAi)* and *Gβx2(RNAi)* animals resulted in reduced distance traveled over time (Fig. 2e; Supplementary Fig. 3b). *Gαq1(RNAi)* animals also appeared to move slower than controls in short-term RNAi paradigms, and amputation marginally increased this phenotype (Supplementary Table 2). After long-term RNAi, *Gαq1(RNAi)* animals displayed labored movement (Supplementary Fig. 3c; Supplementary Video 4). *Gβ1-4a(RNAi)* animals alternated between inching and gliding, most perceptibly after amputation or long-term RNAi (Supplementary Fig. 3c; Supplementary Table 2; Supplementary Video 4). An assay documenting negative phototaxis in these animals also demonstrated slow movement to a dark area of a dish, with the strongest effects resulting from perturbation of *Gβx2* (Supplementary Fig. 3d). Finally, we also note that *Gαq1(RNAi)*, *Gβ1-4a(RNAi)*, *Gαs2(RNAi)*, and *Gβx2(RNAi)* animals spent a noticeable amount of time raising and turning their heads, which may be indicative of additional sensory or movement dysfunction.

Locomotion of *Gαs2(RNAi)* and *Gβx2(RNAi)* animals was indistinguishable, which led us to hypothesize that *Gαs2* and *Gβx2* might be operating in the same cells. We noted that *Gαs2* is expressed in a head margin pattern consistent with putative peripheral sensory neurons (Ross *et al*. 2018) (Fig. 1d, Fig. 2f). *Gβx2* is expressed in a similar pattern but also in cells of the intestine (Fig. 1e, Fig. 2f). We further validated the coexpression of *Gαs2* and *Gβx2* in cells at the tip of the head through FISH (Fig. 2g). The colocalization of *Gαs2* and *Gβx2* transcripts supports the hypothesis that they could potentially work in the same cells.

Our goal in focusing on heterotrimeric G proteins was to uncover roles for GPCRs. As proof-of-principle, we next sought to identify the GPCR that works with *Gαs2* and *Gβx2*. We identified and screened 8 GPCR-encoding genes enriched in the same cell clusters as *Gαs2* or *Gβx2* in available single cell sequencing datasets (Fincher *et al*. 2018; Plass *et al*. 2018) (Fig. 2h; Supplementary Table 3). Using this method, we identified a putative serotonin receptor, *gcr052* [the homolog of *DtSER-1* (Zamanian *et al*. 2012), *S7.1R* (Chan *et al.* 2015, 2016), and *Smed-ser85* (Zamanian 2011) in planarian literature], for which knockdown caused inching indistinguishable from that displayed by *Gαs2(RNAi)* and *Gβx2(RNAi)* animals (Fig. 2i–j; Supplementary Videos 2-3; Supplementary Fig. 3a–b).

*gcr052* is expressed broadly throughout the CNS (Fig. 2k). Using FISH, we detected coexpression of *gcr052* with *Gαs2* and *Gβx2* in many cells, including clusters at the anterior (Fig. 2l). While targeting *Gαs2*, *Gβx2*, and/or *gcr052* in combination did not noticeably exacerbate the phenotype (Supplementary Fig. 3e–f; Supplementary Video S5), some GPCR research indicates that loss of one component can prevent the assembly of the receptor/trimer complex (Smrcka 2008; Dupré *et al*. 2009). We thus hypothesize that Gαs2 and Gβx2 act downstream of the GCR052 receptor to support gliding motion.

In summary, our results show that 5 planarian heterotrimeric G proteins are essential for normal animal movement. Additionally, our identification of GCR052 provides proof-of-principle that the heterotrimeric G proteins characterized in this work can accelerate planarian GPCR research.

### Planarian heterotrimeric G proteins function in regeneration

Over the course of our functional analysis, we knocked down each G protein subunit and assessed the degree of brain regeneration after amputation (Fig. 3a–e; Supplementary Table 4), because brain size is a highly robust way of detecting regeneration defects (Roberts-Galbraith *et al*. 2016). After screening 37 of the 38 predicted subunit genes, we found 7 genes for which RNAi caused significant reduction in brain regeneration (Fig. 3b–e). Of these candidates, RNAi targeting *Gαs1*, *Gαs2*, *Gαo2*, *Gαq2*, or *Gα-like6* produced modest effects (Fig. 3b–e). RNAi targeting *Gαq1* or *Gβ1-4a* caused a strong reduction of brain regeneration (Fig. 3b–e). Of these genes, knockdown of 3 candidate subunits, *Gαs1*, *Gαq1*, or *Gβ1-4a*, also caused reduction in tail regeneration (Supplementary Fig. 4a–c). These results show that multiple Gα class and one Gβ class subunit play roles in planarian regeneration.

Interestingly, we detected no significant regeneration phenotypes after RNAi targeting individual Gγ subunit genes (Fig. 3e). To account for potential functional redundancy among Gγ subunits, we observed brain regeneration after combinatorial RNAi targeting all identified Gγ class subunit genes (Supplementary Fig. 4d). Indeed, targeting these genes concurrently produced a significant ∼46% reduction in regenerated brain size (Supplementary Fig. 4d). Furthermore, RNAi of *Gγ-like1*, *Gγ-like4*, and *Gγ-like5* together caused a severe reduction in brain regeneration (Fig. 3f–g). These results indicate that Gγ subunits are likely functionally redundant and have cooperative roles in regeneration.

Due to the strong roles for *Gαq1* and *Gβ1-4a* in regeneration, we sought to further identify the cell types that express *Gαq1* and *Gβ1-4a* and determine whether these genes are expressed in overlapping cells. Based on the colorimetric ISH expression patterns, the transcripts of *Gαq1* and *Gβ1-4a* appear to both be particularly enriched in the central nervous system and eyespots (Fig. 1d). Additionally, based on published sequencing datasets, these genes are also detected in muscle and at low levels in stem cells (Supplementary Table 1). We confirmed expression of *Gαq1* and *Gβ1-4a* in the brain branches and eyespots through FISH (Fig. 3h). Additionally, due to the highly enriched expression in the eyespots, we took a closer look at these cells and saw that *Gαq1* and *Gβ1-4a* transcripts indeed colocalize (Fig. 3h). Although we require biochemical analyses to prove functional pairing, these results show that *Gαq1* and *Gβ1-4a* are expressed in overlapping cell populations.

Additionally, we considered that *Gαq1* or *Gβ1-4a* could impact regeneration by affecting the timing of tissue regrowth. To determine whether the phenotypes we saw were due to delays in regeneration, we observed brain regeneration at 14 days postamputation (dpa) in knockdown animals. *Gαq1(RNAi)* and *Gβ1-4a(RNAi)* animals showed partial recovery of regenerated brain size with additional time (Supplementary Fig. 4e). However, we note that the distribution of brain regeneration is not the same in *Gαq1(RNAi)* and *Gβ1-4a(RNAi)* animals. A small proportion of *Gαq1(RNAi)* animals failed to initiate any regenerative response, and while the rest of the animals regenerated the expected brain size, brain morphology appeared more collapsed toward the midline relative to control brains (Supplementary Fig. 4e). In contrast, all *Gβ1-4a(RNAi)* animals regenerated a reduced, but otherwise normal, bilobed brain structure (Supplementary Fig. 4e). Our results at 14 dpa support the notion that *Gβ1-4a* promotes the speed of brain regeneration, whereas *Gαq1* shows a more complex role in brain regeneration including initiation of regenerative response and proper morphology of the mature CNS.

Through these studies, we find that multiple heterotrimeric G proteins promote regeneration, with *Gαq1* and *Gβ1-4a* playing especially critical roles. Further, although we saw overlap in roles for regeneration and behavior after perturbation of some genes (*Gαs1*, *Gαs2*, *Gαq1*, and *Gβ1-4a*), some genes specifically impact regeneration (*Gαo2*, *Gαq2*, *and Gα-like6*) or behavior (*Gβx2)* (Figs. 2 and 3, and Supplementary Table 2).

### *Gβ1-4a* promotes mitotic response after amputation and long-term survival

Our next goal was to understand why *Gαq1* and *Gβ1-4a* are critical for regeneration. We first considered whether *Gαq1* and *Gβ1-4a* affect initial response to wounding. We examined the expression of *Gαq1* and *Gβ1-4a* after injury. Indeed, we found that *Gαq1* and *Gβ1-4a* are upregulated at the amputation site at both 6 h postamputation (hpa) and 3 dpa (Supplementary Fig. 5a). Planarians initiate a molecular wound response program during this time that includes upregulation of genes like *follistatin*, *jun-1*, *inhibin*, and *wnt1* (Wenemoser *et al*. 2012; Wurtzel *et al*. 2015). We determined that *Gαq1(RNAi)* and *Gβ1-4a(RNAi)* animals expressed wound-induced genes normally at 6 hpa (Supplementary Fig. 5b–c). The only significant difference we observed was a mild increase in *follistatin* transcripts in *Gαq1(RNAi)* animals, detected through RT-qPCR (Supplementary Fig. 5c). These results suggest that while *Gαq1* and *Gβ1-4a* are upregulated during regeneration, they are dispensable for early injury response.

**Fig. 4.**
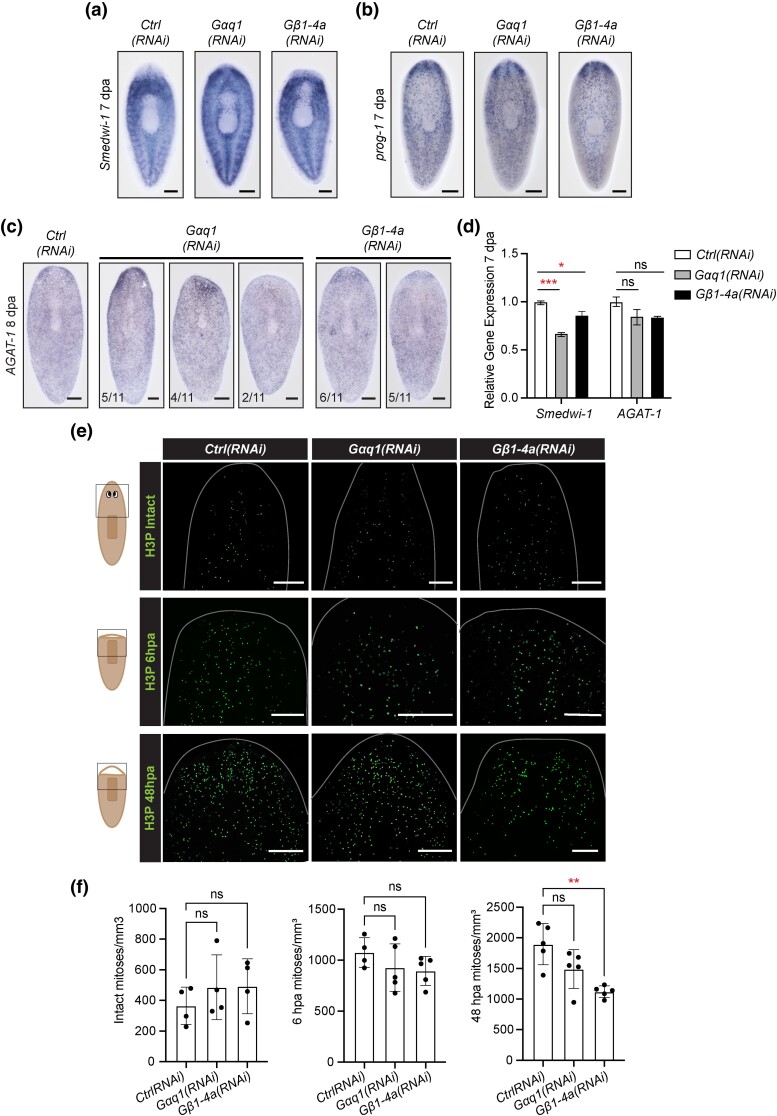
*Gαq1* and *Gβ1-4a* are not required for stem cell maintenance. Representative images of (a) *Smedwi-1*, (b) *prog-1*, and (c) *AGAT-1* ISH in regenerating animals after RNAi targeting *Gαq1* and *Gβ1-4a*. d) Relative transcript abundance of stem cell markers, measured by RT-qPCR. Differences were analyzed with one-way ANOVA with multiple comparisons. Error bars represent standard error. * = *P* value ≤ 0.05. *** = *P* value ≤ 0.0005. e) Representative images of proliferative cell detection (anti-H3P) at the anterior region of intact, 6-h regenerating and 48-h regenerating RNAi animals. f) Results from quantification of H3P^+^ cells detected in the anterior region of the animals at each timepoint, displayed as mean and standard deviation. Differences were analyzed with Brown–Forsythe and Welch ANOVA with multiple comparisons. ** = *P* value ≤ 0.01. Scale bars = 200 μm.

Next, we investigated whether the regeneration defects observed after *Gαq1(RNAi)* or *Gβ1-4a(RNAi)* result from perturbed stem cell maintenance or differentiation. We looked at expression of a stem cell marker [*Smedwi-1* (Reddien *et al*. 2005)] and epidermal progenitor markers [*prog-1* and *AGAT-1* (Eisenhoffer *et al*. 2008; Tu *et al*. 2015)] after head regeneration. We did not see depletion of stem cell or progenitor markers through ISH (Fig. 4a–c). However, transcript abundance of *Smedwi-1* showed modest or mild reduction through RT-qPCR after RNAi of *Gαq1* or *Gβ1-4a*, respectively (Fig. 4d).

We also examined mitotic activity of stem cells in *Gαq1(RNAi)* and *Gβ1-4a(RNAi)* animals. Planarian stem cells divide at a regular rate in intact animals, and after amputation two primary waves of mitosis occur: one at ∼6 hpa that is body-wide and one at ∼48 hpa that is localized to the amputation site (Baguna *et al*. 1989; Wenemoser and Reddien 2010). To investigate the rates of stem cell division in *Gαq1(RNAi)* and *Gβ1-4a(RNAi)* animals, we performed an antibody stain for a histone modification associated with mitosis (phospho-histone-H3-Ser10) (Hendzel *et al*. 1997; Newmark and Sánchez Alvarado 2000). We detected a significant decrease in proliferative cells in *Gβ1-4a(RNAi)* animals at 48 hpa, but otherwise the mitotic activity in *Gαq1(RNAi)* and *Gβ1-4a(RNAi)* animals appeared comparable to controls (Fig. 4e–f). We also found that *Gβ1-4a* is not strongly coexpressed with *Smedwi-1* in intact or regenerating animals, but we did see *Gβ1-4a^+^* and *Smedwi-1^+^* cells near one another in regenerating tissue (Supplementary Fig. 6a–b), suggesting that any effect of Gβ1-4a signaling on stem cells might be noncell autonomous. We conclude that *Gαq1* and *Gβ1-4a* may play subtle roles in stem cell maintenance, differentiation, or division, but that these defects are likely insufficient to explain the severe regenerative phenotypes seen in RNAi animals.

Finally, we asked whether the roles of *Gαq1* and *Gβ1-4a* were exclusive to regeneration or whether either gene also functioned during homeostasis. We performed longer term RNAi and measured animal growth and survival over time (Supplementary Fig. 6c). *Gβ1-4a(RNAi)* animals ceased growth after day 21 and we halted the growth measurements of *Gαq1(RNAi)* animals at that time point because they began to fission (Supplementary Fig. 6d). Long-term RNAi targeting *Gβ1-4a* was lethal, with animals showing head lysis and dying near day 40 (Supplementary Fig. 6e–f). We also noted postural changes without change in viability in *Gαq1(RNAi)* animals, suggesting that *Gαq1* promotes head regeneration but is not required for head maintenance (Supplementary Fig. 6e–f). Intriguingly, *Smedwi-1^+^* cells remained abundant at later timepoints of RNAi (Supplementary Fig. 6g–h), suggesting that the stem cells are maintained even as *Gβ1-4a(RNAi)* animals begin to lyse.

To summarize, our data indicate that *Gαq1* is essential for regeneration but not strictly required for wound response induction, mitosis, or stem cell maintenance. Long-term inhibition of *Gβ1-4a* is lethal, but other than modestly promoting the late wave of mitotic response after amputation, we did not detect strong impacts of *Gβ1-4a* perturbation on stem cell regulation. Importantly, several results indicate key differences in function for *Gαq1* and *Gβ1-4a,* despite the two genes having similarly important roles in regeneration. Because the impacts we saw for both subunits in stem cell biology were mild, we sought to examine influences of *Gαq1* and *Gβ1-4a* on other physiological processes that contribute to regeneration.

### *Gαq1* and *Gβ1-4a* support the late phase of anterior–posterior polarity reestablishment

**Fig. 5.**
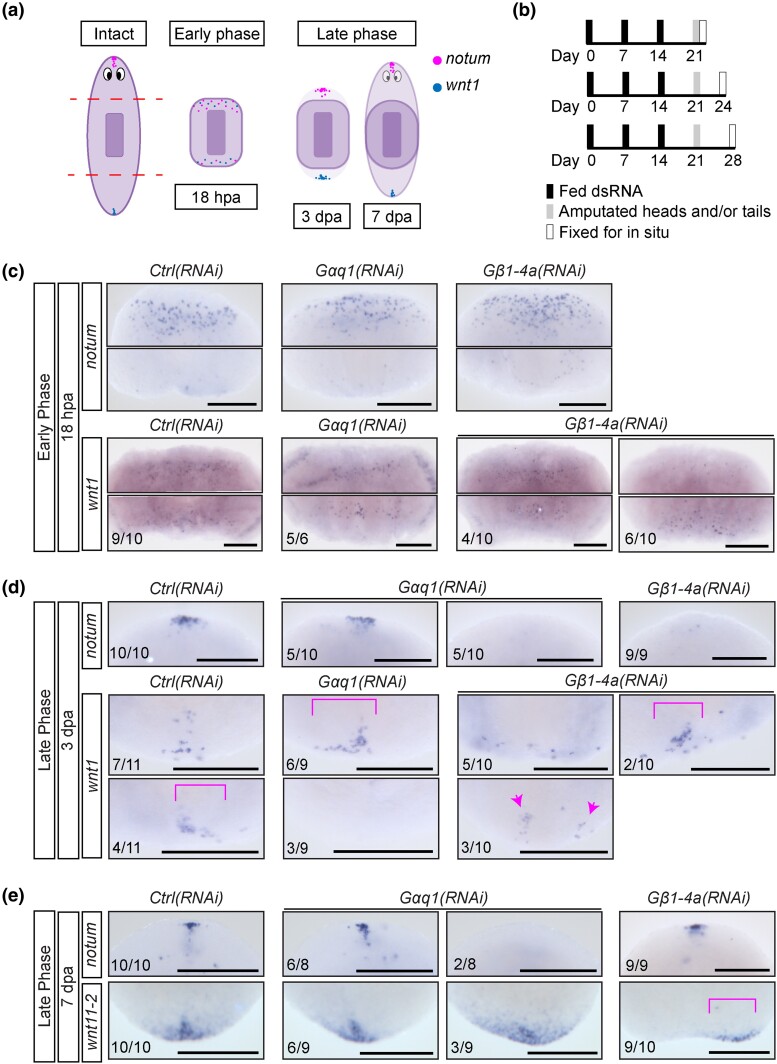
*Gαq1* and *Gβ1-4a* support the late phase of anterior and posterior pole regeneration. a) Graphic summary depicting phases of polarity reestablishment after head and tail amputation, as summarized in (Owlarn and Bartscherer 2016). b) RNAi paradigms for 18 hpa (top), 3 dpa (middle), and 7 dpa (bottom). The following images are zoomed to focus on the regenerating head or tail blastemas for each stage. Representative images of anterior *notum*, and posterior *wnt1* or *wnt11-2* expression at (c) 18 hpa, (d) 3 dpa, and (e) 7 dpa of heads (pointing upward) and/or tails (pointing downward). Brackets denote nonmedial expression domains. Arrowheads indicate multiple expression domains. Scale bars = 200 μm.

Early in planarian regeneration, tissues reorganize to pattern body axes using conserved developmental signaling pathways [e.g. Wnt/β-catenin (Gurley *et al*. 2008; Iglesias *et al*. 2008; Petersen and Reddien 2008)]. We next considered whether regeneration failures after *Gαq1(RNAi)* or *Gβ1-4a(RNAi)* occur due to abnormal body polarity. During the early phase of polarity reestablishment, the remaining tissue determines which end of the animal is anterior and which is posterior (Fig. 5a) [reviewed in (Owlarn and Bartscherer 2016)]. To determine whether *Gαq1(RNAi)* and *Gβ1-4a(RNAi)* animals correctly complete the initial anterior–posterior decision, we examined *notum* and *wnt1* expression 18 hpa (Fig. 5b). *notum* expression resumed normally at the anterior in *Gαq1(RNAi)* and *Gβ1-4a(RNAi)* animals (Fig. 5c). *Gαq1(RNAi)* animals also expressed bipolar *wnt1*, but over half of *Gβ1-4a(RNAi)* animals displayed posterior-enriched expression of *wnt1* (Fig. 5c). These results suggest that *Gαq1* is not involved in early polarity decisions, but *Gβ1-4a* might affect anterior wound-induced *wnt1* expression.

After re-initiation of axial polarity, anterior and posterior poles form at the distal ends of the planarian body (Fig. 5a). To determine whether pole formation was disrupted in *Gαq1(RNAi)* and *Gβ1-4a(RNAi)* animals, we analyzed *notum* and *wnt1* expression at 3 dpa (Fig. 5b). 50% of *Gαq1(RNAi)* animals and all *Gβ1-4a(RNAi)* animals lacked anterior *notum* expression (Fig. 5d). Additionally, *Gαq1(RNAi)* animals displayed an asymmetric *wnt1* pattern or absent *wnt1* in the posterior domain, and *Gβ1-4a(RNAi)* animals regenerated with a broadened and/or asymmetrical domain of *wnt1* expression (Fig. 5d). Our results indicate that *Gαq1* and *Gβ1-4a* impact pole formation at both anterior and posterior ends of the animal.

During the late phase of polarity reestablishment, anterior and posterior poles further coalesce and mature (Fig. 5a). To investigate whether *Gαq1* and *Gβ1-4a* support the maturation of the key polarity domains, we examined expression of *notum* and another posterior marker, *wnt11-2* (Gurley *et al*. 2008), in knockdown animals at 7 dpa (Fig. 5b). We observed a lack of *notum* staining at the anterior pole in ∼25% of *Gαq1(RNAi)* animals (Fig. 5e), and we confirmed this pattern by using a second pole marker, *sFRP-1* (Gurley *et al*. 2008; Petersen and Reddien 2008) (Supplementary Fig. 7a). Posterior pole maturation was also disrupted in ∼33% of *Gαq1(RNAi)* animals, which displayed broader and more diffuse *wnt11-2* expression (Fig. 5e). All *Gβ1-4a(RNAi)* animals recovered anterior *notum* expression by 7 dpa, although the domains appeared less consolidated than in control animals, which could signify slower maturation (Fig. 5e, Supplementary Fig. 7a). The formation of an anterior pole domain at a slower pace is consistent with our previous results suggesting that *Gβ1-4a* largely affects the speed of head regeneration rather than ultimate success (Supplementary Fig. 4e). Strikingly, most *Gβ1-4a(RNAi)* animals expressed posterior *wnt11-2* asymmetrically, with staining on either side of the animal's midline (Fig. 5e). Notched tails were also commonly seen after *Gβ1-4a(RNAi)* (Supplementary Fig. 4c), though our data did not support the presence of a secondary anterior domain, as has been seen after other RNAi treatments (Supplementary Fig. 7b–c) (Cloutier *et al*. 2021).

Taken together, we conclude that *Gβ1-4a* supports the speed of anterior pole reestablishment and promotes proper midline placement of the posterior pole. Our data also support a role for *Gαq1* in promoting robust anterior pole formation, though this phenotype was limited to a minority of animals. Interestingly, while both *Gαq1* and *Gβ1-4a* function during regeneration and influence anterior–posterior polarity, the precise phenotypes seen after RNAi of *Gαq1* and *Gβ1-4a* are distinct.

### *Gαq1* promotes head regeneration through production and activity of *follistatin*  ^+^ anterior pole cells

The anterior pole is established and maintained through two mutually dependent signaling proteins, Notum and Follistatin (Petersen and Reddien 2011; Gaviño *et al*. 2013; Roberts-Galbraith and Newmark 2013). *notum* and *follistatin* encode key extracellular inhibitors of posterior-promoting Wnt and Activin pathways, respectively (Nakamura *et al*. 1990; Kakugawa *et al*. 2015). We noted several similarities between the phenotypes caused by *follistatin(RNAi)* and those caused by *Gαq1(RNAi)* or *Gβ1-4a(RNAi).* Similarities include strong impacts on head and brain regeneration; reduced or delayed *notum* expression in the regenerating head; unaffected expression of early wound response genes; and subtle impacts on stem cells (Gaviño *et al*. 2013; Roberts-Galbraith and Newmark 2013; Tewari *et al*. 2018).

**Fig. 6.**
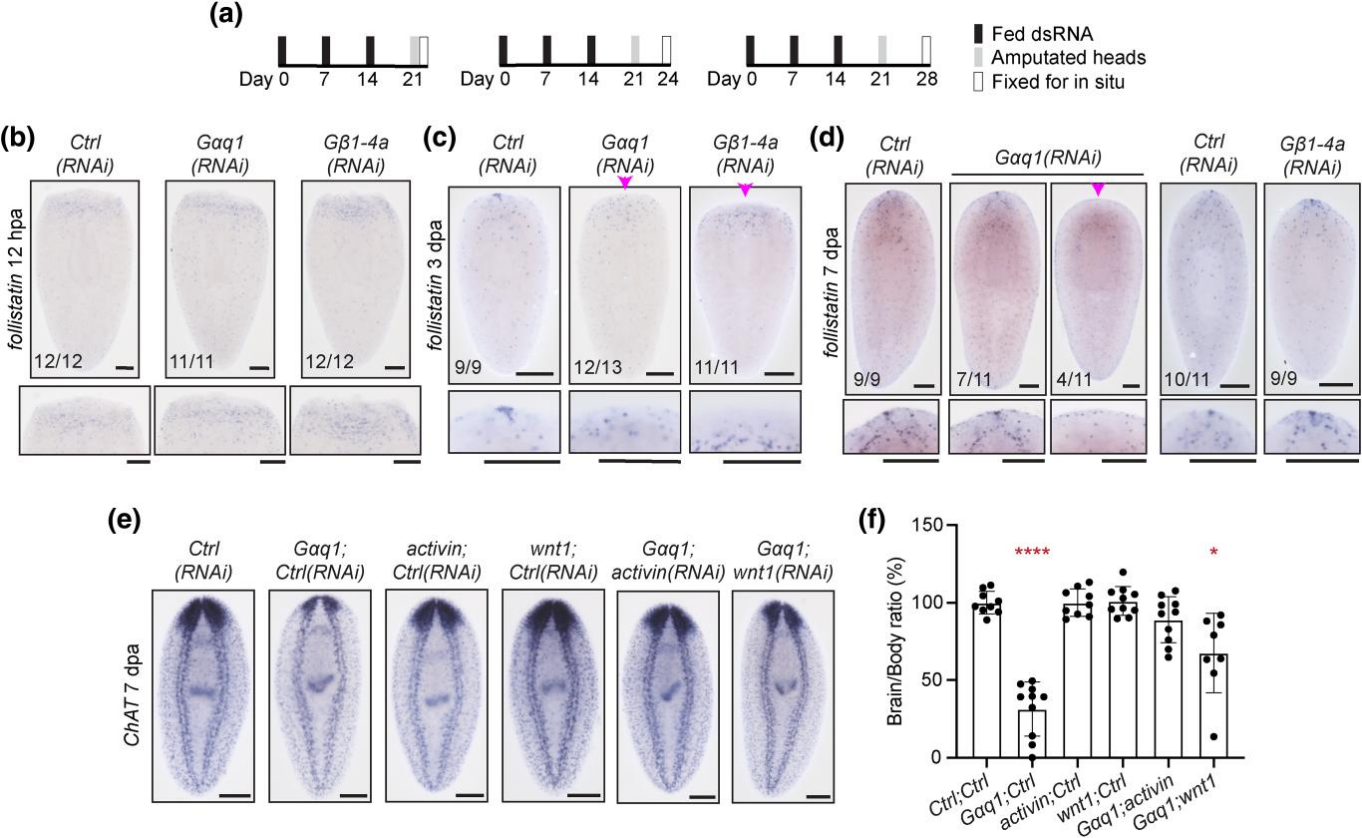
*Gαq1* supports head regeneration through production of *follistatin* ^+^ anterior pole cells. a) RNAi paradigms used for data presented at 12 hpa (left), 3 dpa (middle), and 7 dpa (right). Representative images of *follistatin* expression at (b) 12 hpa, (c) 3 dpa, and (d) 7 dpa in *Gαq1(RNAi)* and *Gβ1-4a(RNAi)* animals. Arrowheads indicate absent anterior pole expression domain. Insets show close-up images of the animals above. e) Representative images showing *ChAT* expression from rescue experiments 7 dpa. f) Bar graph showing results from quantification of brain/body ratios in rescue experiments, displayed as mean and standard deviation. Differences were analyzed using Brown–Forsythe and Welch ANOVA with multiple comparisons. * = *P* value ≤ 0.05 and **** = *P* value ≤ 0.0001. Scale bars = 200 μm.

Based on phenotypic similarities, we sought to determine whether RNAi of *Gαq1* or *Gβ1-4a* impacts *follistatin* expression during regeneration (Fig. 6a). We detected no change in *follistatin* expression at 12 hpa after perturbation of *Gαq1* or *Gβ1-4a* (Fig. 6b). We similarly saw equivalent or higher *follistatin* transcripts 6 hpa through RT-qPCR (Supplementary Fig. 5c). These results indicate that regeneration failure in *Gαq1(RNAi)* and *Gβ1-4a(RNAi)* animals is not correlated with a reduction of wound-induced *follistatin* expression.

To determine whether *Gαq1* and *Gβ1-4a* support *follistatin* expression in the anterior pole, we examined *follistatin* expression during pole formation (Fig. 6a). At 3 dpa, most *Gαq1(RNAi)* and all *Gβ1-4a(RNAi)* animals had absent *follistatin* expression at the anterior pole (Fig. 6c). At 7 dpa, ∼36% of *Gαq1(RNAi)* animals still lacked *follistatin^+^* anterior pole cells (Fig. 6d). However, all *Gβ1-4a(RNAi)* animals established *follistatin^+^* pole cells by 7 dpa (Fig. 6d), reflecting a similar delay in anterior pole formation seen with other markers (Fig. 5 and Supplementary Fig. 7).

Both *notum* and *follistatin* expression in anterior pole cell progenitors relies on a key transcription factor, encoded by *foxD* (Roberts-Galbraith and Newmark 2013; Scimone *et al*. 2014; Vogg *et al*. 2014). To investigate whether Gαq1 could modulate *follistatin* through FoxD, we examined *foxD* expression following *Gαq1* knockdown. Indeed, anterior *foxD* expression was absent in 50% of *Gαq1(RNAi)* animals at 3 dpa and ∼36% of animals at 7 dpa (Supplementary Fig. 7d–e). We confirmed this significant reduction of *foxD* expression through RT-qPCR (Supplementary Fig. 7f). *Gβ1-4a(RNAi)* animals displayed absent *foxD* anterior pole expression 3 dpa and most animals resumed *foxD* expression by 7 dpa (Supplementary Fig. 7d–e). Thus, our data suggest that impacts on *follistatin* expression could be mediated by *foxD.* Alternatively, the lack of these anterior pole markers could result from a failure to produce and/or specify anterior pole progenitors, resulting in fewer pole cells.

Previous work characterizing the Follistatin/Activin and Notum/Wnt pathways determined that reduction of the antagonistic posterior-promoting ligands rescued head regeneration (Petersen and Reddien 2011; Gaviño *et al*. 2013; Roberts-Galbraith and Newmark 2013). The similarities between *Gαq1(RNAi)* and *follistatin(RNAi)* phenotypes and the impact of *Gαq1(RNAi)* on *follistatin* expression led us to hypothesize that *Gαq1* functions to promote Follistatin signaling from the pole. To test this hypothesis, we performed RNAi targeting *Gαq1* with *activin(RNAi)*, *wnt1(RNAi)*, or *bmp4(RNAi)* (a TGF-β ligand that impacts dorsoventral polarity) (Reddien *et al*. 2007; Gaviño *et al*. 2013; Roberts-Galbraith and Newmark 2013; Tewari *et al*. 2018). *activin(RNAi)* significantly rescued *Gαq1(RNAi)*-induced brain regeneration defects (Fig. 6e–f; Supplementary Fig. 8a–e). *wnt1(RNAi)* also partially rescued *Gaq1(RNAi)* (Fig. 6e–f; Supplementary Fig. 8a–e). As expected, *bmp4(RNAi)* failed to rescue regeneration in *Gαq1(RNAi)* animals (Supplementary Fig. 8a–b). Our results were also confirmed in a second experiment that showed equally strong *Gαq1* knockdown efficiency in double RNAi conditions (Supplementary Fig. 8c–e). Incidentally, though we primarily focused on a functional connection between Gαq1 and Follistatin, we also found that *activin* inhibition modestly restored brain regeneration in *Gβ1-4a(RNAi)* animals (Supplementary Fig. 8f–g). We conclude that Gαq1 function specifically supports Follistatin signaling from the anterior pole during head regeneration.

## Discussion

**Fig. 7.**
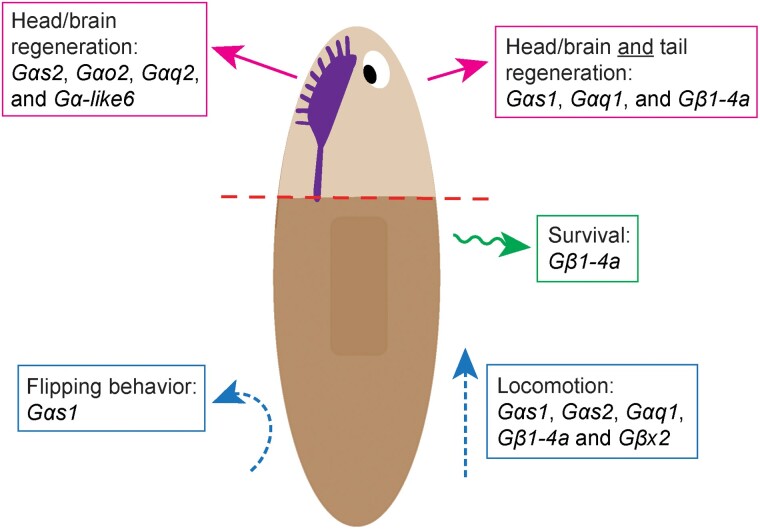
Planarian heterotrimeric G proteins play diverse roles in regeneration, physiology, and behavior. Graphical summary of roles described in this work for heterotrimeric G proteins in *S. mediterranea*.

The vast number of GPCRs hinders progress in understanding the function of this fascinating receptor family in planarian regeneration and stem cell biology. In this work, we take a step toward investigating planarian GPCR signaling by identifying and functionally characterizing the heterotrimeric G protein subunit complement in behavior and regeneration. We characterized 38 heterotrimeric G protein homologs, of which 23 were conserved enough to categorize. Through our functional screens, we identified 5 subunit genes required for proper planarian movement (Fig. 7). Using these data as a starting point and relying on single cell sequencing data, we identified a putative serotonin receptor (GCR052) that could function with Gαs2 and Gβx2 in movement. Through our brain regeneration screen, we identified 7 genes with roles in regeneration, with *Gαq1* and *Gβ1-4a* having especially significant effects (Fig. 7). We determined that *Gαq1* and *Gβ1-4a* promote successful regeneration and establishment speed of the anterior pole, respectively. Our findings indicate new pathways active in planarian regeneration and behavior and support the hypothesis that GPCR signaling is likely to be involved in key molecular events that drive and coordinate planarian regeneration.

Due to the functionally overlapping, but nonidentical effects of *Gαq1* or *Gβ1-4a*, we reason that these subunits could be activated downstream of a common GPCR but stimulate different downstream pathways to support tissue regeneration (Tang and Gilman 1991; Inglese *et al*. 1995; Brock *et al*. 2003). This model is supported by coexpression of *Gαq1* and *Gβ1-4a* through FISH and in single cell sequencing data (Fincher *et al*. 2018) (Fig. 3h; Supplementary Table 1 and 3). Additionally, we show through RT-qPCR that while targeting *Gβ1-4a* does not impact expression of *Gαq1*, expression of *Gβ1-4a* is significantly reduced in *Gαq1(RNAi)* animals (Supplementary Fig. 2c). We also demonstrated phenotypes for *Gβ1-4a(RNAi)* but not *Gβ1-4b(RNAi)*, despite some cross-targeting of dsRNA (Supplementary Fig. 2). This indicates that the relationships between G protein subunits could involve additional redundancy or regulatory elements.

### *Gαq1* provides a putative connection between planarian GPCR signaling and defined polarity axes

The phenotypic similarities between *Gαq1(RNAi)* and *follistatin(RNAi)* animals and the ability to rescue phenotypes via *activin* double knockdown indicate that the Gαq1 protein likely cooperates with Follistatin during regeneration. Our results suggest that Gαq1 could function upstream to promote *follistatin* expression at the anterior pole. How Gαq1 promotes *follistatin* expression and whether this results from a failure to specify early anterior pole progenitors or a failure to turn on key gene networks in differentiating anterior pole cells remain to be determined. Alternatively, Activin signals belong to the transforming growth factor-β (TGF-β) family, and recent work describes the potential for GPCRs to modulate TGF-β pathways through transactivation (Burch *et al*. 2012; Hinck *et al*. 2016; Schafer and Blaxall 2017). Therefore, Gαq1 could potentially influence the Activin/Follistatin axis through a noncanonical mechanism. Further exploring relationships between *Gαq1* and pathway components will help define the nature of the Gαq1/Follistatin cooperation.

Additionally, because *Gαq1(RNAi)* animals displayed functional wound-induced *follistatin* expression, our results also support the notion that *follistatin* expression from the anterior pole is specifically needed to drive successful head regeneration (Gaviño *et al*. 2013; Roberts-Galbraith and Newmark 2013; Tewari *et al*. 2018). Therefore, results from future work with *Gαq1* could inform the nature of anterior identity establishment. Potential roles for *Gαq1* (and GPCRs) in modulating the Activin pathway and promoting polarity reestablishment will require further investigation.

### The relationship between *Gαs2*, *Gβx2*, and *gcr052* suggests complexity of serotonin's role in planarian locomotion

In addition to characterizing planarian heterotrimeric G proteins with roles in regeneration, this work also contributes to knowledge of mechanisms governing planarian movement. The current model for planarian gliding is that serotonergic neurons directly innervate ventral epidermal cells and coordinate the beating of motile cilia (Currie and Pearson 2013; März *et al*. 2013). Furthermore, experiments with mianserin, a pharmacological inhibitor of serotonin receptors, also implicated GPCRs in cilia coordination in *S. mediterranea* (Kuang *et al*. 2002; Currie and Pearson 2013). In this work, we identify 2 G protein subunits (*Gαs2* and *Gβx2*) that similarly affect locomotion in *S. mediterranea* (Fig. 2). We further identified *gcr052* (Saberi *et al*. 2016), which encodes a putative serotonin GPCR, as a potential specific mediator of gliding motion. Homologs of the receptor *gcr052* have well documented roles in movement among planarian species, with coupling validation to Gαs protein family subunits [receptor referred to in literature as *DtSER-1* (Zamanian *et al*. 2012), *S7.1R* (Chan *et al.* 2015, 2016), and *Smed-ser85* (Zamanian 2011)]. We identified the receptor through our study of G proteins, displaying the usefulness of our pipeline method.

Further supporting the notion that Gαs2 and Gβx2 operate together and downstream of GCR052, we identified cells that are enriched with *Gαs2/Gβx2*, *Gαs2/gcr052*, and *Gβx2/gcr052* through FISH (Fig. 2). These cells are patterned similarly to cells of the *soxB1-2^+^* dorsal ciliated stripes of sensory neurons in the peripheral nervous system (Ross *et al*. 2018). While these cells were the most identifiable localization of all 3 transcripts, we note that additional cell types also appeared enriched for one or more of these genes. For example, we also observed high levels of *gcr052* in putative epidermal cells at the periphery of the animal, potentially supporting the model that serotonin directly influences ciliary coordination on the epidermal cells via this receptor (Fig. 2l). However, *Gαs2* and *Gβx2* transcripts were not highly enriched in these cells, suggesting that serotonin signaling to other cells, such as the putative neurons described here, may also be important for planarian locomotion. Future work further characterizing the specific cells in which *Gαs2*, *Gβx2*, and *gcr052* operate in vivo, along with detailed documentation of how these genes affect planarian motile cilia, could elucidate the mechanisms regulating neural control of cilia-based gliding.

Furthermore, additional assays may reveal new roles of heterotrimeric G proteins in behavior and sensation. G proteins act in diverse biological processes, such as sensation, in other animals (Jones and Reed 1989; Yarfitz and Hurley 1994; Wong *et al*. 1996), and 8 planarian G protein subunits show expression enrichment in sensory structures called the brain branches (Agata *et al*. 1998; Okamoto *et al*. 2005), further supporting this notion (Fig. 1; Supplementary Fig. 9). Using the G protein group as a primary screening strategy may be a beneficial starting point for future study of GPCRs in planarian sensory neurobiology or other aspects of planarian physiology.

### Planarian heterotrimeric G proteins can suggest candidate receptors for future planarian GPCR research

Because GPCRs represent one of the largest receptor families in many organisms, including humans (Fredriksson *et al*. 2003) and planarians (Zamanian *et al*. 2011; Saberi *et al*. 2016), approaches to accelerate identification of relevant GPCRs for a given process can prove to be valuable. Our investigation into planarian heterotrimeric G protein subunits produced functionally distinct and measurable phenotypes, supporting the idea that planarian heterotrimeric G protein subunits could provide a practical first step for identifying and studying roles of GPCRs.

To develop a G protein subunit-driven candidate approach, we formulated a pipeline that identifies candidate GPCR genes using phenotypes from our work along with published single cell sequencing datasets (Fig. 2h; Supplementary Table 3) (Fincher *et al*. 2018; Plass *et al*. 2018; Swapna *et al*. 2018). Our work with *Gβx2* and *gcr052* demonstrates the utility of characterizing heterotrimeric G proteins as a first step in identifying relevant GPCRs and understanding the cellular mechanism (Wise *et al*. 2004; Oh *et al*. 2006; Civelli *et al*. 2013; Ngo *et al*. 2016). In the future, we plan to apply this approach to identify candidate GPCRs that work with heterotrimeric G proteins to promote polarity establishment and successful regeneration. Identification of novel signaling pathways with key roles in regeneration will help us understand how information about injury is converted into cellular responses to coordinate and drive planarian regeneration.

## Supplementary Material

iyad019_Supplementary_Data

## Data Availability

Planarians and plasmids are available upon request. The authors affirm the inclusion of all relevant data and all data required to confirm conclusions of the work are present in the figures, article, tables, and supplementary material associated with this manuscript. Supplementary material is available at the *Genetics* website. In situ hybridization data will also be shared at the Planarian Anatomy Gene Expression (PAGE) database. Supplemental material available at GENETICS online.
