# Supplementary material for: Heterotrimeric G proteins regulate planarian regeneration and behavior
Source: Genetics. 2023 Feb 10;223(4):iyad019. doi: 10.1093/genetics/iyad019 (PMC10078920; doi:10.1093/genetics/iyad019)
Supplement: iyad019_Supplementary_Data [file iyad019_supplementary_data.zip › File_S1_GENETICS-2022-305416.pdf]

```
                                                          α1                    β1
RatGnai1[Ai]
                1                  10      ▲  ▲▲  20▲▲  ▲▲   ▲▲30
RatGnai1[Ai]      ........MGCTL...............SAEDKA.AVERSKMIDRNLREDGEKAAREVKL
22522             ........MNCNFKISSHLN......TINQISTQKSVLRSREIDQQLMQDQELRKKEIQI
2931              .......MLPCIPK.RSRF........IGDVDIKHQKAKSREIDKLMKREKRKFLTRHSI
8947              .......MFTCFKNSKKKF........YKEIDLKSQKSFSKLIDKSLKIERRKNLMKQHI
33116             ............................................................
9665              ......MPAICGGKSA.................STEDKSNKEIDKKLKKDKKTFLMTHRI
6167              ......MALSCAAF............KTETEEQKNRKESNKRIEKCIEKEKRSFKSTHRL
5082              ........MACCN...............SEEFKE.QSRINKEIEKQLKRDKKDARRELKL
3755              ........MGCCL...............SELDKE.QKRINKEIEKQLKKDKKESRRELKL
1773              ........MGCPL...............GKPDEIEQNEISHKIDKELKLEAKHSYRIVKL
13658             ............................................................
9715              ........MGCAM...............SLDERI.ALNKSKDIDKKLKEDGLQLQRDIKL
1656              ........MGCTM...............SKDERD.ALDKSRNIDKKLKEDGMQAAKDVKL
12112             ........MGCAG...............SVPDKD.QQEKNKEIEQQLRQDAEKAMKEVKL
19937             ........MGCIN...............STKNVI.QEEAHKEIEKQLRIDQEKAMREIKL
47050complete     ........MGCIN...............SSHDEP.QQELNKNIEKQLRIDAEKAMKEVKL
4493              .......MNTCISKVETDEQREKR......VLLEENRRKNNQIEKELALYKNKKLSCLKL
HumanGNAS[As]     ........MGCLGNSK.........TEDQRNEEKAQREANKKIEKQLQKDKQVYRATHRL
HumanGNA12[A12]   MSGVVRTLSRCLLPAEAGGARERRAGSGARDAEREARRRSRDIDALLARERRAVRRLVKI
HumanGNAQ[Aq]     ..MTLESIMACCL...............SEEAKE.ARRINDEIERQLRRDKRDARRELKL
HumanGNAI1[Ai]    ........MGCTL...............SAEDKA.AVERSKMIDRNLREDGEKAAREVKL
HumanGNAO1[Ao]    ........MGCTL...............SAEERA.ALERSKAIEKNLKEDGISAAKDVKL
```

```
                              α2                α3             α4
RatGnai1[Ai]      →    TT
                     40        50         60        70        80        90
RatGnai1[Ai]      LLLGAGESGKSTIVKQMKIIH..EAGYSEEE.CKQYKAVVYSNTIQSIIAIIRAMGRLKI
22522             LVLGNRNSGKTTFLKQFRIHH..GDGYPYLQ.RAILVPDILSNLADAIHIVMDNMARWDL
2931              LLLGTGESGKSTFLKQMKLIN..GKKFTSAE.LAGFKDTIYDNVYKGILFLLHARSSLNI
8947              LLLGTGESGKSTFLKQMKIIC..GQKFTVYE.IESFKDIIYDNIFKGVLFLIDARAQLNI
33116             ................MKLLSSINQTFPDVY.RAKFIPEIQRNLVQALTAILQFMDQIKL
9665              PVVGAGESGKSTLIKQMQILY..INGFNDEE.RKSKIKDIRGNIKDGMLAVTGAMKTLKP
6167              LLLGAGESGKSTIVKQMRILH..IDGFSNEE.KKQKAEDIRKNLRDAILTITSAMSNINP
5082              LLLGTGESGKSTFIKQMRIIH..GTGYSDDD.KRSFIKLIYQNIYLATYTLIRAMEVLKI
3755              LLLGTGESGKSTFIKQMRIIH..GSGYSDED.KRSFIKLVYQNIYLAIYTLILAMENLAI
1773              LLLGTGESGKSTIVKQMKLIV..NNGHSQAE.RLGFRSLIFSNTIQSLIVIIRAMSKLDI
13658             ...............................................MIDILYAVHHLNI
9715              LLLGAGESGKSTIMKQMKIIH..EGGFSSTD.NHHYKPIVYSNTIQSVAAILRAMHKLNI
1656              LLLGAGESGKSTIVKQMKIIH..EGGFTQED.NKQYKPVVYSNTTQSMIAILRAMTTLGI
12112             LLLGAGECGKSTILKQMTIIH..GKGYPEEE.RKEYVPIIYANVVQSMVTILNAMETLGI
19937             LLLGAGECGKSTILKQMSIIH..GGGYSDEE.KKEFIPIIYANIVQSMGAILKAMDILEI
47050complete     LLLGAGECGKSTILKQMSIIH..GDGYSESE.RKKFIPIIYSNIIQSVVTILKAMETLEI
4493              LLLGTGESGKSTILKQMKIIH..VNGFSKTE.KIEFISNIKRNVRDAIMAIVVSMNKLQI
HumanGNAS[As]     LLLGAGESGKSTIVKQMRILH..VNGFNGDSEKATKVQDIKNNLKEAIETIVAAMSNLVP
HumanGNA12[A12]   LLLGAGESGKSTFLKQMRIIH..GREFDQKA.LLEFRDTIFDNILKGSRVLVDARDKLGI
HumanGNAQ[Aq]     LLLGTGESGKSTFIKQMRIIH..GSGYSDED.KRGFTKLVYQNIFTAMQAMIRAMDTLKI
HumanGNAI1[Ai]    LLLGAGESGKSTIVKQMKIIH..EAGYSEEE.CKQYKAVVYSNTIQSIIAIIRAMGRLKI
HumanGNAO1[Ao]    LLLGAGESGKSTIVKQMKIIH..EDGFSGED.VKQYKPVVYSNTIQSLAAIVRAMDTLGI
```

```
                                 α5                                   α6
RatGnai1[Ai]
                     100                 110                 120
RatGnai1[Ai]      DFGDAARADDAR.............QLFVLAGA....AEEGF.........MTAELAGVI
22522             RFEDPYVQTLAKDFHNNCPRSPALSNNPHNLFKSIQNNSPRPFNRFCKTVNRITDFCLHI
2931              NFENYEETSEAEI....R.IEDHFKRNKEIHKEKA..TTGQI....IWSEEEFLKLVDDF
8947              DWNDNETAN.SAE....E.IERYFDETKLIHRQRR..QSKQL....LWKENEFLEIVQRL
33116             DFKHPNAHLHTAKKELYE.IRDQIDKNPDTIEKYA..NSTEM....D....VRNNFYDNC
9665              PVDLEHAEN..Q.............TLLDFIQHNA..VKPDF....T....YTKEFFDYC
6167              PTKLGNPEN..Q.............KFLDYMQHTA..SKPDF....R....YPSEFYEYC
5082              PYENPDNLEYAK.............DLRDIDY.....ETVTT.........FEPSHIVAT
3755              SYSNPENLSYID.............EIKNIDY.....ESVST.........LEPDHVIAI
1773              SFTNPERLNDAQ.............TLLQLAGT.V..ESNGP.........LNEELYSAM
13658             HFESSAREADEI.............LVNNTITT.G..MDEKP.........LNAQLCNAL
9715              SFENSAREADEI.............LVNNIMTT.M..KDQEP.........FTPQLNSAL
1656              SFGDIDRGADAK.............VVSDVIQA.M..EDTEP.........FSEELLEAM
12112             SMENEAMIEESK.............IVQKYMRN....AEEGE.........FPKELGSAL
19937             PLENANLGTEKN.............TVHESMKN....AEEGG.........MTINTIKAL
47050complete     KLNE.HLHSDKL.............LVIDQVKD....VDNGV.........LRQDAVESI
4493              TPKKFDLSEKIK.................FLQT.A..FSEQY....T....YPNTFFDVV
HumanGNAS[As]     PVELANPEN..Q.............FRVDYILSVM..NVPDF....D....FPPEFYEHA
HumanGNA12[A12]   PWQYSENEKHGM.............FLMAFENKAG..LPVEP....A....TFQLYVPAL
HumanGNAQ[Aq]     PYKYEHNKAHAQ.............LVREVDV.....EKVSA.........FENPYVDAI
HumanGNAI1[Ai]    DFGDSARADDAR.............QLFVLAGA....AEEGF.........MTAELAGVI
HumanGNAO1[Ao]    EYGDKERKADAK.............MVCDVVSR.M..EDTEP.........FSAELLSAM
```

```
                    α7                              α8        α9                α10
RatGnai1[Ai]
                    130        140         150         160         170        180
RatGnai1[Ai]        KRLWKDSGVQACFNRSREYQ...LNDSAAYYLNDLDRIAQPNYIPTQQDVLRTRVKTTGI
22522               KNISDQDDFQECLDC.KEFFKPSITYADLYFIQNIDRLIQADYTPTLQDIMVMRKPTYSV
2931                KLIWNDKGIKEAFNQRSKLMTESFSENSRFYLNKLDKIRVKDYQPSNEDIVWSRKPTDSI
8947                KAIWNDKSIQQTFLRRSEIITESFSENTRYYLNKIDEIGTLNYFPTDEDIVWTRKPTESI
33116               KALWRDPNILDTFQRSNEYQ...LIDSAQYFLDKIDLIRQPDYKPSDDDVLQCRTKTLGI
9665                YTLWIDAGVQATFARANEYQ...LIDSTKYFLDQVQTIAKDDYLPTDQDLLRCRVLTSGI
6167                AVLWADKGVLETYERSNEYQ...LIDCAKYFLDQALILGQQNYTPTEQDILRCRVLTSGI
5082                RSLWNDSGVKECYDRRREYQ...LTDSAKYYLDDLDRIVTPDYLPTLQDILRVRVPTTGI
3755                KSLWADVGVKECYDRRREFQ...LTDSAKYYLDSLDRIATPNFLPTLQDILRVRVPTTGI
1773                LNLWQDLGIQECFQRSAEYQ...LIDSAEYYLNSLERLSNSNYVPTEQDILRTRVKTAGI
13658               KSLWSDVGVQECFRTFNEYD...LNYSTQYFLDDIDRISAVDYEPTVQDILRSKMTNYGV
9715                KSLWSDIGVQECFRRSNEYQ...LNDSAQYFLDNVDRISAVDYKPSDQDILRTRIKTTGI
1656                KRLWADPGVQECFGRSNEYQ...LNDSAKYFLDDLDRLGAKDYMPTEQDILRTRVKTTGI
12112               ALLWKDDSVQNCYARAKEYQ...LNDSAGYYLDSLDRLSEPSYIPTEQDVLRSRVKTTGI
19937               TVLWKDTGVQEAFNRSKEYQ...LNDSAEYYLIDLERIASDNYIPTVQDILRSRIKTTGI
47050complete       KKLWQNVEVQQCVQRAKEYN...LNDSAEYYLNDIDRLRGDNYLPNDQDILRSRVKTTGI
4493                SELWQDEDVQASFLRSNEYQ...LIDSAKYFLDHIHIIRQNDYIPSLQDILRCRKMTSGI
HumanGNAS[As]       KALWEDEGVRACYERSNEYQ...LIDCAQYFLDKIDVIKQADYVPSDQDLLRCRVLTSGI
HumanGNA12[A12]     SALWRDSGIREAFSRRSEFQ...LGESVKYFLDNLDRIGQLNYFPSKQDILLARKATKGI
HumanGNAQ[Aq]       KSLWNDPGIQECYDRRREYQ...LSDSTKYYLNDLDRVADPAYLPTQQDVLRVRVPTTGI
HumanGNAI1[Ai]      KRLWKDSGVQACFNRSREYQ...LNDSAAYYLNDLDRIAQPNYIPTQQDVLRTRVKTTGI
HumanGNAO1[Ao]      MRLWGDSGIQECFNRSREYQ...LNDSAKYYLDSLDRIGAADYQPTEQDILRTRVKTTGI
```

```
                    β2               β3                  α11  η1          β4     η2
RatGnai1[Ai]        ------->T.....T------->              ..........      ------->.....
                      190              200                  210           220         230
RatGnai1[Ai]        VETHFTFK.....DLHFKMFDVGGQRSERKKWIHCF.EGVTAIIFCVALSDYDLVLAE..
22522               KDFIFNLG.....GITLRFIDLGGYKCQRKKFIHYF.EGVSTVFYFCALSEYSEIIEE..
2931                IEEEIDIH....GA.CIVFIDVGGQTKERKKWCQVF.QDMNSVLFLIASSHFDEFYYDKM
8947                IETEIEVS....NDIILVFIDVGGQTKERRKWCQLF.HDMKSVLFLIACSHFDEEYMDRF
33116               HTEIINYN.....NVNFELVDVGGQRDQRAKWIEALSDGVTAVIFLTDVSAYDMVLAE..
9665                FETKFTMN.....KVCFHMFDVGGQREERRKWIQVF.SDVTAVIFVCACSGYDMTLRE..
6167                FETKFTVD.....KVNFHMFDVGGQRDERRKWIQCF.NDVTAIIFVTACSSYNMVLRE..
5082                IEYPFDLD.....SIIFRMVDVGGQRSERRKWIHCF.EGVMTMIYVASISEYDQFLIE..
3755                IEYPFDLD.....SIIFRMVDVGGQRSERRKWIHCF.ENVTSIMFLVALSEYDQGLVE..
1773                IETTVEFK.....ELIMKIVDVGGQRAERKKWIHCF.DNVDAIIFIVALNEYDLRLRE..
13658               CECQIKYK.....ERMFTIFDTSVRLS.DRKWRICF.EDPSALIFCVSLTEYLK......
9715                VEIQFKFK.....NMNFKIFDVGGQRSERKKWIHCF.EDVTAIIFCVSLSEYDQVLVE..
1656                VEVHFMFK.....NMNFKLFDVGGQRSERKKWIHCF.EDVTAIIFCVAMSEYDQVLHE..
12112               VETHFHFK.....DLDFKVFDVGGQRSERKKWMHCF.EGVTAILFLVAMSEYDLKLVE..
19937               IETYFHFK.....DLDFKVFDVGGQRSERKKWMHCF.EGVTAILFLVALSEYDLRLVE..
47050complete       IETEFHFK.....NLDFKVYDVGGQRSERKKWMHCF.EGVTAILFLIAMSEYDLKLVE..
4493                TEISFEVREKKKNKVNFKVFDVGGQQGERKRWIQLF.GEVTAILFLADCSSFDQTLRE..
HumanGNAS[As]       FETKFQVD.....KVNFHMFDVGGQRDERRKWIQCF.NDVTAIIFVVASSSYNMVIRE..
HumanGNA12[A12]     VEHDFVIK.....KIPFKMVDVGGQRSQRQKWFQCF.DGITSILFMVSSSEYDQVLME..
HumanGNAQ[Aq]       IEYPFDLQ.....SVIFRMVDVGGQRSERRKWIHCF.ENVTSIMFLVALSEYDQVLVE..
HumanGNAI1[Ai]      VETHFTFK.....DLHFKMFDVGGQRSERKKWIHCF.EGVTAIIFCVALSDYDLVLAE..
HumanGNAO1[Ao]      VETHFTFK.....NLHFRLFDVGGQRSERKKWIHCF.EDVTAIIFCVALSGYDQVLHE..
```

```
                             α12             η3       β5          α13          η4
RatGnai1[Ai]
                                250          260         270          280          290
RatGnai1[Ai]        .DEEMNRMHESMKLFDSICNNKWFTDTSIILFLNKKDLFEEKIK..KSPLTICYPEYAGS
22522               ..TTKNKLNNSLENFEEIINNKYLWRKDFVIFLNKSDLFKSKVE..NLSINVFFSDFEGN
2931                SKEYRNKLREAMRVFEDLINVQYFLSVSVIIFFNKTDVLTEKVTNKISDIRSEFTEYPEN
8947                TLQKRNKLKEAMFVFEELINQNAFLRVSVLLFFNKTDILSEKIKSHQSNIGKDFQDFPGE
33116               .DQTTNRLRESVSLLGQVWTKNPLRDKSIILFLNKQDKLEMKVRKGRTQIETYFPEFKQE
9665                .AQKQNRLRECISLFSEVWGNRYLRQTSIILFLNKQDLFVQKLTSGKTKIVDFFPEFAEY
6167                .DASQNRLRESLELLKSIWNNRWLRNISVILFLNKQDVLKEKVLAGKSKIEDYFPDYLRY
5082                .DNEINRMFESIKLFDSVCNNQWFSKCSIILFLNKTDLFKLKIV..KSPLTVCFPEYKGN
3755                .SDNDNRMEESKALFRTIITYPWFHNASVILFLNKKDLLEEKII..YSHLVDYFPEYEGP
1773                .DPEVNRMMESLRLFDSMCNNVVFKDTCMILFLNKRDLFEVKIK..KSPLSICFEEYADE
13658               ....EDEMACSMRYFNSIIQFKWFQNSSFILFLNKKDLFKRKLL..DHPITCCFPEYTGL
9715                .DDATNRMQESLFLFESICNNVWFLHTSFILFLNKKDLFLEKLQ..ICPITFCFPEYKGP
1656                .DETTNRMQESLKLFDSICNNKWFTQTSIILFLNKKDLFAEKIK..RSPLTVCFSEYTGR
12112               .DSSTNRMHESMRLFDSICNSQWFVNTSIILFLNKKDLFGEKVV..KSPLTVCFPEYTGA
19937               .DSTTNRMHESMKLFDSICNSPWFVNTSVILFLNKVDLFEIKIQ..RSPLTICFPEYLGP
47050complete       .DQTTNRMHESMKLFESICNSQWFTSTSIILFLNKKDLFMKKIE..ISPITICFKDYNGP
4493                .DRSKNRLIDSLEVFYQAWMNRYLQNVPIIVFVNKIDMLELKIQNEHS.IESMINEITNL
HumanGNAS[As]       .DNQTNRLQEALNLFKSIWNNRWLRTISVILFLNKQDLLAEKVLAGKSKIEDYFPEFARY
HumanGNA12[A12]     .DRRTNRLVESMNIFETIVNNKLFFNVSIILFLNKMDLLVEKVK..TVSIKKHFPDFRGD
HumanGNAQ[Aq]       .SDNENRMEESKALFRTIITYPWFQNSSVILFLNKKDLLEEKIM..YSHLVDYFPEYDGP
HumanGNAI1[Ai]      .DEEMNRMHESMKLFDSICNNKWFTDTSIILFLNKKDLFEEKIK..KSPLTICYPEYAGS
HumanGNAO1[Ao]      .DETTNRMHESLMLFDSICNNKFFIDTSIILFLNKKDLFGEKIK..KSPLTICFPEYTGP
```

```
RatGnai1[Ai]

RatGnai1[Ai]       ............................................................
22522              PS..........................................................
2931               FDPHNIV.....................................................
8947               SDPYNLV.....................................................
33116              KCKHIFELIKDLKAARGKRSKKESDIWEKYFSYFLPQMRDQKKTENPEDATRQQRET...
9665               ............................................................
6167               ............................................................
5082               ............................................................
3755               ............................................................
1773               ............................................................
13658              ............................................................
9715               ............................................................
1656               ............................................................
12112              ............................................................
19937              ............................................................
47050complete      ............................................................
4493               TPHSTKNSAKQLNNRRSSLNINHKTNN........DNMTSCSKCENSHPITDNNKCIHLC
HumanGNAS[As]      ............................................................
HumanGNA12[A12]    ............................................................
HumanGNAQ[Aq]      ............................................................
HumanGNAI1[Ai]     ............................................................
HumanGNAO1[Ao]     ............................................................


RatGnai1[Ai]                                         ...........................

RatGnai1[Ai]       ..............................NTY...........................
22522              ............................................................
2931               ..............................Q...........................VQ
8947               ..............................D...........................VQ
33116              .......QIIN..............EYS..QIQSQVAKVFNDGSVDKWLTDGDDDDIFSH
9665               ..............................QSK............NTFDS..........
6167               ..............................TLP............ADVQC..........
5082               ..............................NNF...........................
3755               ..............................QRD...........................
1773               ..............................NTY...........................
13658              ..............................QEY...........................
9715               ..............................QEY...........................
1656               ..............................QTY...........................
12112              ..............................NIY...........................
19937              ..............................NTF...........................
47050complete      ..............................HNY...........................
4493               PESNSNSKICDRKISIRTIPSCSSRRFSKDNNFEFVAQTFSENPWGKYQPSNEECKEFVN
HumanGNAS[As]      ..............................TTP............EDATP..........
HumanGNA12[A12]    ..............................PHR...........................
HumanGNAQ[Aq]      ..............................QRD...........................
HumanGNAI1[Ai]     ..............................NTY...........................
HumanGNAO1[Ao]     ..............................NTY...........................


                           α14
RatGnai1[Ai]       ................................
                                                     300           310

RatGnai1[Ai]       ................................EEAAAYIQCQFEDL..N.......KRKD
22522              ..............................DVEDGINFFRMKFLSLKPA.......NAPK
2931               QFLVDSFVSFVDNPGA...............HP.RSQYSAGNQNQGR.ASIAG.QAGVPK
8947               MFIVDSFVKLIDNPYG...............NDKRASYIRSNSVSGN.KRVVSPVPMQIP
33116              LMKNDEFISFH..........KRIMNITFYQVVSITFFIENQFLHQC.........EQTS
9665               ..........Q..............SDEPSEVTYAKNFVKDKFMST.TK.......KEEG
6167               ..........H.............NHIEEAEFTRAKYFFRDEFLKITTT.......GNDG
5082               ................................EDASEYVRMTFEML..N.......K.KK
3755               ...............................AEAARDFILKMFIEL..N.......P.DQ
1773               ................................EKAVTYIKFKFESL..N.......KYRQ
13658              ................................DPSIEYIEKRFRNE..V.......K.DS
9715               ................................DSSVSYIEQCFRSK..N.......K.DV
1656               ................................EEAAAYIQASFEAK..N.......N.SP
12112              ................................EEASAYIQSQFEEL..N.......KKKE
19937              ................................VDTSNYIREKFEGL..N.......KKKN
47050complete      ................................EETSNFIRLKFEDL..N.......KSKD
4493               SFPVDQ.VDLHSSGTHAAKKKSKDVHNIHPDTIKTACYIKNIFAQITRNHPKNCDMKISN
HumanGNAS[As]      ..........E..............PGEDPRVTRAKYFIRDEFLRISTA.......SGDG
HumanGNA12[A12]    ...............................LEDVQRYLVQCFDRK..R.......R.NR
HumanGNAQ[Aq]      ...............................AQAAREFILKMFVDL..N.......P.DS
HumanGNAI1[Ai]     ................................EEAAAYIQCQFEDL..N.......KRKD
HumanGNAO1[Ao]     ................................EDAAAYIQAQFESK..N.......R.SP
```

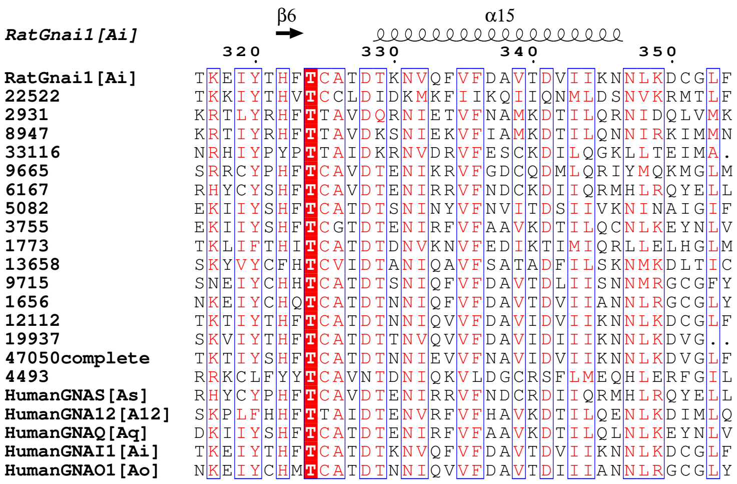
RatGnai1[Ai]
β6
α15
320
330
340
350
RatGnai1[Ai] TKEIYTHFTCATDTKNVQFVFDAVTDVIIKNNLKDCGLF
22522 TKKIYTHVTCCLDIDKMKFIIKQIIQNMLDSNVKRMTLF
2931 KRTLYRHFTTAVDQRNIETVFNAMKDTILQRNIDQLVMK
8947 KRTIYRHFTTAVDKSNIEKVFIAMKDTILQNNIRKIMMN
33116 NRHIYPYPTTAIDKRNVDRVFESCKDILQGKLLTEIMA.
9665 SRRCYPHFTCAVDTENIKRVFGDCQDMLQRIYMQKMGLM
6167 RHYCYSHFTCAVDTENIRRVFNDCKDIIQRMHLRQYELL
5082 EKIIYSHFTCATDTSNINYVFNVITDSIIVKNINAIGIF
3755 EKIIYSHFTCGTDTENIRFVFAAVKDTILQCNLKEYNLV
1773 TKLIFTHITCATDTDNVKNVFEDIKTIMIQRLLELHGLM
13658 SKYVYCFHTCVIDTANIQAVFSATADFILSKNMKDLTIC
9715 SNEIYCHHTCATDTSNIQFVFDAVTDLIISNNMRGCGFY
1656 NKEIYCHQTCATDTNNIQFVFDAVTDVIIANNLRGCGLY
12112 TKTIYTHFTCATDTNNIQVVFDAVIDVIIKNNLKDCGLF
19937 SKVIYTHFTCATDTTNVQVVFDAVIDIIIKNNLKDVG..
47050complete TKTIYSHFTCATDTNNIEVVFNAVIDVIIKNNLKDVGLF
4493 RRKCLFYYTCAVNTDNIQKVLDGCRSFLMEQHLERFGIL
HumanGNAS[As] RHYCYPHFTCAVDTENIRRVFNDCRDIIQRMHLRQYELL
HumanGNA12[A12] SKPLFHHFTTAIDTENVRFVFHAVKDTILQENLKDIMLQ
HumanGNAQ[Aq] DKIIYSHFTCATDTENIRFVFAAVKDTILQLNLKEYNLV
HumanGNAI1[Ai] TKEIYTHFTCATDTKNVQFVFDAVTDVIIKNNLKDCGLF
HumanGNAO1[Ao] NKEIYCHMTCATDTNNIQVVFDAVTDIIIANNLRGCGLY

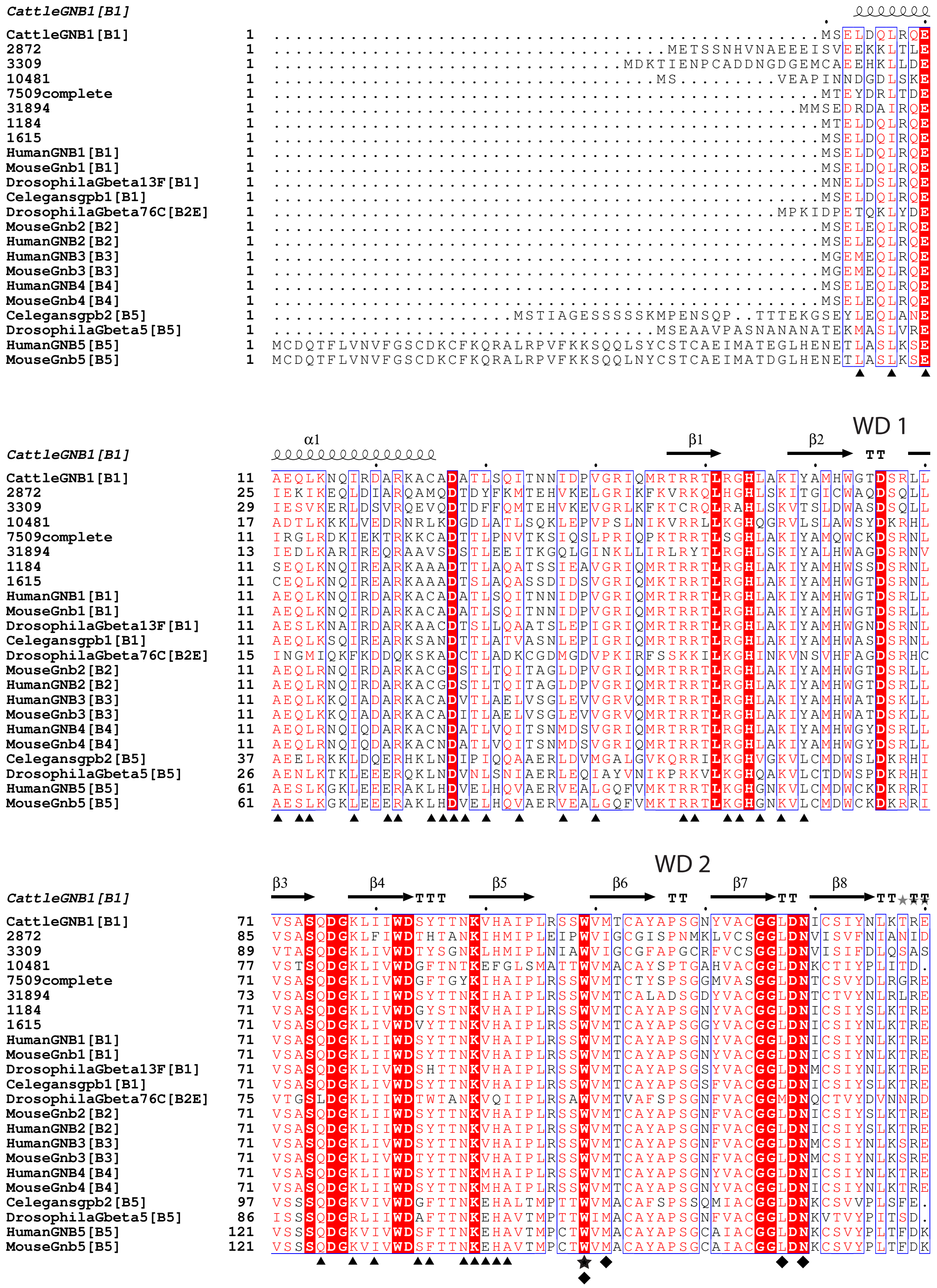

CattleGNB1[B1]
CattleGNB1[B1] 1 ....MSELDQLRQE
2872 1 ....................................METSSNHVNAEEEISVEEKKLTLE
3309 1 ................................MDKTIENPCADDNGDGEMCAEEHKLLDE
10481 1 ....MS.........VEAPINNDGDLSKE
7509complete 1 ....MTEYDRLTDE
31894 1 ....MMSEDRDAIRQE
1184 1 ....MTELDQLRQE
1615 1 ....MSELDQIRQE
HumanGNB1[B1] 1 ....MSELDQLRQE
MouseGnb1[B1] 1 ....MSELDQLRQE
DrosophilaGbeta13F[B1] 1 ....MNELDSLRQE
Celegansgpb1[B1] 1 ....MSELDQLRQE
DrosophilaGbeta76C[B2E] 1 ....MPKIDPETQKLYDE
MouseGnb2[B2] 1 ....MSELEQLRQE
HumanGNB2[B2] 1 ....MSELEQLRQE
HumanGNB3[B3] 1 ....MGEMEQLRQE
MouseGnb3[B3] 1 ....MGEMEQLRQE
HumanGNB4[B4] 1 ....MSELEQLRQE
MouseGnb4[B4] 1 ....MSELEQLRQE
Celegansgpb2[B5] 1 ......................MSTIAGESSSSSKMPENSQP..TTTEKGSEYLEQLANE
DrosophilaGbeta5[B5] 1 ...................................MSEAAVPASNANANATEKMASLVRE
HumanGNB5[B5] 1 MCDQTFLVNVFGSCDKCFKQRALRPVFKKSQQLSYCSTCAEIMATEGLHENETLASLKSE
MouseGnb5[B5] 1 MCDQTFLVNVFGSCDKCFKQRALRPVFKKSQQLNYCSTCAEIMATDGLHENETLASLKSE
WD 1
α1
β1
β2
TT
CattleGNB1[B1]
CattleGNB1[B1] 11 AEQLKNQIRDARKACADATLSQITNNIDPVGRIQMRTRRTLRGHLAKIYAMHWGTDSRLL
2872 25 IEKIKEQLDIARQAMQDTDYFKMTEHVKELGRIKFKVRKQLHGHLSKITGICWAQDSQLL
3309 29 IESVKERLDSVRQEVQDTDFFQMTEHVKEVGRLKFKTCRQLRAHLSKVTSLDWASDSQLL
10481 17 ADTLKKKLVEDRNRLKDGDLATLSQKLEPVPSLNIKVRRLLKGHQGRVLSLAWSYDKRHL
7509complete 11 IRGLRDKIEKTRKKCADTTLPNVTKSIQSLPRIQPKTRRTLSGHLAKIYAMQWCKDSRNL
31894 13 IEDLKARIREQRAAVSDSTLEEITKGQLGINKLLIRLRYTLRGHLSKIYALHWAGDSRNV
1184 11 SEQLKNQIREARKAAADTTLAQATSSIEAVGRIQMRTRRTLRGHLAKIYAMHWSSDSRNL
1615 11 CEQLKNQIREARKAAADTSLAQASSDIDSVGRIQMKTRRTLRGHLAKIYAMHWGTDSRNL
HumanGNB1[B1] 11 AEQLKNQIRDARKACADATLSQITNNIDPVGRIQMRTRRTLRGHLAKIYAMHWGTDSRLL
MouseGnb1[B1] 11 AEQLKNQIRDARKACADATLSQITNNIDPVGRIQMRTRRTLRGHLAKIYAMHWGTDSRLL
DrosophilaGbeta13F[B1] 11 AESLKNAIRDARKAACDTSLLQAATSLEPIGRIQMRTRRTLRGHLAKIYAMHWGNDSRNL
Celegansgpb1[B1] 11 AEQLKSQIREARKSANDTTLATVASNLEPIGRIQMRTRRTLRGHLAKIYAMHWASDSRNL
DrosophilaGbeta76C[B2E] 15 INGMIQKFKDDQKSKADCTLADKCGDMGDVPKIRFSSKKILKGHINKVNSVHFAGDSRHC
MouseGnb2[B2] 11 AEQLRNQIRDARKACGDSTLTQITAGLDPVGRIQMRTRRTLRGHLAKIYAMHWGTDSRLL
HumanGNB2[B2] 11 AEQLRNQIRDARKACGDSTLTQITAGLDPVGRIQMRTRRTLRGHLAKIYAMHWGTDSRLL
HumanGNB3[B3] 11 AEQLKKQIADARKACADVTLAELVSGLEVVGRVQMRTRRTLRGHLAKIYAMHWATDSKLL
MouseGnb3[B3] 11 AEQLKKQIADARKACADITLAELVSGLEVVGRVQMRTRRTLRGHLAKIYAMHWATDSKLL
HumanGNB4[B4] 11 AEQLRNQIQDARKACNDATLVQITSNMDSVGRIQMRTRRTLRGHLAKIYAMHWGYDSRLL
MouseGnb4[B4] 11 AEQLRNQIQDARKACNDATLVQITSNMDSVGRIQMRTRRTLRGHLAKIYAMHWGYDSRLL
Celegansgpb2[B5] 37 AEELRKKLDQERHKLNDIPIQQAAERLDVMGALGVKQRRILKGHVGKVLCMDWSLDKRHI
DrosophilaGbeta5[B5] 26 AENLKTKLEEERQKLNDVNLSNIAERLEQIAYVNIKPRKVLKGHQAKVLCTDWSPDKRHI
HumanGNB5[B5] 61 AESLKGKLEEERAKLHDVELHQVAERVEALGQFVMKTRRTLKGHGNKVLCMDWCKDKRRI
MouseGnb5[B5] 61 AESLKGKLEEERAKLHDVELHQVAERVEALGQFVMKTRRTLKGHGNKVLCMDWCKDKRRI
WD 2
β3
β4
β5
β6
β7
β8
TTT
TT
TT
TT
TT
CattleGNB1[B1]
CattleGNB1[B1] 71 VSASQDGKLIIWDSYTTNKVHAIPLRSSWVMTCAYAPSGNYVACGGLDNICSIYNLKTRE
2872 85 VSASQDGKLFIWDTHTANKIHMIPLEIPWVIGCGISPNMKLVCSGGLDNVISVFNIANID
3309 89 VTASQDGKLIVWDTYSGNKLHMIPLNIAWVIGCGFAPGCRFVCSGGLDNVISIFDLQSAS
10481 77 VSTSQDGKLIVWDGFTNTKEFGLSMATTWVMACAYSPTGAHVACGGLDNKCTIYPLITD.
7509complete 71 VSASQDGKLIVWDGFTGYKIHAIPLRSSWVMTCTYSPSGGMVASGGLDNTCSVYDLRGRE
31894 73 VSASQDGKLIIWDSYTTNKIHAIPLRSSWVMTCALADSGDYVACGGLDNTCTVYNLRLRE
1184 71 VSASQDGKLIVWDGYSTNKVHAIPLRSSWVMTCAYAPSGNYVACGGLDNICSIYSLKTRE
1615 71 VSASQDGKLIVWDVYTTNKVHAIPLRSSWVMTCAYAPSGGYVACGGLDNICSIYNLKTRE
HumanGNB1[B1] 71 VSASQDGKLIIWDSYTTNKVHAIPLRSSWVMTCAYAPSGNYVACGGLDNICSIYNLKTRE
MouseGnb1[B1] 71 VSASQDGKLIIWDSYTTNKVHAIPLRSSWVMTCAYAPSGNYVACGGLDNICSIYNLKTRE
DrosophilaGbeta13F[B1] 71 VSASQDGKLIVWDSHTTNKVHAIPLRSSWVMTCAYAPSGSYVACGGLDNMCSIYNLKTRE
Celegansgpb1[B1] 71 VSASQDGKLIVWDSYTTNKVHAIPLRSSWVMTCAYAPSGSFVACGGLDNICSIYSLKTRE
DrosophilaGbeta76C[B2E] 75 VTGSLDGKLIIWDTWTANKVQIIPLRSAWVMTVAFSPSGNFVACGGMDNQCTVYDVNNRD
MouseGnb2[B2] 71 VSASQDGKLIIWDSYTTNKVHAIPLRSSWVMTCAYAPSGNFVACGGLDNICSIYSLKTRE
HumanGNB2[B2] 71 VSASQDGKLIIWDSYTTNKVHAIPLRSSWVMTCAYAPSGNFVACGGLDNICSIYSLKTRE
HumanGNB3[B3] 71 VSASQDGKLIVWDSYTTNKVHAIPLRSSWVMTCAYAPSGNFVACGGLDNMCSIYNLKSRE
MouseGnb3[B3] 71 VSASQDGKLIVWDTYTTNKVHAIPLRSSWVMTCAYAPSGNFVACGGLDNMCSIYNLKSRE
HumanGNB4[B4] 71 VSASQDGKLIIWDSYTTNKMHAIPLRSSWVMTCAYAPSGNYVACGGLDNICSIYNLKTRE
MouseGnb4[B4] 71 VSASQDGKLIIWDSYTTNKMHAIPLRSSWVMTCAYAPSGNYVACGGLDNICSIYNLKTRE
Celegansgpb2[B5] 97 VSSSQDGKVIVWDGFTTNKEHALTMPTTWVMACAFSPSSQMIACGGLDNKCSVVPLSFE.
DrosophilaGbeta5[B5] 86 ISSSQDGRLIIWDAFTTNKEHAVTMPTTWIMACAYAPSGNFVACGGLDNKVTVYPITSD.
HumanGNB5[B5] 121 VSSSQDGKVIVWDSFTTNKEHAVTMPCTWVMACAYAPSGCAIACGGLDNKCSVYPLTFDK
MouseGnb5[B5] 121 VSSSQDGKVIVWDSFTTNKEHAVTMPCTWVMACAYAPSGCAIACGGLDNKCSVYPLTFDK

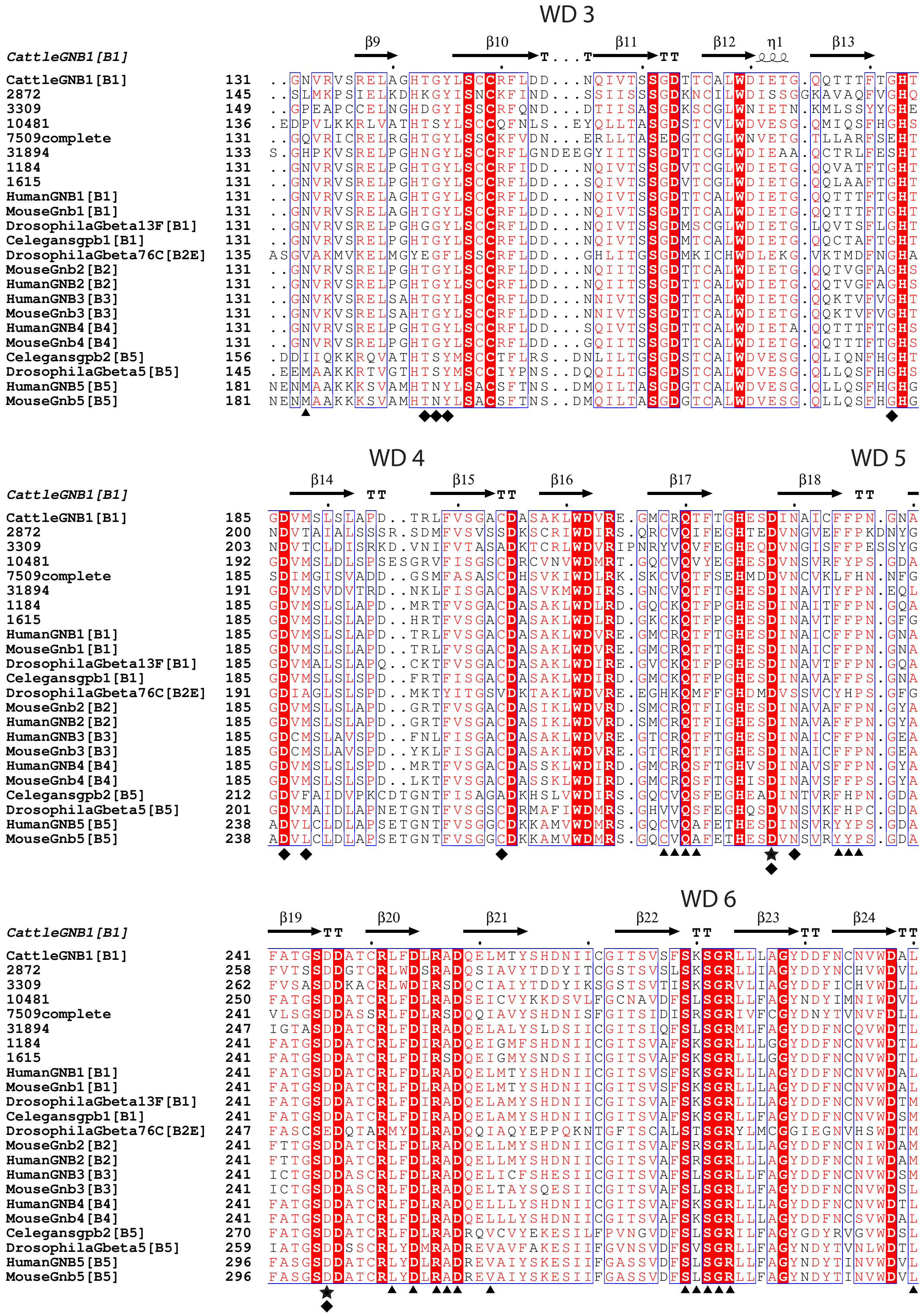
WD 3
β9 β10 β11 β12 η1 β13
TT TT
CattleGNB1[B1]
CattleGNB1[B1] 131 ..GNVRVSRELAGHTGYLSCCRFLDD...NQIVTSSGDTTCALWDIETG.QQTTTFTGHT
2872 145 ..SLMKPSIELKDHKGYISNCKFIND...SSIISSSGDKNCILWDISSGGKAVAQFVGHQ
3309 149 ..GPEAPCCELNGHDGYISSCRFQND...DTIISASGDKSCGLWNIETN.KMLSSYYGHE
10481 136 .EDPVLKKRLVATHTSYLSCCQFNLS..EYQLLTASGDSTCVLWDVESG.QMIQSFHGHS
7509complete 131 ..GQVRICRELRGHTGYLSSCKFVDN...ERLLTASEDGTCGLWNVETG.TLLARFSEHT
31894 133 S.GHPKVSRELPGHNGYLSCCRFLGNDEEGYIITSSGDTTCGLWDIEAA.QCTRLFESHT
1184 131 ..GNVRVSRELPGHTGYLSCCRFIDD...NQIVTSSGDVTCGLWDIETG.QQVATFTGHT
1615 131 ..GNVRVSRELPGHTGYLSCCRFLDD...SQIVTSSGDVTCGLWDIETG.QQLAAFTGHT
HumanGNB1[B1] 131 ..GNVRVSRELAGHTGYLSCCRFLDD...NQIVTSSGDTTCALWDIETG.QQTTTFTGHT
MouseGnb1[B1] 131 ..GNVRVSRELAGHTGYLSCCRFLDD...NQIVTSSGDTTCALWDIETG.QQTTTFTGHT
DrosophilaGbeta13F[B1] 131 ..GNVRVSRELPGHGGYLSCCRFLDD...NQIVTSSGDMSCGLWDIETG.LQVTSFLGHT
Celegansgpb1[B1] 131 ..GNVRVSRELPGHTGYLSCCRFLDD...NQIVTSSGDMTCALWDIETG.QQCTAFTGHT
DrosophilaGbeta76C[B2E] 135 ASGVAKMVKELMGYEGFLSSCRFLDD...GHLITGSGDMKICHWDLEKG.VKTMDFNGHA
MouseGnb2[B2] 131 ..GNVRVSRELPGHTGYLSCCRFLDD...NQIITSSGDTTCALWDIETG.QQTVGFAGHS
HumanGNB2[B2] 131 ..GNVRVSRELPGHTGYLSCCRFLDD...NQIITSSGDTTCALWDIETG.QQTVGFAGHS
HumanGNB3[B3] 131 ..GNVKVSRELSAHTGYLSCCRFLDD...NNIVTSSGDTTCALWDIETG.QQKTVFVGHT
MouseGnb3[B3] 131 ..GNVKVSRELSAHTGYLSCCRFLDD...NNIVTSSGDTTCALWDIETG.QQKTVFVGHT
HumanGNB4[B4] 131 ..GNVRVSRELPGHTGYLSCCRFLDD...SQIVTSSGDTTCALWDIETA.QQTTTFTGHS
MouseGnb4[B4] 131 ..GNVRVSRELPGHTGYLSCCRFLDD...GQIITSSGDTTCALWDIETG.QQTTTFTGHS
Celegansgpb2[B5] 156 .DDIIQKKRQVATHTSYMSCCTFLRS..DNLILTGSGDSTCAIWDVESG.QLIQNFHGHT
DrosophilaGbeta5[B5] 145 .EEMAAKKRTVGTHTSYMSCCIYPNS..DQQILTGSGDSTCALWDVESG.QLLQSFHGHS
HumanGNB5[B5] 181 NENMAAKKKSVAMHTNYLSACSFTNS..DMQILTASGDGTCALWDVESG.QLLQSFHGHG
MouseGnb5[B5] 181 NENMAAKKKSVAMHTNYLSACSFTNS..DMQILTASGDGTCALWDVESG.QLLQSFHGHG
WD 4
WD 5
β14 β15 β16 β17 β18
TT TT TT
CattleGNB1[B1]
CattleGNB1[B1] 185 GDVMSLSLAPD..TRLFVSGACDASAKLWDVRE.GMCRQTFTGHESDINAICFFPN.GNA
2872 200 NDVTAIALSSSR.SDMFVSVSSDKSCRIWDIRS.QRCVQIFEGHTEDVNGVEFFPKDNYG
3309 203 NDVTCLDISRKD.VNIFVTASADKTCRLWDVRIPNRYVQVFEGHEQDVNGISFFPESSYG
10481 192 GDVMSLDLSPSESGRVFISGSCDRCVNVWDMRT.GQCVQVYEGHESDVNSVRFYPS.GDA
7509complete 185 SDIMGISVADD..GSMFASASCDHSVKIWDLRK.SKCVQTFSEHMDDVNCVKLFHN.NFG
31894 191 GDVMSVDVTRD..NKLFISGACDASVKMWDIRS.GNCVQTFTGHESDINAVTYFPN.EQL
1184 185 GDVMSLSLAPD..MRTFVSGACDASAKLWDLRD.GQCKQTFPGHESDINAITFFPN.GQA
1615 185 GDVMSLSLAPD..HRTFVSGACDASAKLWDLRD.GKCKQTFPGHESDINAVTFFPN.GFG
HumanGNB1[B1] 185 GDVMSLSLAPD..TRLFVSGACDASAKLWDVRE.GMCRQTFTGHESDINAICFFPN.GNA
MouseGnb1[B1] 185 GDVMSLSLAPD..TRLFVSGACDASAKLWDVRE.GMCRQTFTGHESDINAICFFPN.GNA
DrosophilaGbeta13F[B1] 185 GDVMALSLAPQ..CKTFVSGACDASAKLWDIRE.GVCKQTFPGHESDINAVTFFPN.GQA
Celegansgpb1[B1] 185 GDVMSLSLSPD..FRTFISGACDASAKLWDIRD.GMCKQTFPGHESDINAVAFFPS.GNA
DrosophilaGbeta76C[B2E] 191 GDIAGLSLSPD..MKTYITGSVDKTAKLWDVRE.EGHKQMFFGHDMDVSSVCYHPS.GFG
MouseGnb2[B2] 185 GDVMSLSLAPD..GRTFVSGACDASIKLWDVRD.SMCRQTFIGHESDINAVAFFPN.GYA
HumanGNB2[B2] 185 GDVMSLSLAPD..GRTFVSGACDASIKLWDVRD.SMCRQTFIGHESDINAVAFFPN.GYA
HumanGNB3[B3] 185 GDCMSLAVSPD..FNLFISGACDASAKLWDVRE.GTCRQTFTGHESDINAICFFPN.GEA
MouseGnb3[B3] 185 GDCMSLAVSPD..YKLFISGACDASAKLWDVRE.GTCRQTFTGHESDINAICFFPN.GEA
HumanGNB4[B4] 185 GDVMSLSLSPD..MRTFVSGACDASSKLWDIRD.GMCRQSFTGHVSDINAVSFFPN.GYA
MouseGnb4[B4] 185 GDVMSLSLSPD..LKTFVSGACDASSKLWDIRD.GMCRQSFTGHISDINAVSFFPS.GYA
Celegansgpb2[B5] 212 GDVFAIDVPKCDTGNTFISAGADKHSLVWDIRS.GQCVQSFEGHEADINTVRFHPN.GDA
DrosophilaGbeta5[B5] 201 GDVMAIDLAPNETGNTFVSGSCDRMAFIWDMRS.GHVVQSFEGHQSDVNSVKFHPC.GDA
HumanGNB5[B5] 238 ADVLCLDLAPSETGNTFVSGGCDKKAMVWDMRS.GQCVQAFETHESDINSVRYYPS.GDA
MouseGnb5[B5] 238 ADVLCLDLAPSETGNTFVSGGCDKKAMVWDMRS.GQCVQAFETHESDVNSVRYYPS.GDA
WD 6
β19 β20 β21 β22 β23 β24
TT TT TT TT
CattleGNB1[B1]
CattleGNB1[B1] 241 FATGSDDATCRLFDLRADQELMTYSHDNIICGITSVSFSKSGRLLLAGYDDFNCNVWDAL
2872 258 FVTSSDDGTCRLWDSRADQSIAVYTDDYITCGSTSVSLSKSGRLLIAGYDDYNCHVWDVL
3309 262 FVSASDDKACRLWDIRSDQCIAIYTDDYIKSGSTSVTISKSGRVLIAGYDDFICHVWDLL
10481 250 FATGSDDATCRLFDLRADSEICVYKKDSVLFGCNAVDFSLSGRLLFAGYNDYIMNIWDVL
7509complete 241 VLSGSDDASSRLFDLRSDQQIAVYSHDNISFGITSIDISRSGRIVFCGYDNYTVNVFDLL
31894 247 IGTASDDATCRLFDIRADQELALYSLDSIICGITSIQFSLSGRMLFAGYDDFNCQVWDTL
1184 241 FATGSDDATCRLFDIRADQEIGMFSHDNIICGITSVAFSKSGRLLLGGYDDFNCNVWDTL
1615 241 FATGSDDATCRLFDIRSDQEIGMYSNDSIICGITSVAFSKSGRLLLGGYDDFNCNVWDTL
HumanGNB1[B1] 241 FATGSDDATCRLFDLRADQELMTYSHDNIICGITSVSFSKSGRLLLAGYDDFNCNVWDAL
MouseGnb1[B1] 241 FATGSDDATCRLFDLRADQELMTYSHDNIICGITSVSFSKSGRLLLAGYDDFNCNVWDAL
DrosophilaGbeta13F[B1] 241 FATGSDDATCRLFDIRADQELAMYSHDNIICGITSVAFSKSGRLLLAGYDDFNCNVWDTM
Celegansgpb1[B1] 241 FATGSDDATCRLFDIRADQELAMYSHDNIICGITSVAFSKSGRLLFAGYDDFNCNVWDSM
DrosophilaGbeta76C[B2E] 247 FASCSEDQTARMYDLRADQQIAQYEPPQKNTGFTSCALSTSGRYLMCGGIEGNVHSWDTM
MouseGnb2[B2] 241 FTTGSDDATCRLFDLRADQELLMYSHDNIICGITSVAFSRSGRLLLAGYDDFNCNIWDAM
HumanGNB2[B2] 241 FTTGSDDATCRLFDLRADQELLMYSHDNIICGITSVAFSRSGRLLLAGYDDFNCNIWDAM
HumanGNB3[B3] 241 ICTGSDDASCRLFDLRADQELICFSHESIICGITSVAFSLSGRLLFAGYDDFNCNVWDSM
MouseGnb3[B3] 241 ICTGSDDASCRLFDLRADQELTAYSQESIICGITSVAFSLSGRLLFAGYDDFNCNVWDSL
HumanGNB4[B4] 241 FATGSDDATCRLFDLRADQELLLYSHDNIICGITSVAFSKSGRLLLAGYDDFNCNVWDTL
MouseGnb4[B4] 241 FATGSDDATCRLFDLRADQELLLYSHDNIICGITSVAFSKSGRLLLAGYDDFNCSVWDAL
Celegansgpb2[B5] 270 FATGSDDATCRLFDLRADRQVCVYEKESIIFPVNGVDFSLSGRILFAGYGDYRVGVWDSL
DrosophilaGbeta5[B5] 259 IATGSDDSSCRLYDMRADREVAVFAKESIIFGVNSVDFSVSGRLLFAGYNDYTVNLWDTL
HumanGNB5[B5] 296 FASGSDDATCRLYDLRADREVAIYSKESIIFGASSVDFSLSGRLLFAGYNDYTINVWDVL
MouseGnb5[B5] 296 FASGSDDATCRLYDLRADREVAIYSKESIIFGASSVDFSLSGRLLFAGYNDYTINVWDVL

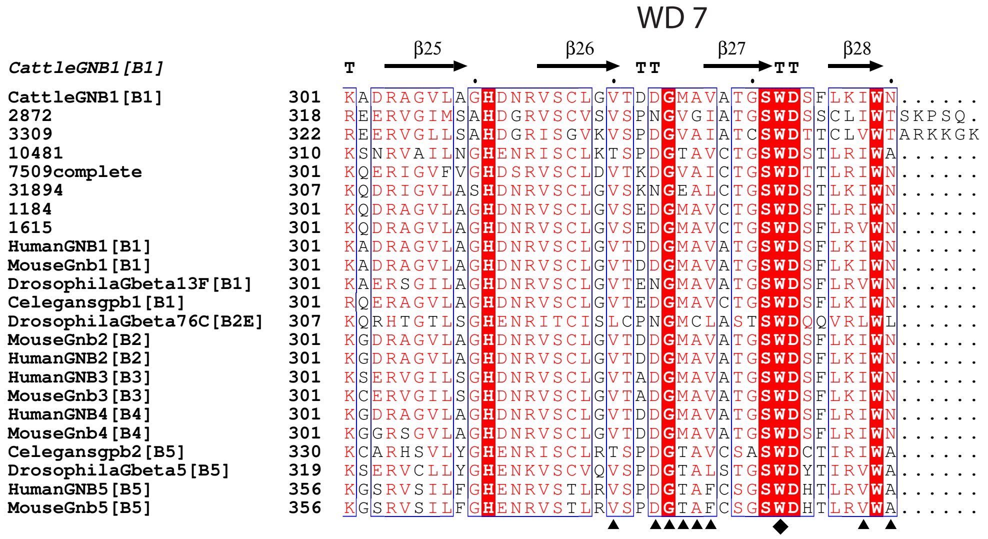
WD 7
β25
β26
β27
β28
CattleGNB1[B1]
CattleGNB1[B1] 301 KADRAGVLAGHDNRVSCLGVTDDGMAVATGSWDSFLKIWN......
2872 318 REERVGIMSAHDGRVSCVSVSPNGVGIATGSWDSSCLIWTSKPSQ.
3309 322 REERVGLLSAHDGRISGVKVSPDGVAIATCSWDTTCLVWTARKKGK
10481 310 KSNRVAILNGHENRISCLKTSPDGTAVCTGSWDSTLRIWA......
7509complete 301 KQERIGVFVGHDSRVSCLDVTKDGVAICTGSWDTTLRIWN......
31894 307 KQDRIGVLASHDNRVSCLGVSKNGEALCTGSWDSTLKIWN......
1184 301 KQDRAGVLAGHDNRVSCLGVSEDGMAVCTGSWDSFLRIWN......
1615 301 KQDRAGVLAGHDNRVSCLGVSEDGMAVCTGSWDSFLRVWN......
HumanGNB1[B1] 301 KADRAGVLAGHDNRVSCLGVTDDGMAVATGSWDSFLKIWN......
MouseGnb1[B1] 301 KADRAGVLAGHDNRVSCLGVTDDGMAVATGSWDSFLKIWN......
DrosophilaGbeta13F[B1] 301 KAERSGILAGHDNRVSCLGVTENGMAVATGSWDSFLRVWN......
Celegansgpb1[B1] 301 RQERAGVLAGHDNRVSCLGVTEDGMAVCTGSWDSFLKIWN......
DrosophilaGbeta76C[B2E] 307 KQRHTGTLSGHENRITCISLCPNGMCLASTSWDQQVRLWL......
MouseGnb2[B2] 301 KGDRAGVLAGHDNRVSCLGVTDDGMAVATGSWDSFLKIWN......
HumanGNB2[B2] 301 KGDRAGVLAGHDNRVSCLGVTDDGMAVATGSWDSFLKIWN......
HumanGNB3[B3] 301 KSERVGILSGHDNRVSCLGVTADGMAVATGSWDSFLKIWN......
MouseGnb3[B3] 301 KCERVGILSGHDNRVSCLGVTADGMAVATGSWDSFLKIWN......
HumanGNB4[B4] 301 KGDRAGVLAGHDNRVSCLGVTDDGMAVATGSWDSFLRIWN......
MouseGnb4[B4] 301 KGGRSGVLAGHDNRVSCLGVTDDGMAVATGSWDSFLRIWN......
Celegansgpb2[B5] 330 KCARHSVLYGHENRISCLRTSPDGTAVCSASWDCTIRIWA......
DrosophilaGbeta5[B5] 319 KSERVCLLYGHENKVSCVQVSPDGTALSTGSWDYTIRVWA......
HumanGNB5[B5] 356 KGSRVSILFGHENRVSTLRVSPDGTAFCSGSWDHTLRVWA......
MouseGnb5[B5] 356 KGSRVSILFGHENRVSTLRVSPDGTAFCSGSWDHTLRVWA......

```
                                     α1                             α2
CattleGNG2 [G2]
                         1        10         20          30
CattleGNG2 [G2]   ......MASNNTASIAQARKLVEQLK..........MEANIDRIKV........SKAAAD
1191              M........NKSSTVSNDRKLSKNIKKEAKENFVNRLQIKINKKQINSQLADKLDETTEM
1090              .....MQPDKVIRKMAELNKEISILK..........NNLRVKRIPV........SQAVQE
2571              ........MPAKRDVAKAMVELETLR..........ESSQVKRLPL........SVTLKE
1237              ...........MSGISQQKKLVEQLR..........AEAAINRRPV........SASVQE
2883              MSAAATEENPRQNEIDMKARELEYLK..........NILKLERLQL........SKTIKD
HumanGNGT1 [Gt1]  MP...VINIEDLTEKDKLKMEVDQLK..........KEVTLERMLV........SKCCEE
HumanGNG2 [G2]    ......MASNNTASIAQARKLVEQLK..........MEANIDRIKV........SKAAAD
HumanGNG3 [G3]    MK..GETPVNSTMSIGQARKMVEQLK..........IEASLCRIKV........SKAAAD
HumanGNG4 [G4]    MK..EGMSNNSTTSISQARKAVEQLK..........MEACMDRVKV........SQAAAD
HumanGNG5 [G5]    ........MSGSSSVAAMKKVVQQLR..........LEAGLNRVKV........SQAAAD
HumanGNG7 [G7]    ........MSATNNIAQARKLVEQLR..........IEAGIERIKV........SKAASD
HumanGNG8 [G8]    .......MSNNMAKIAEARKTVEQLK..........LEVNIDRMKV........SQAAAE
HumanGNG10 [G10]  ........MSSGASASALQRLVEQLK..........LEAGVERIKV........SQAAAE
HumanGNG11 [G11]  MP...ALHIEDLPEKEKLKMEVEQLR..........KEVKLQRQQV........SKCSEE
HumanGNG12 [G12]  ....MSSKTASTNNIAQARRTVQQLR..........LEASIERIKV........SKASAD
HumanGNG13 [G13]  .........MEEWDVPQMKKEVESLK..........YQLAFQREMA........SKTIPE
HumanRGS7         SPTHTPTPETKPPTEDELQQQIKYWQ..........IQLDRHRLKM........SKVADS

CattleGNG2 [G2]                       TTT
                     40         50         60       ☆70
CattleGNG2 [G2]   LMAYCEAHAKEDPLLTPVP.ASENPFREKKF..FCAIL......................
1191              ILENCNQEQSN.......PSNKEKPKKQKKL..KNLEIEK...IINKNITSFGHSYENIF
1090              LIIYCNENSTHDIWMNP...LIKSPFSSTHWSICGML.......................
2571              LIEFCNTNYKVDFFLNP...MEENPFKSHRKPICSII.......................
1237              MIQYMDENKDKDCLVSGFANKKDNPFQEKGG..CIIL.......................
2883              FMEYCYNNANSDPLLYP...LKENPFLEKKS..CIIL.......................
HumanGNGT1 [Gt1]  VRDYVEERSGEDPLVKGIP.EDKNPFKELKG..GCVIS......................
HumanGNG2 [G2]    LMAYCEAHAKEDPLLTPVP.ASENPFREKKF..FCAIL......................
HumanGNG3 [G3]    LMTYCDAHACEDPLITPVP.TSENPFREKKF..FCALL......................
HumanGNG4 [G4]    LLAYCEAHVREDPLIIPVP.ASENPFREKKF..FCTIL......................
HumanGNG5 [G5]    LKQFCLQNAQHDPLLTGVS.SSTNPFRPQKV..CSFL.......................
HumanGNG7 [G7]    LMSYCEQHARNDPLLVGVP.ASENPFKDKKP..C.IIL......................
HumanGNG8 [G8]    LLAFCETHAKDDPLVTPVP.AAENPFRDKRL..FCVLL......................
HumanGNG10 [G10]  LQQYCMQNACKDALLVGVP.AGSNPFREPRS..CALL.......................
HumanGNG11 [G11]  IKNYIEERSGEDPLVKGIP.EDKNPFKE.KG..SCVIS......................
HumanGNG12 [G12]  LMSYCEEHARSDPLLIGIP.TSENPFKDKKT..C.IIL......................
HumanGNG13 [G13]  LLKWIEDGIPKDPFLNPDL.MKNNPWVEKGK..CTIL.......................
HumanRGS7         LLSYTEQYLEYDPFLLPP..DPSNPWLSDDT..TFWELEASKEPSQQRVKRWGFGMDEAL

CattleGNG2 [G2]

CattleGNG2 [G2]   ............................................................
1191              ............................................................
1090              ............................................................
2571              ............................................................
1237              ............................................................
2883              ............................................................
HumanGNGT1 [Gt1]  ............................................................
HumanGNG2 [G2]    ............................................................
HumanGNG3 [G3]    ............................................................
HumanGNG4 [G4]    ............................................................
HumanGNG5 [G5]    ............................................................
HumanGNG7 [G7]    ............................................................
HumanGNG8 [G8]    ............................................................
HumanGNG10 [G10]  ............................................................
HumanGNG11 [G11]  ............................................................
HumanGNG12 [G12]  ............................................................
HumanGNG13 [G13]  ............................................................
HumanRGS7         KDPVGREQFLKFLESEFSSENLRFWLAVEDLKKRPIKEVPSRVQEIWQEFLAPGAPSAIN

CattleGNG2 [G2]

CattleGNG2 [G2]   ............................................................
1191              ............................................................
1090              ............................................................
2571              ............................................................
1237              ............................................................
2883              ............................................................
HumanGNGT1 [Gt1]  ............................................................
HumanGNG2 [G2]    ............................................................
HumanGNG3 [G3]    ............................................................
HumanGNG4 [G4]    ............................................................
HumanGNG5 [G5]    ............................................................
HumanGNG7 [G7]    ............................................................
HumanGNG8 [G8]    ............................................................
HumanGNG10 [G10]  ............................................................
HumanGNG11 [G11]  ............................................................
HumanGNG12 [G12]  ............................................................
HumanGNG13 [G13]  ............................................................
HumanRGS7         LDSKSYDKTTQNVKEPGRYTFEDAQEHIYKLMKSDSYPRFIRSSAYQELLQAKKKSGNSM
```
