# Supplementary material for: Heterotrimeric G proteins regulate planarian regeneration and behavior
Source: Genetics. 2023 Feb 10;223(4):iyad019. doi: 10.1093/genetics/iyad019 (PMC10078920; doi:10.1093/genetics/iyad019)
Supplement: iyad019_Supplementary_Data [file iyad019_supplementary_data.zip › Supplemental_Figure_Legends_GENETICS-2022-305416.docx]

**Supplemental Figure Legends**

**Figure S1. Additional G protein subunit expression patterns.** Representative images of G protein subunit ISH that were not included in Figure 1 of the main text. Images are grouped by the most visually enriched tissue type. Scale bars = 200μm.

**Figure S2. Quantitative assessment of RNAi efficiency and specificity. (A)** Relative expression levels of *Gαi2, Gβ1-4b, Gγ-like1, Gαq1,* and *Gβ1-4a* after targeting the corresponding gene with RNAi, measured by RT-qPCR. Values are grouped by experiment. **(B)** Relative expression levels of *Gβ1-4a* and *Gβ1-4b* after targeting the opposite gene with RNAi. **(C)** Relative expression levels of *Gβ1-4a* and *Gαq1* after targeting the opposite gene with RNAi. Error bars represent SEM. Differences in sample means were statistically analyzed with Unpaired T-Tests. ** = P-value ≤ 0.01. *** = P-value ≤ 0.0005. **** = P-value ≤ 0.0001. 7 dpa RT-qPCR experiments throughout this work utilize the *Gαq1(RNAi)* or *Gβ1-4a(RNAi)* cDNA used to produce the data displayed here.

**Figure S3. Additional behavior phenotypes. (A)** Image stills from videos capturing locomotion displayed by intact control, *Gas2(RNAi)*, *Gβx2(RNAi)*, and *gcr052(RNAi)* animals. **(B)** Results from quantification of average velocity over a 40 second timespan in intact knockdown animals. Each dot represents an individual animal, and mean and standard deviation are displayed. **(C)** Image stills from videos taken during the long-term assay in Figure S6 capturing locomotion displayed by intact control, *Gaq1(RNAi)*, and *Gβ1-4a(RNAi)* animals after 28 days of RNAi*.* **(D)** Results from negative phototaxis assays, displaying the percentage of animals residing in the uncovered area after each minute. Data are grouped by experiment. Poor animal health prevented the analysis of negative phototaxis in *Gβ1-4a(RNAi)* animals. **(E)** Image stills from videos capturing locomotion displayed by intact control, *Gas2:Gβx2*, *Gβx2:gcr052*, and *Gas2:gcr052* double knockdown animals. **(F)** Results from quantification of average velocity over a 40 second timespan in double knockdown animals, displayed as mean and standard deviation. Differences in both regenerating and double knockdown experiments were analyzed with Brown-Forsythe and Welch ANOVA with multiple comparisons. *** = Adjusted P-value ≤ 0.0005. **** = Adjusted P-value ≤ 0.0001. Scale bars = 2 mm.

**Figure S4. Additional regeneration phenotypes. (A)** Representative images showing animals treated with RNAi 7 days after tail amputation. **(B)** Bar graph with quantification of tail blastema/body ratios, using the quantification method referred to in Main Figure 3 applied to blastemas instead of brains. Results are displayed as mean and standard deviation. Differences were statistically analyzed with One-Way ANOVA with multiple comparisons. * = Adjusted P-value ≤ 0.05. ** = Adjusted P-value ≤ 0.005. *** = Adjusted P-value ≤ 0.0005. **(C)** Representative images showing asymmetrical and notched tail blastemas in *Gαq1(RNAi)* and *Gβ1-4a(RNAi)* animals 7 dpa. Representative images and quantification of brain regeneration in **(D)** combinatorial RNAi of all predicted Gγ-like subunits and **(E)** *Gαq1(RNAi)* and *Gβ1-4a(RNAi)* animals 14 dpa. Bar graphs are displayed as mean and standard deviation. Differences were analyzed with Unpaired T-Tests with Welch’s correction in (D). **** = P-value ≤ 0.0001. Differences were analyzed with Brown-Forsythe and Welch ANOVA with multiple comparisons in (E). * = Adjusted P-value ≤ 0.05. **** = Adjusted P-value ≤ 0.0001. Scale bars = 200μm.

**Figure S5. *Gαq1* and *Gβ1-4a* are upregulated after injury but are not required for described wound response programs. (A)** Representative images of *Gαq1* and *Gβ1-4a* ISH in untreated animals 6 hpa and 3 dpa. Example image of *ChAT* ISH at 3 dpa, for reference. **(B)** Representative images of *inhibin* and *jun-1* ISH in *Gαq1(RNAi)* and *Gβ1-4a(RNAi)* animals 6 hpa. Zoomed images are the red-dashed boxes in the top, whole-body images. **(C)** Relative expression levels of wound response markers 6 hpa, measured by RT-qPCR. Knockdown strengths for each target gene are included. Error bars represent SEM. Differences in *Gαq1* or *Gβ1-4a* transcripts were measured with Unpaired T-Tests. Differences in wound response markers were analyzed with One-Way ANOVA with multiple comparisons. * = Adjusted P-value ≤ 0.05. **** = P-value ≤ 0.0001. Scale bars = 200μm.

**Figure S6. *Gβ1-4a* likely influences mitosis in a cell non-autonomous manner and promotes survival.** *Smedwi-1* and *Gβ1-4a* dFISH showing **(A)** the head region of intact, untreated animals or **(B)** the head-facing region of amputated animals 24 hpa. Yellow arrowheads indicate examples of cells enriched for each marker, and the yellow box highlights the close association between some *Smedwi-1^+^* and *Gβ1-4a^+^* cells near the amputation site. **(C)** Homeostatic roles for *Gαq1* and *Gβ1-4a* were determined through long-term RNAi paradigms. **(D)** Growth curve data showing mean animal length of 10-12 animals/RNAi treatment over time, displayed with standard deviation. Differences were analyzed with One-Way ANOVA with multiple comparisons for timepoints with values for all three RNAi treatments and an Unpaired T-Test for the last timepoint with only control and *Gβ1-4a(RNAi)*. **** = P-value ≤ 0.0001. **(E)** Survival curve showing the relative percentage of surviving animals after each week of RNAi. **(F)** Representative images of homeostatic phenotypes on day 37 of RNAi. **(G)** The RNAi paradigm used to observe *Smedwi-1* expression after longer-term RNAi. *Gβ1-4a(RNAi)* animals were fixed 1 week earlier due to highly penetrant lysing. **(H)** Representative images of Smedwi-1 ISH in *Gαq1(RNAi)* and *Gβ1-4a(RNAi)* after 42 or 35 days of RNAi. Animals were not amputated, but 8/12 *Gαq1(RNAi)* animals fissioned and 2/8 *Gβ1-4a(RNAi)* animals showed head lysis. Scale bars = 200μm.

**Figure S7. Additional characterization of polarity in *Gαq1(RNAi)* and *Gβ1-4a(RNAi)* animals. (A)** Representative images of *sFRP-1* ISH in animals treated with RNAi targeting *Gαq1* or *Gβ1-4a* 7 dpa. **(B)** Representative images of *notum* ISH in the posterior of *Gβ1-4a(RNAi)* animals 7 days after tail amputation. The green arrowhead indicates potential expression of *notum* in the regenerating tail region in 1/10 animals. **(C)** Representative images of *ChAT* ISH in *Gβ1-4a(RNAi)* animals 14 days after tail amputation. The red dashed lines indicate the amputation site and the black arrowheads indicate expression in the pharynx. Representative images of *foxD* ISH in regenerating heads at **(D)** 3 dpa and **(E)** 7 dpa in *Gαq1(RNAi)* and *Gβ1-4a(RNAi)* animals. Magenta arrowheads indicate absence of *foxD* expression in anterior pole domains. **(F)** Relative expression of anterior pole markers 7 dpa, measured by RT-qPCR and displayed as mean and standard error. Data for *foxD* was analyzed with Unpaired T-Test. * = P-value ≤ 0.05. Data for *sFRP-1* and *notum* were analyzed with One-way ANOVA with multiple comparisons. * = Adjusted P-value ≤ 0.05. ** = Adjusted P-value ≤ 0.005. Scale bars = 200μm.

**Figure S8. *Gαq1* and *Gβ1-4a* regeneration phenotypes are rescued to varying degrees by co-targeting of posterior-promoting pathway signals. (A)** Bar graphs showing results from quantification of brain/body ratios in rescue experiments with *Gαq1(RNAi)* animals, including *bmp4* controls. Error bars represent standard deviations. **(B)** Representative images showing *ChAT* expression in 7 dpa regenerated animals from rescue experiments in (A). **(C)** Relative expression levels of *Gαq1* in animals from rescue experiments, measured by RT-qPCR and displayed as mean and standard error. **(D)** Bar graph showing results from quantification of brain/body ratios from rescue experiments performed alongside animals destined for RT-qPCRs in (C). **(E)** Representative images showing *ChAT* expression 7 dpa from rescue experiments in (D). **(F)** Brain/body ratios from rescue experiments performed with *Gβ1-4a(RNAi)*, displayed as mean and standard deviation*.* **(G)** Representative images showing *ChAT* expression 7 dpa from rescue experiments in (F). Differences in sample means for (A), (D), and (F) were statistically analyzed with Brown-Forsythe and Welch ANOVA with multiple comparisons. Differences in transcript abundance in (C) were analyzed with One-Way ANOVA with multiple comparisons. ** = Adjusted P-value ≤ 0.006. *** = Adjusted P-value ≤ 0.0006. **** = Adjusted P-value ≤ 0.0001. Scale bars = 200μm (B and E) and 500μm (G).

**Figure S9. Many heterotrimeric G protein subunit-encoding genes in *S. mediterranea* display expression enriched in the brain branches.** Images from the head region of ISH images showing G protein subunit gene expression in brain branches. Staining patterns displayed include a variety of brain branch patterns, ranging from broad to narrow expression in these structures. Scale bars = 200μm.

**File S1. Alignment and secondary structure predictions for *Schmidtea mediterranea* Gα, Gβ, and Gγ** **class subunits.** (Page 1) Secondary structure assignments are taken from *Rattus norvegicus* Gαi1, *Bos taurus* Gβ1, and *Bos taurus* Gγ2 (PDB ID: 1GP2). For Gα class subunits, triangles denote residues that contact Gβ, and stars represent the residues involved in GTP hydrolysis and switch II residues (Lys209, Trp211, Ile212, and Phe215) that interact with Gβ1. Highly truncated *S. mediterranea* Gα sequences were excluded (see File S3). (Page 5) For Gβ class subunits, triangles denote residues that contact Gγ, diamonds represent regions where contacts form with the switch regions in Gα, and stars indicate the Gβ1 residues (Trp99, Asp228, and Asp246) that interact with the Gαi1 switch II helix. (Page 8) For Gγ class subunits, triangles denote residues that contact Gβ and the star represents the Gγ prenylation site.

**File S2. Phylogenetic trees for *S. mediterranea* Gα, Gβ, and Gγ** **class subunits.** Branch support values displayed in red.

**File S3. Alignment and secondary structure predictions for *S. mediterranea* Gα class subunits including truncated sequences.** Secondary structure assignments are taken from *Rattus norvegicus* Gαi1 (PDB ID: 1GP2).

**Table S1. Summary of *S. mediterranea* heterotrimeric G protein subunits.** The table includes gene identifications, enriched clusters from single sell sequencing (Fincher et al., 2018), ISH descriptions, RNAi phenotype summaries, and homology details for all genes from this study.

**Table S2. Overlap between roles in behavior and roles in regeneration.** The table includes the genes significant for behavior and/or regeneration with details of the phenotypes observed after amputation during the regeneration screens, during short-term homeostatic RNAi, and during long-term homeostatic RNAi.

**Table S3. GPCRs potentially expressed with key heterotrimeric G protein-encoding genes.** The table provides the GPCR-encoding genes that are enriched in the same cell clusters as heterotrimeric G protein subunits of interest from single cell sequencing data (Fincher et al., 2018).

**Table S4. Raw data of brain regeneration screens displayed in Figure 3.** The table shows how the values were calculated for the brain regeneration bar graph in Figure 3. All other brain regeneration data in this work utilized the same method of quantification, based on (Roberts-Galbraith et al., 2016). Inside the cyan box are the area measurements (for either the brain or the body) from *ChAT* (Nishimura et al., 2010) ISH images, generated by tracing the structures in FIJI (Schindelin et al., 2012) after setting the scale in the software using the scale bar reference on the image. Each animal was imaged and measured individually, and the animal number associated with each set of measurements is shown on the left. Each structure is traced three times, then the values of the three traces are averaged in the subsequent Excel cell. Inside the orange box are the values demonstrating the percentage of the animal’s body area that contains the brain. These values are generated by dividing the average brain area by the average body area and multiplying by 100. The average of the brain/body ratios is found below the individual animal brain/body ratio values. Inside the magenta box are additional descriptive values of the data, including the sample size and standard deviation. Inside the green box are the values that we display in brain regeneration bar graphs and use for statistical analysis in Prism - GraphPad Version 7.0 software (GraphPad Software, San Diego, CA). Each raw value is divided by the average of the control and multiplied by 100 to generate a percent change between the *control(RNAi)* brain/body ratios and *experimental(RNAi)* brain/body ratios. Each RNAi treatment is displayed on its own sheet and matched with the control values of its corresponding experiment.

**Table S5. Reference protein sequences used for classification of *S. mediterranea* heterotrimeric G protein subunits.**

**Table S6. Primer sequences used for cloning and RT-qPCR in this study.** We occasionally cloned a larger section of the transcript to optimize ISH clarity for genes of interest. In these cases, the function of each primer set is indicated in parenthesis.

**Video S1. *Gαs1* is required for planarian flipping behavior.** A 10 second video showing different reactions when placed dorsal side down displayed by *Ctrl(RNAi)* and *Gαs1(RNAi)* animals 7 dpa, on day 28 of RNAi. Playback set to 8X speed.

**Video S2. Behavior displayed by intact control, *Gαs2(RNAi), Gβx2(RNAi),* and *gcr052(RNAi)* animals.** A 10 second video showing locomotion of intact knockdown animals. Playback set to 20X speed. Each grid square = 13x13 mm.

**Video S3. Behavior displayed by regenerating control, *Gαs2(RNAi), Gβx2(RNAi),* and *gcr052(RNAi)* animals.** A 10 second video showing locomotion of knockdown animals 10 dpa. Playback set to 20X speed. Each grid square = 13x13 mm.

**Video S4. Behavior displayed by intact control, *Gαq1(RNAi)*, and *Gβ1-4a(RNAi)* animals.** A 10 second video showing locomotion of knockdown animals on day 28 of the long-term RNAi paradigm in Supplemental Figure S6C. Playback set to 20X speed. Each grid square = 13x13 mm.

**Video S5. Behavior displayed by intact double knockdown animals.** A 10 second video showing locomotion of intact control, *Gαs2:Gβx2(RNAi)*, *Gαs2:gcr052(RNAi)*, and *Gβx2:gcr052(RNAi)* animals. Playback set to 20X speed. Each grid square = 13x13 mm.
