# Supplementary figures and images for: Heterotrimeric G proteins regulate planarian regeneration and behavior
Source: Genetics. 2023 Feb 10;223(4):iyad019. doi: 10.1093/genetics/iyad019 (PMC10078920; doi:10.1093/genetics/iyad019)

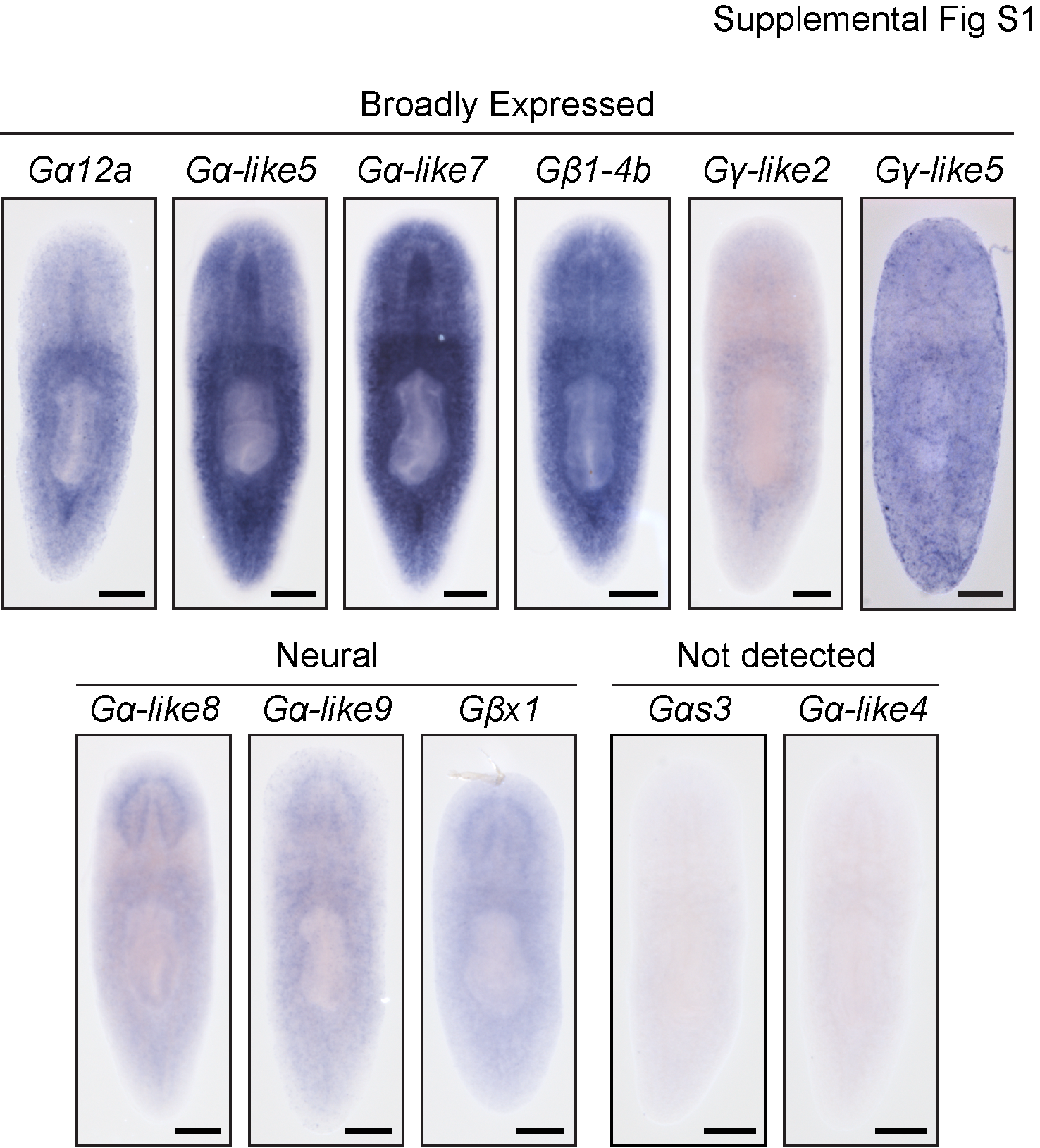

Supplement: iyad019_Supplementary_Data [file iyad019_supplementary_data.zip › Figure_S1_GENETICS-2022-305416.tif]

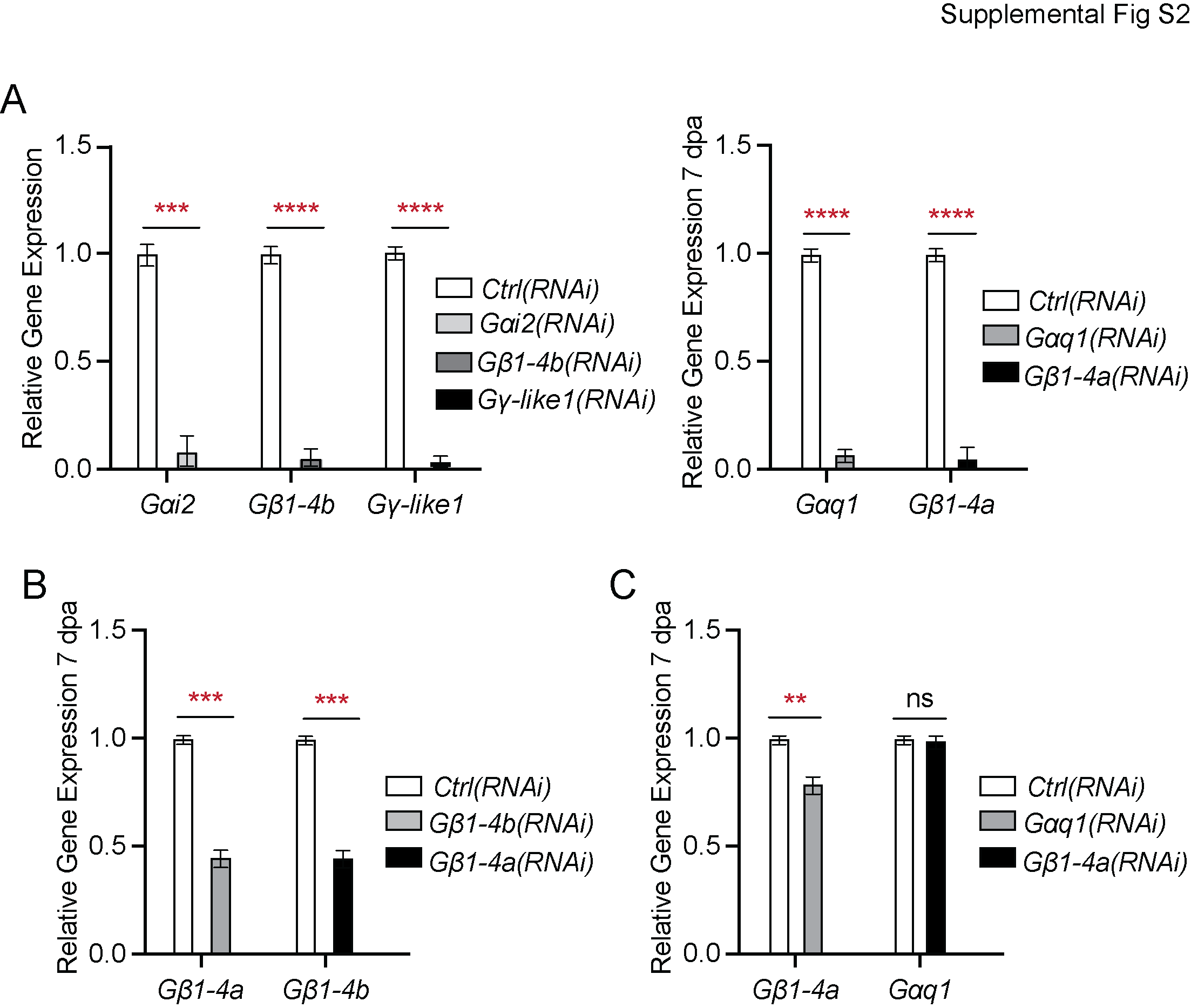

Supplement: iyad019_Supplementary_Data [file iyad019_supplementary_data.zip › Figure_S2_GENETICS-2022-305416.tif]

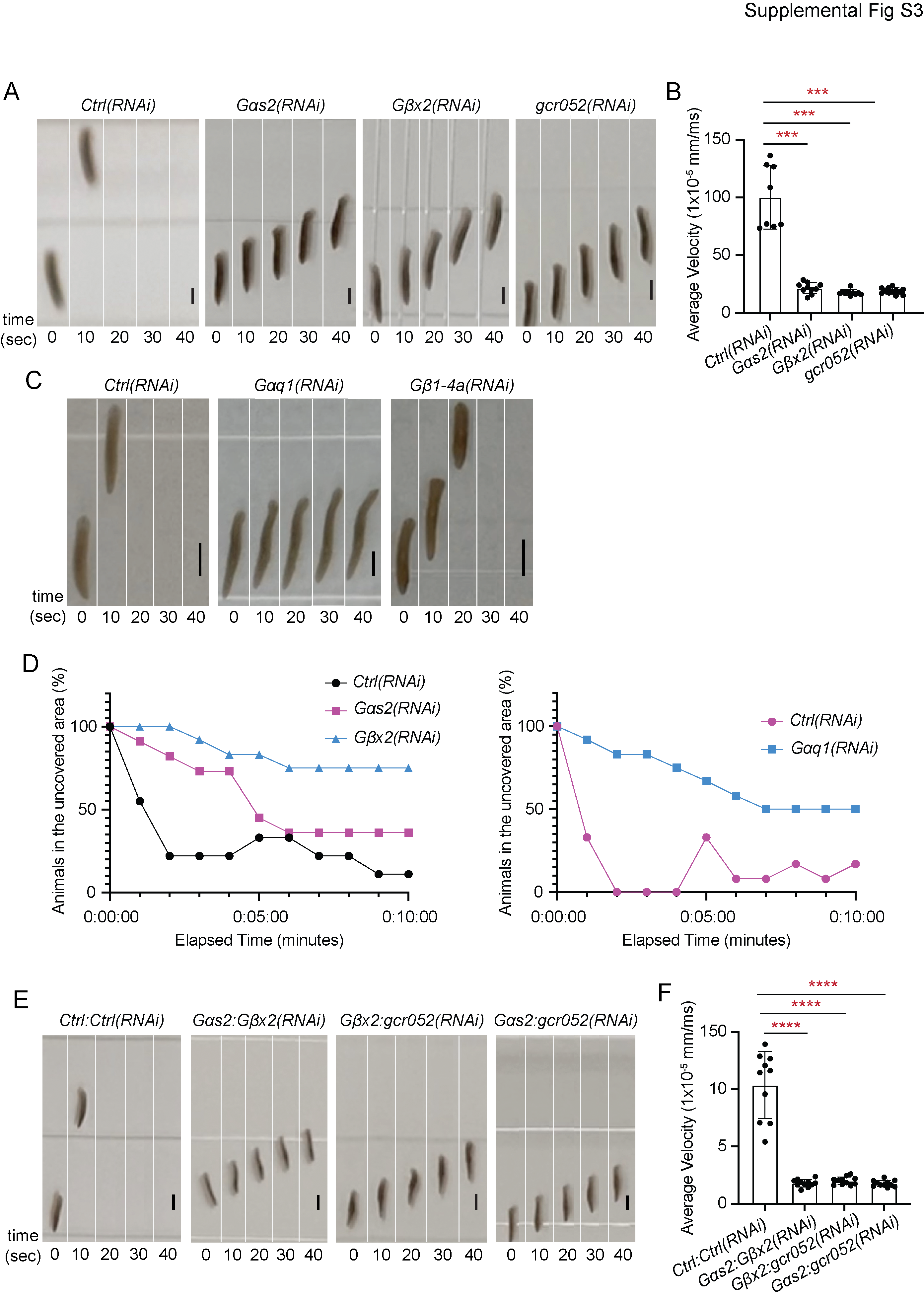

Supplement: iyad019_Supplementary_Data [file iyad019_supplementary_data.zip › Figure_S3_GENETICS-2022-305416.tif]

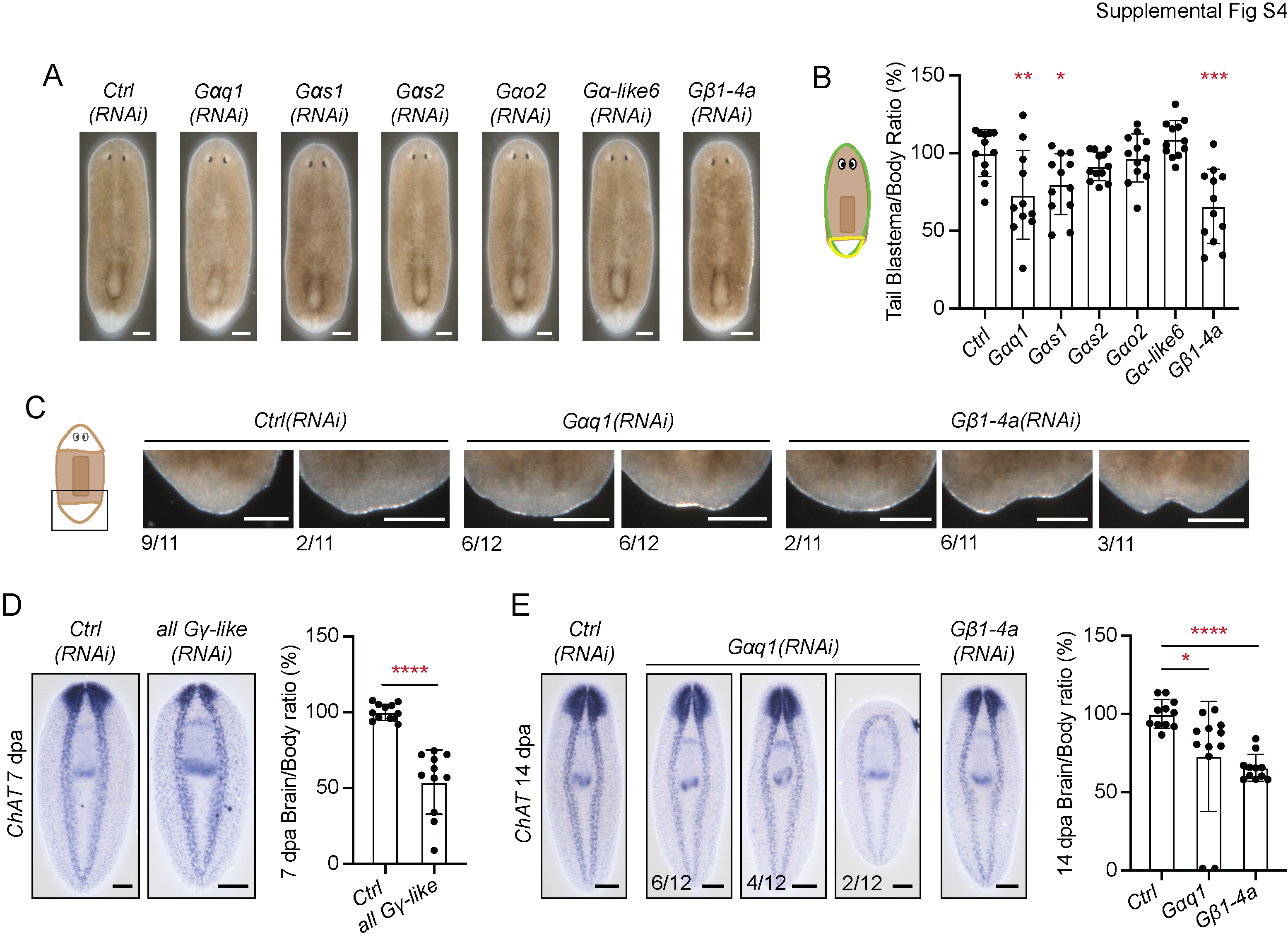

Supplement: iyad019_Supplementary_Data [file iyad019_supplementary_data.zip › Figure_S4_GENETICS-2022-305416.tif]

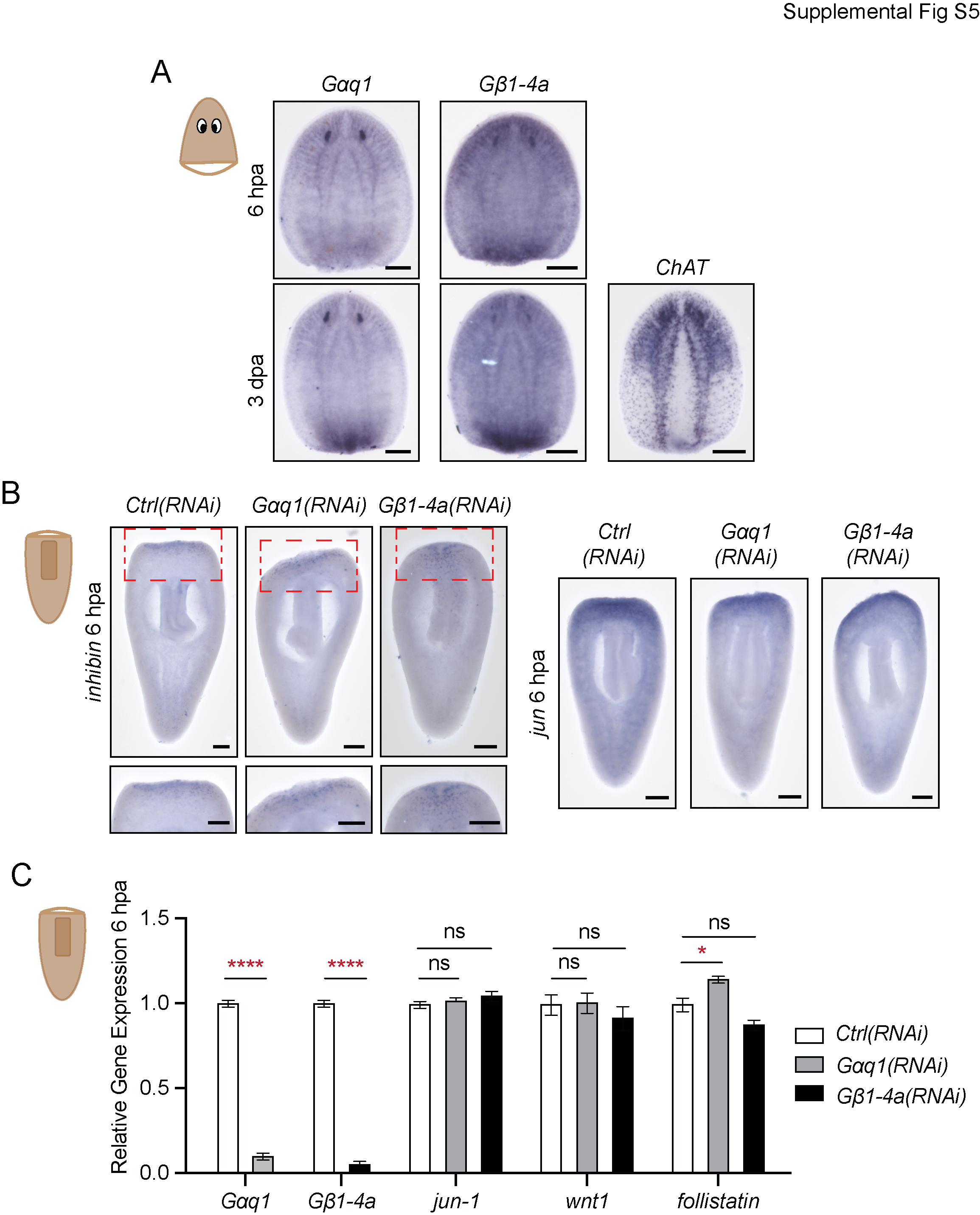

Supplement: iyad019_Supplementary_Data [file iyad019_supplementary_data.zip › Figure_S5_GENETICS-2022-305416.tif]

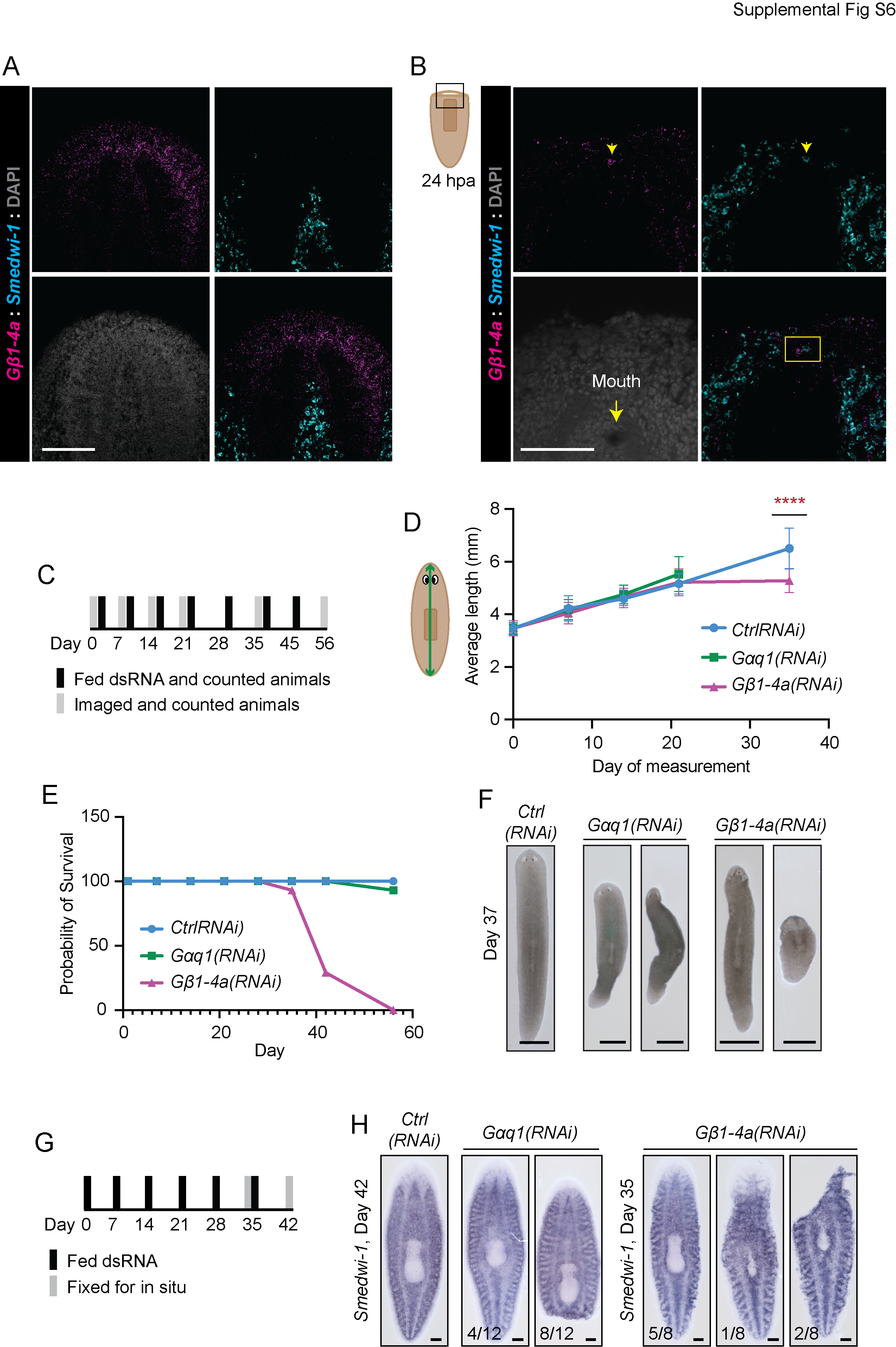

Supplement: iyad019_Supplementary_Data [file iyad019_supplementary_data.zip › Figure_S6_GENETICS-2022-305416.tif]

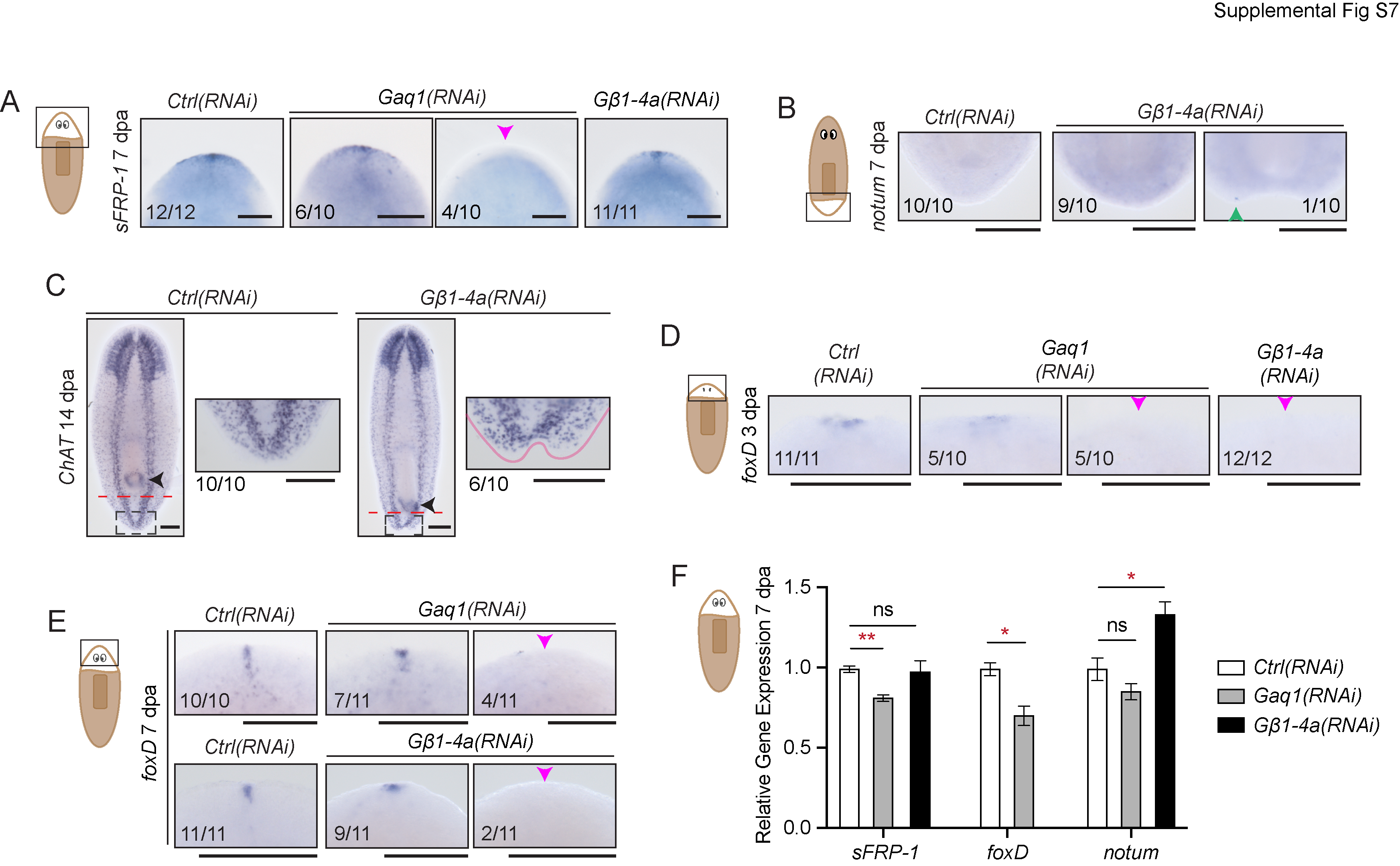

Supplement: iyad019_Supplementary_Data [file iyad019_supplementary_data.zip › Figure_S7_GENETICS-2022-305416.tif]

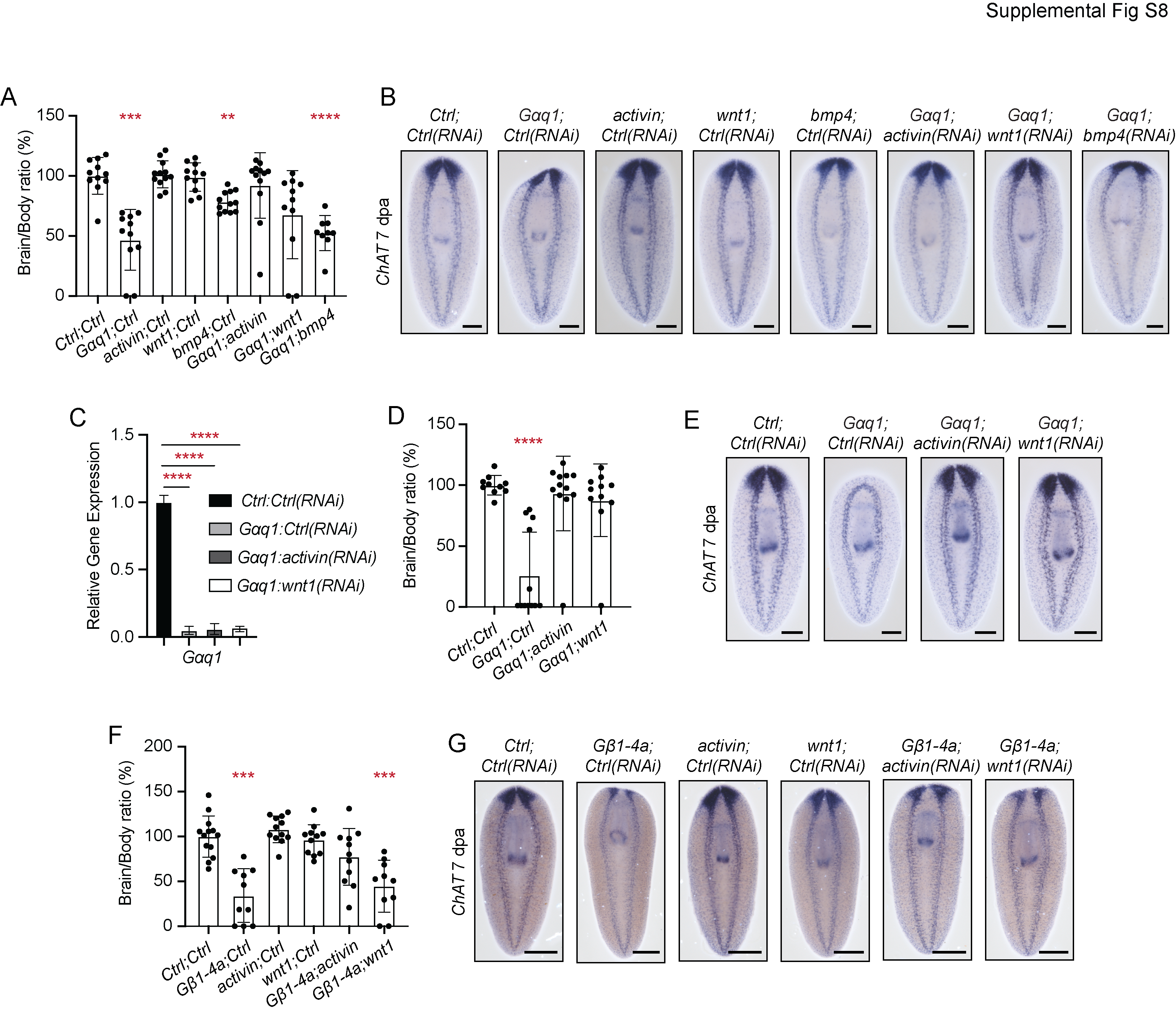

Supplement: iyad019_Supplementary_Data [file iyad019_supplementary_data.zip › Figure_S8_GENETICS-2022-305416.tif]

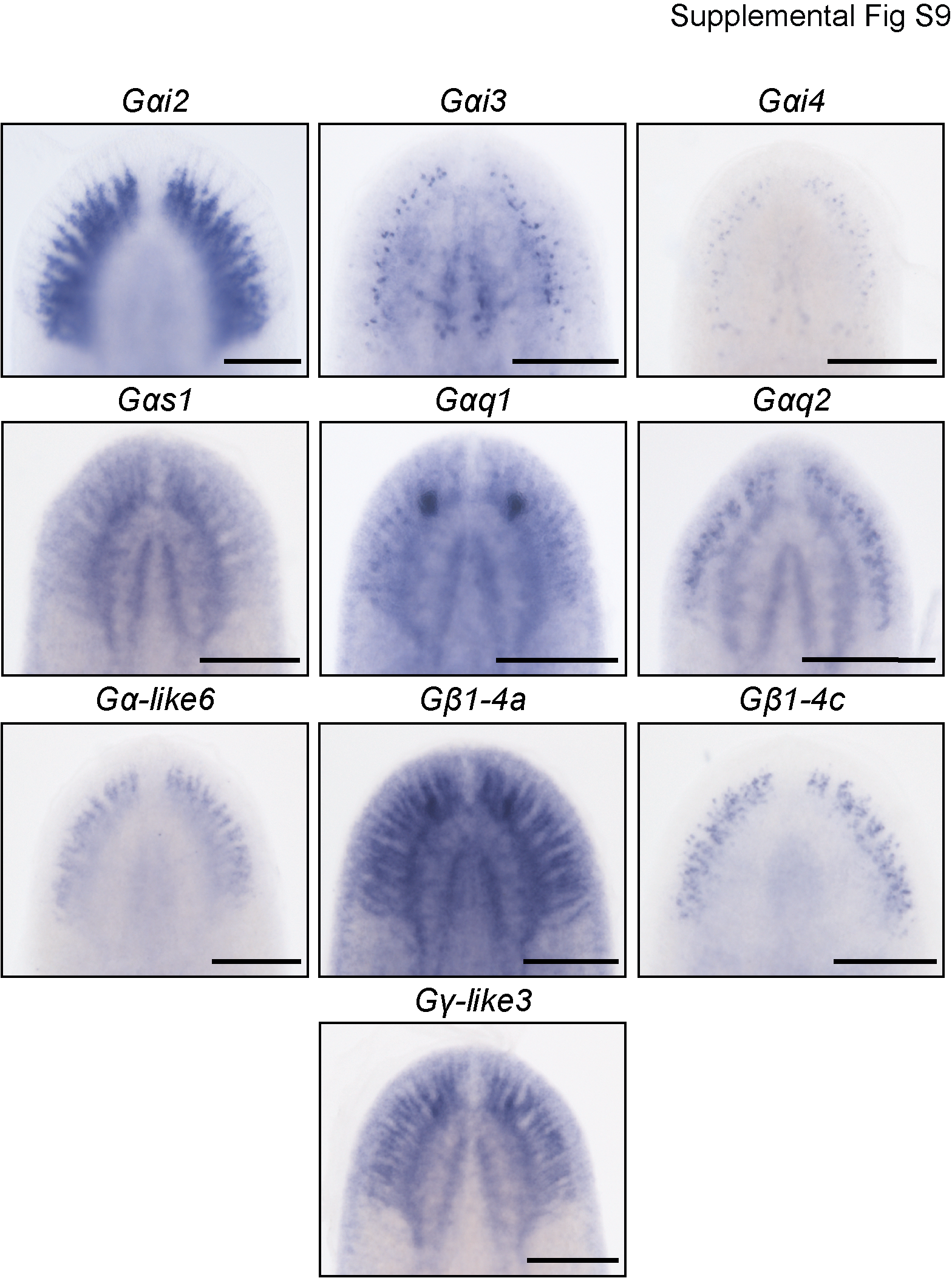

Supplement: iyad019_Supplementary_Data [file iyad019_supplementary_data.zip › Figure_S9_GENETICS-2022-305416.tif]

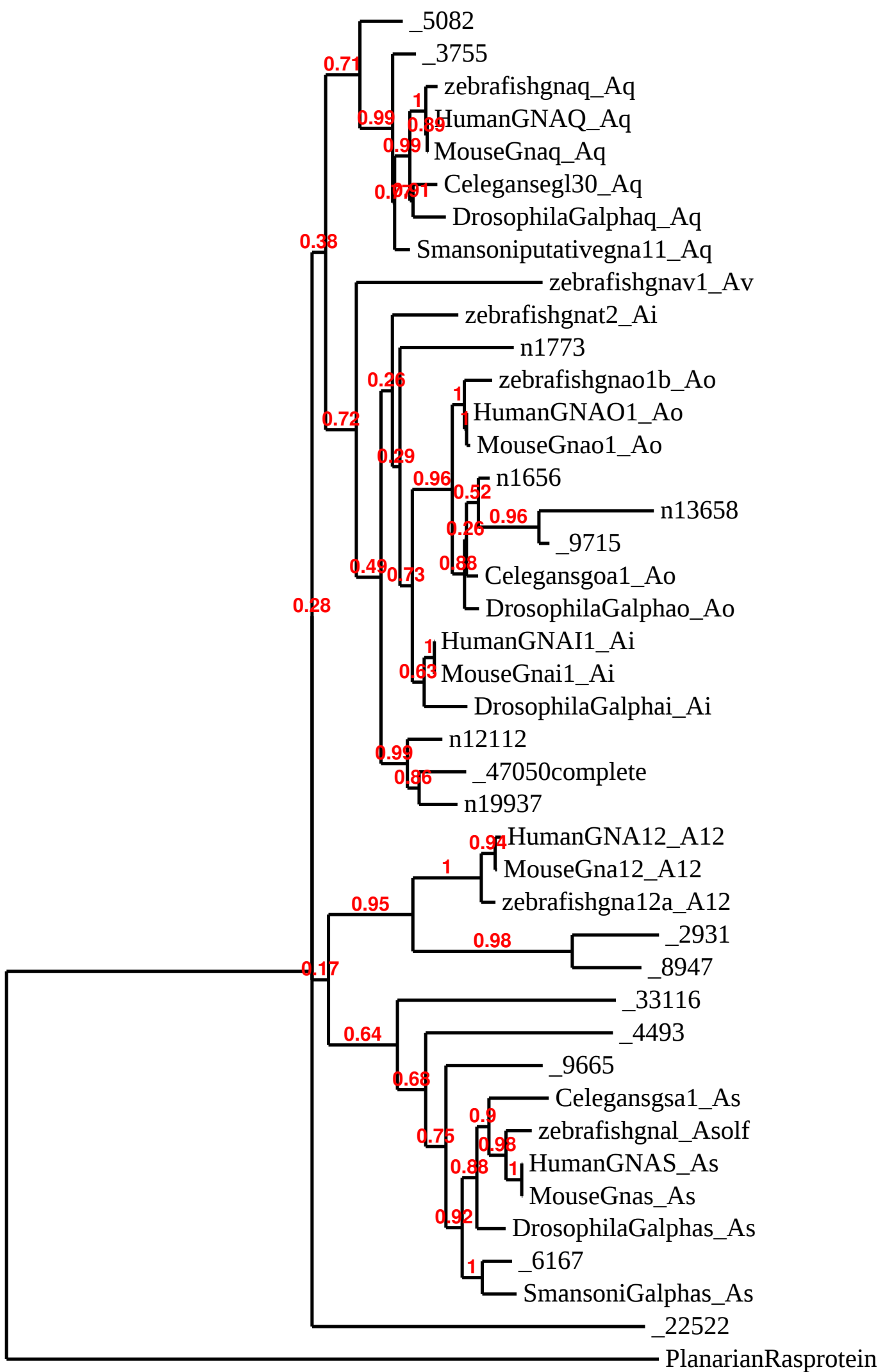

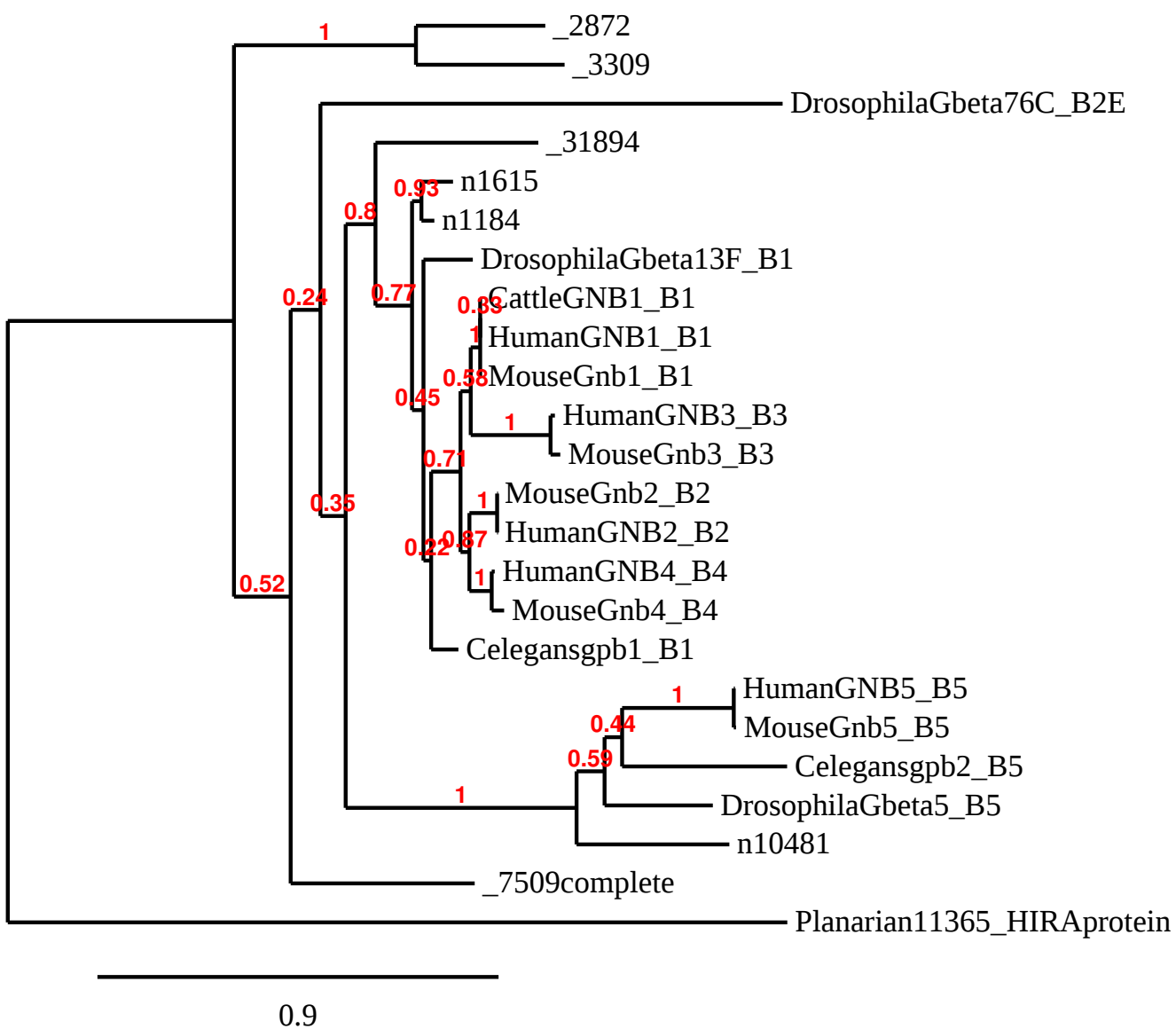

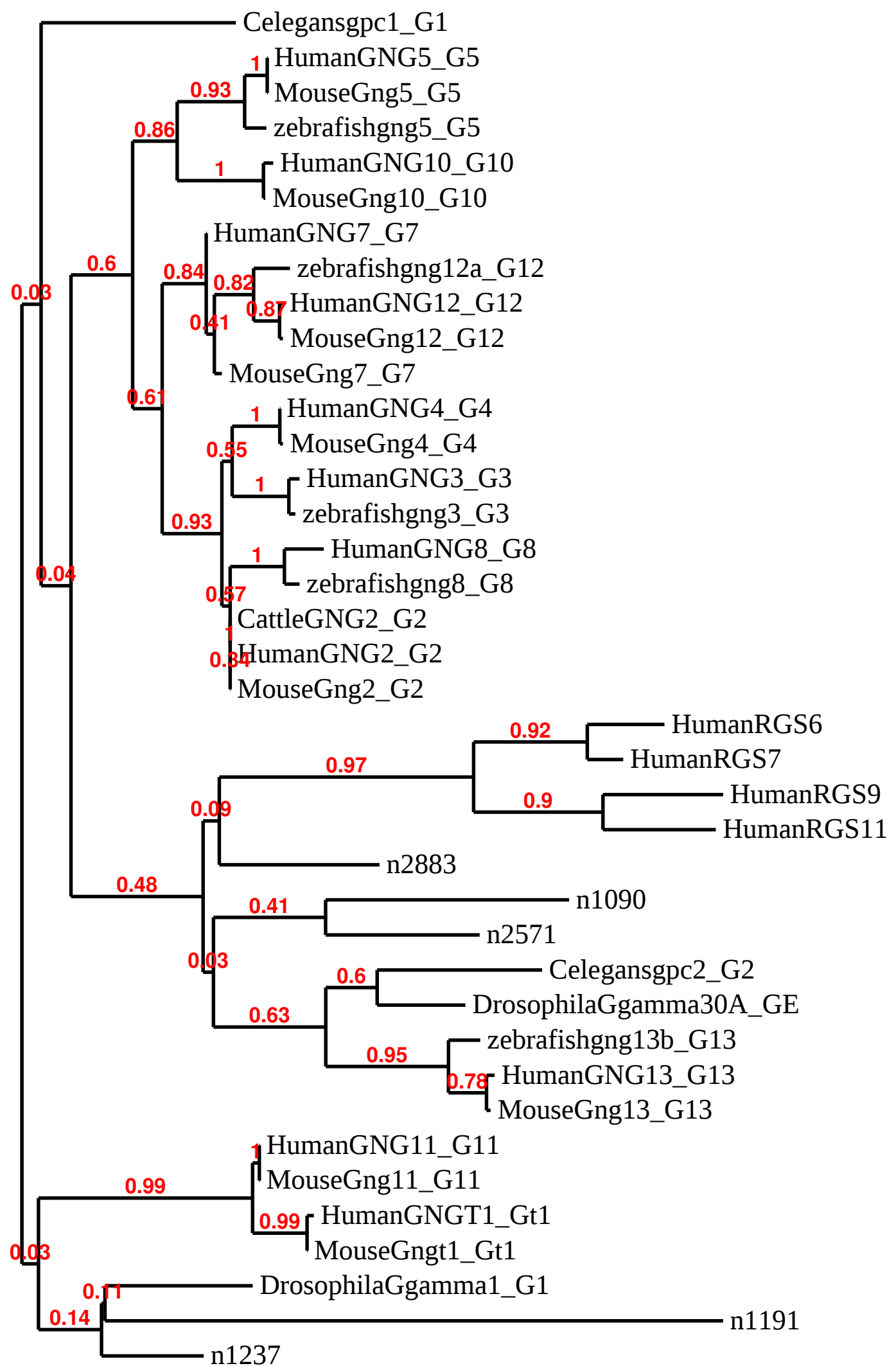

Supplement: iyad019_Supplementary_Data [file iyad019_supplementary_data.zip › File_S2_GENETICS-2022-305416.pdf]
